# Genome-wide analysis of the C2H2-ZFP gene family in *Stevia rebaudiana* reveals involvement in abiotic stress response

**DOI:** 10.1038/s41598-024-56624-y

**Published:** 2024-03-14

**Authors:** Shahla Nikraftar, Rahman Ebrahimzadegan, Mohammad Majdi, Ghader Mirzaghaderi

**Affiliations:** Department of Plant Production and Genetics, Faculty of Agriculture, University of Kurdistan, P. O. Box 416, Sanandaj, Iran

**Keywords:** C2H2-ZFP, Gene family, Transcription factor, Stevia, Genetics, Agricultural genetics

## Abstract

Stevia (*Stevia rebaudiana* Bertoni) is a natural sweetener plant that accumulates highly sweet steviol glycosides (SGs) especially in leaves. Stevia is native to humid areas and does not have a high tolerance to drought which is the most serious abiotic stress restricting its production worldwide. C2H2 zinc finger proteins (C2H2-ZFPs) are a group of well-known transcription factors that involves in various developmental, physiological and biochemical activities as well as in response to abiotic stresses. Here we analyzed C2H2-ZFP gene family in stevia and identified a total of 185 putative SrC2H2-ZF proteins from the genome sequence of *S. rebaudiana*. We further characterized the identified C2H2-ZF domains and their organization, additional domains and motifs and analyzed their physicochemical properties, localization and gene expression patterns. The cis-element analysis suggested multiple roles of SrC2H2-ZFPs in response to light, phytohormone, and abiotic stresses. In silico analysis revealed that the stevia *C2H2-ZFP* genes are interactively expressed in different tissues and developmental stages and some *C2H2-ZFP* genes are involved in response to drought stress. This study provides a background for future exploration of the functional, and regulatory aspects of the *C2H2-ZFP* gene family in *S. rebaudiana*.

## Introduction

Stevia (*Stevia rebaudiana* Bertoni) is a perennial short-day and medicinal herb of the Asteraceae family that is native to Brazil and Paraguay. It grows near water sources (high relative humidity) and prefers sandy, slightly acidic, and loamy soil with high humus content and good drainage^1,2^. Drought is the most serious abiotic stress restricting stevia production worldwide. Although stevia cannot tolerate drought, it grows in wide temperatures from 15 to 38 °C^3^. It is well known for the accumulation of low-calorie content and highly sweet steviol glycosides (SGs) especially in leaves. Despite the surging popularity of SGs, limitations in *S. rebaudiana* cultivation hinder widespread adoption. To bridge this gap and secure product quality, probing insights into stevia's growth, environmental resilience, and abiotic stress response is of great importance.

Transcription factors control the expression of their target genes by binding to specific DNA motifs. In plants, transcription factors are key regulators of various developmental processes and responding to environmental stresses^4^. In *S. rebaudiana*, zinc finger (ZF) transcription factors may modify the expression of genes that might be involved in stevioside biosynthesis^5^. Additionally, C2H2-ZFPs may contribute to stevia's resilience, helping it adapt to environmental stresses like drought and salinity^6^. ZF proteins represent a diverse family of transcription factors characterized by the presence of one or more zinc-stabilized structural motifs known as zinc fingers. These finger-like domains fold around a single zinc ion (Zn2^+^) coordinated by conserved cysteine (Cys) and histidine (His) residues, generating distinct subclasses based on their specific amino acid composition. Zinc finger proteins (ZFPs) play a pivotal role in regulating gene expression by binding to specific DNA sequences within the major groove of the double helix. Each zinc finger domain, functioning as an independent DNA recognition module, recognizes a short, three-nucleotide sequence with high specificity^7,8^. This modularity allows for combinatorial binding of multiple zinc fingers, enabling ZFPs to target a wide array of DNA sequences with exquisite precision. Hence, ZFPs mediate regulating gene expression and are involved in a wide range of biological processes, including development, differentiation, and stress response. The ZFP family encompasses nine distinct subclasses classified based on the number and arrangement of cysteine and histidine residues, as well as their secondary structure including Cys2/His2 (C2H2), C3H, C3HC4, C2HC5, C4HC3, C2HC, C4, C6 and C8^9^.

The most prevalent subclass, C2H2-ZF proteins (also known as TFIIIA-type ZFPs), are implicated in various critical plant processes, including growth, development, stress resistance, and hormone signaling^10,11^. The role of C2H2 zinc fingers in gene regulation is to act as transcriptional activators or repressors, depending on the specific protein and the context in which it is functioning. The discovery of the first plant *C2H2-ZFP* gene, EPF1 from petunia, encoding two typical C2H2 zinc finger motifs, serves as a landmark in understanding the role of these versatile proteins in plant biology^12^. Many C2H2-type zinc finger protein genes have since been characterized in model and crop plants^10^. C2H2-ZF proteins with a common pattern of C-X(2–4)-C-X3-F-X5-L-X2-H–X(3–5)-H have 25–30 amino acids with two conserved Cys and two His that coordinate a Zn2 + atom on four sides^8^. Plant C2H2-ZFPs have a longer and more variable linker between their fingers and the QALGGH conserved sequence in the DNA-contacting surface of each finger^13^ compared to animals where the distance between two consecutive fingers is often short, seven or eight amino acids, as exemplified in fruit flies and yeast^14^.

Researchers categorized the plant C2H2-ZF proteins based on the number and arrangement of their C2H2 domains, and the architecture of these domains: Tandem C2H2-ZFPs have closely-spaced C2H2 domains, linked by less than 12 amino acids while dispersed C2H2-ZFPs have C2H2 domains further apart, separated by more than 11 amino acids^15^. Based on domain architecture, each C2H2 domain has been classified into Q-type (X2-C-X2-C-X7-QALGGH-X3-H) harboring a conserved QALGGH sequence), M-type (further divided into M1 to M5 subtypes based on 1 to 5 degraded amino acids in the QALGGH sequence), Z-type (spacing between the second cysteine and first histidine falls within specific ranges for Z1 and Z2 subtypes), D-type (missing the second histidine compared to other types)^16,17^. By using these criteria, the researchers created a detailed classification system for plant C2H2-ZFPs, providing a clearer understanding of their diverse structure and potential functions.

Here, we conducted a structural classification and expression analysis of C2H2 zinc finger protein (C2H2-ZFP) gene family in stevia and analyzed the expression of some *C2H2-ZFP* genes under drought stress.

## Materials and methods

### Identification of SrC2H2-ZFP genes

A flowchart of the analysis steps is presented in Supplementary Figure S1. The *S. rebaudiana* genomeic files were received from Xu et al.^18^, and the multiple sequence alignment (MSA) for C2H2 family was downloaded from the plant transcription factor database (http://planttfdb.gao-lab.org/) which belong to more than 160 species including model plants. The MSA was used it to make a Hidden Markov Model (HMM) profile by hmmbuild option of HMMER package^19^. This HMM profile was used to identify C2H2-ZFPs proteins of *S. rebaudiana*. The identified SrC2H2-ZF proteins were grouped based on the structure of each C2H2-ZFP domain, sequence length between the C2H2 zinc-finger domains, and the availability of additional domains in the protein^15^. SrC2H2-ZFPs were uploaded to the SMART database to distinguish the available domains. The proteins were also inspected manually to confirm the number of C2H2-ZF domains, the position of C2H2-ZF domain, and the space length between two adjacent C2H2-ZFs. The identified SrC2H2-ZF proteins were also assigned to iTAK (http://itak.feilab.net/cgi-bin/itak/online_itak.cgi) to further analyze via C2H2-ZFP domain search^20^. The longest C2H2-ZF protein variant from each gene that was confirmed to have the C2H2-ZFP domain(s) was considered as stevia C2H2-ZF protein and defined as SrC2H2.[type-description]. The confirmed SrC2H2-ZFPs were grouped into Q-, M-, and or D-type according to Alam et al.^16^. Briefly, SrC2H2-ZFPs having the conserved QALGGH motif and the conserved X2-C-X2-C-X7-QALGGH-X3-H pattern in the SrC2H2-ZF domains were classified as Q-type; while proteins with one to five degraded amino acids in the QALGGH motif and specific changes in the spacing between two cysteines (C2) and two histidines (H2) were grouped as M1 to M5. The D-type does not have the second histidine in the SrC2H2-ZF domain compared with the Q and M types. This classification placed, SrC2H2-ZFPs containing a single C2H2-ZF domain into 1i (e.g. 1i-Q, 1i-M). C2H2-ZFPs containing two C2H2-ZF domains were defined as 2i (e.g. 2i-Q and 2i-M). The SrC2H2-ZFPs containing at least two types of n non-tandemly repeated C2H2-ZF domains were classified as ni-Mx. Proteins with three or more than three ZF domains with at least two tandemly repeated ZF domains were presented as t1 and t2, respectively. Two ZF domains separated by up to 11 amino acids were considered tandem duplication^15^.

### Physicochemical characterization of C2H2-ZFPs

Physicochemical properties of each of the identified SrC2H2-ZF proteins including amino acid sequence length (AA), molecular weight (MW), isoelectric point (pI), instability index, aliphatic index, and grand average of hydropathicity were obtained from the ProtParam website (https://web.expasy.org/protparam; Gasteiger et al., 2005). The structure of SrC2H2-ZFP genes was extracted from the *S. rebaudiana* GFF file and the gene structures were presented using the Gene Structure Display Server at http://gsds.cbi.pku.edu.cn^21^. The conserved domains of the SrC2H2-ZF proteins were searched from Pfam^22^ and MEME^23^ web servers and the output tables were visualized in TBtools^24^. Subcellular localization of the SrC2H2-ZFPs was predicted using the Plant-mPLoc webpage^25^.

### Phylogenetic analysis

The identified SrC2H2-ZF protein sequences were aligned using the ClustalOmega argument of msa function from “msa” package^26^ in R (The R Project for Statistical Computing, Vienna, Austria). Then, the alignment was used to obtain a neighbor-joining tree by applying 500 bootstrap replicates using the “ape” package^27^. The resulting newick tree was visualized in R by using the “ggtree” package^28^.

### Evolution of *SrC2H2-ZFP* gene family

Based on the phylogenetic analysis, the gene pairs encoding the most similar proteins were considered as the recently duplicated genes. The coding sequences (CDS) of these gene pairs were extracted and their Ka/Ks (non-synonymous/synonymous substitution) ratios were obtained using the KaKs_Calculator^29^. The divergence time for the duplicated genes was predicted by T = Ks/2λ × 10^–6^, where Ks is the rate of the synonymous nucleotide substitution and λ is the number of substitutions per synonymous site per generation. The λ of 6.5 × 10^−9^ was used according to Yang, Zhang, Yue, Tian and Chen^30^.

### Expression analysis of *C2H2-ZFP* genes

RNAseq data for different developmental stages and tissues (NCBI, BioProject ID PRJNA705537)^31^ and leaf responses to nitrogen deficiency (NCBI, BioProject: PRJNA681456)^18^ of *S. rebaudiana* were downloaded from the SRA database of NCBI. After quality control using FastQC and trimming using Trimmomatic^32^, the low-quality section of reads, the reads of each sample were aligned to the stevia reference genome using the HISAT2 aligner and the aligned reads were assembled using StringTie under its default settings^33^. Extraction of abundance estimates as FPKM (fragments per kilobase of transcript per million mapped reads) values, for the *SrC2H2-ZFP* genes and differential expression analysis were done using the ballgown package^34^. Heatmaps were generated from log2(FPKM + 1) transformed expression values of *SrC2H2-ZFP* genes using R package “pheatmap”. We also extracted the log2 fold changes (log2FC) of the differentially expressed *SrC2H2* genes from the RNAseq results of *S. rebaudiana* roots and leaves under drought (50% of field capacity, one-day hold) and salinity (120 mM) stresses^6^ and presented the barplots of those values using the R package “ggplot2”^35^.

### Cis-regulatory elements of *C2H2-ZFP* genes

The upstream sequences (2-Kb) of *S. rebaudiana* SrC2H2-ZFP genes were extracted from the stevia genome using TBtools^36^. The cis-acting elements of the extracted sequences were predicted using the PlantCARE tool^37^.

### Semi-quantitative reverse transcription PCR (sqRT-PCR)

One-month-old seedlings of *S. rebaudiana* were provided by the Agricultural Research Center of Guilan Province, Iran. Seedlings were transplanted to pots (20 cm diameter and 25 cm height) filled with garden soil and kept in a greenhouse with 22 °C temperature and 16 h of daily light. After two months, we applied drought stress to half of the plants by stopping watering until the leaves wilted while the control plants retained under normal irrigation. We then harvested the second and third leaves from the second lateral stem of both control and stressed plants. The tissues were frozen immediately in liquid nitrogen, and kept at − 80 °C until RNA extraction. Three biological replicates were collected for each of the control and drought treated conditions.

From each frozen stevia leaf sample, total RNA was extracted using acid guanidinium thiocyanate–phenol–chloroform method^38^. The quality of the isolated RNA was evaluated by both agarose gel electrophoresis and NanoDrop spectrophotometry (Supplementary File S1). To prepare for further analysis, 500 ng of RNA per sample was reverse transcribed into cDNA using a TaKaRa kit (Dalian, China). These cDNA products were diluted tenfold and kept frozen at − 20 °C until needed. Three stevia C2H2-ZFP genes with reasonable expression were selected based on the results of the analysis of publicly available RNAseq data as described above and their expression was analyzed by sqRT-PCR in three technical replicates. Specific primers for the selected genes were designed using the online Primer3Plus tool. The forward and reverse primers of each selected *SrC2H2-ZFP* gene were designed from two adjacent exons of their cDNA sequences spanning the interval intron. This would allow to identify undesired PCR products if amplified from genomic DNA contamination. The stevia *Actin* (AF548026.1) was served as an internal reference gene. Band intensities were quantified using ImageJ^39^, and mean expression between control and drought conditions for each gene was compared using a t-test.

### Protein–protein interaction (PPI) network prediction

The Arabidopsis genes orthologous to the stevia C2H2 genes were identified using OrthoVenn3^40^. By referring to the differentially expressed genes in RNAseq results from a stevia drought experiment^6^, the Search Tool for the Retrieval of Interacting Genes (STRING) database (https://string-db.org/) was retrieved for predicting protein–protein interactions (default confidence score ≥ 0.40) using the Arabidopsis orthologs of the stevia C2H2-ZFPs that overexpressed under drought stress.

## Results

### Identification of C2H2-ZFP genes and properties of their proteins

A total of 185 SrC2H2-ZFP genes were identified. In total, 283 transcripts (1–7 transcripts per gene) and on average 1.54 transcripts per gene are encoded. SrC2H2-ZF gene IDs, longest transcripts and protein IDs, assigned gene names, chromosomal positions, general domain architecture and C2H2 domain sequences are presented in Supplementary Dataset S1 and the longest protein sequences of each od SrC2H2-ZF genes are available in Supplementary File S2. The molecular weights of the longest proteins of the *SrC2H2-ZFP* gene transcripts range from 12,448.27 to 158,960.08 Daltons (Da) and the isoelectric point (PIs) ranged from 4.88 to 10.05. All of the SrC2H2-ZF proteins had a negative grand average of hydropathicity indicating the hydrophilic nature of *StC2H2-ZF* proteins. The range of the instability indices of the SrC2H2-ZF proteins was between 32.52 and 75.72. For the majority of the proteins, the instability index was higher than 40.00, suggesting that most SrC2H2-ZF proteins are unstable (Supplementary Dataset S2).

**Figure 1:**
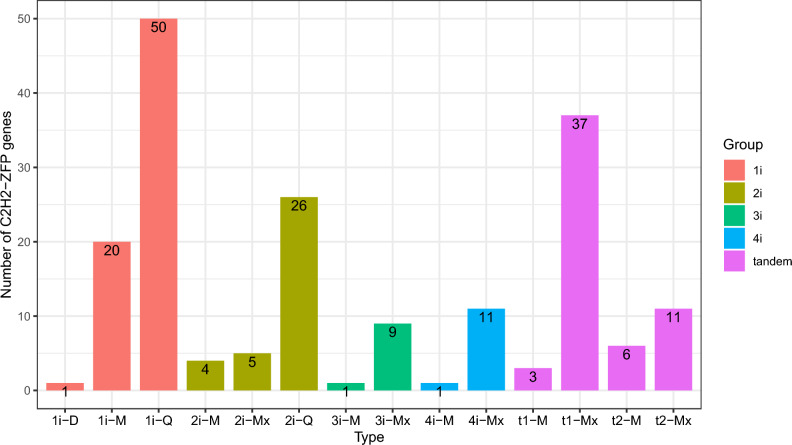
Grouping of 185 SrC2H2-ZF proteins of *S. rebaudiana* based on the number and pattern of C2H2 zinc finger (ZF) domains. Each group has further been divided into subsets based on the variation in C2H2 domain sequences. Numbers of SrC2H2-ZFPs in the 10 different subsets containing 1–4 non-tandem C2H2-ZF domains and number of proteins with up to three (t1) or more than three (t2) C2H2-ZF domains with at least two tandemly repeated domains are presented. Detailed information on corresponding genes is available in Supplementary Dataset S1.

**Figure 2:**
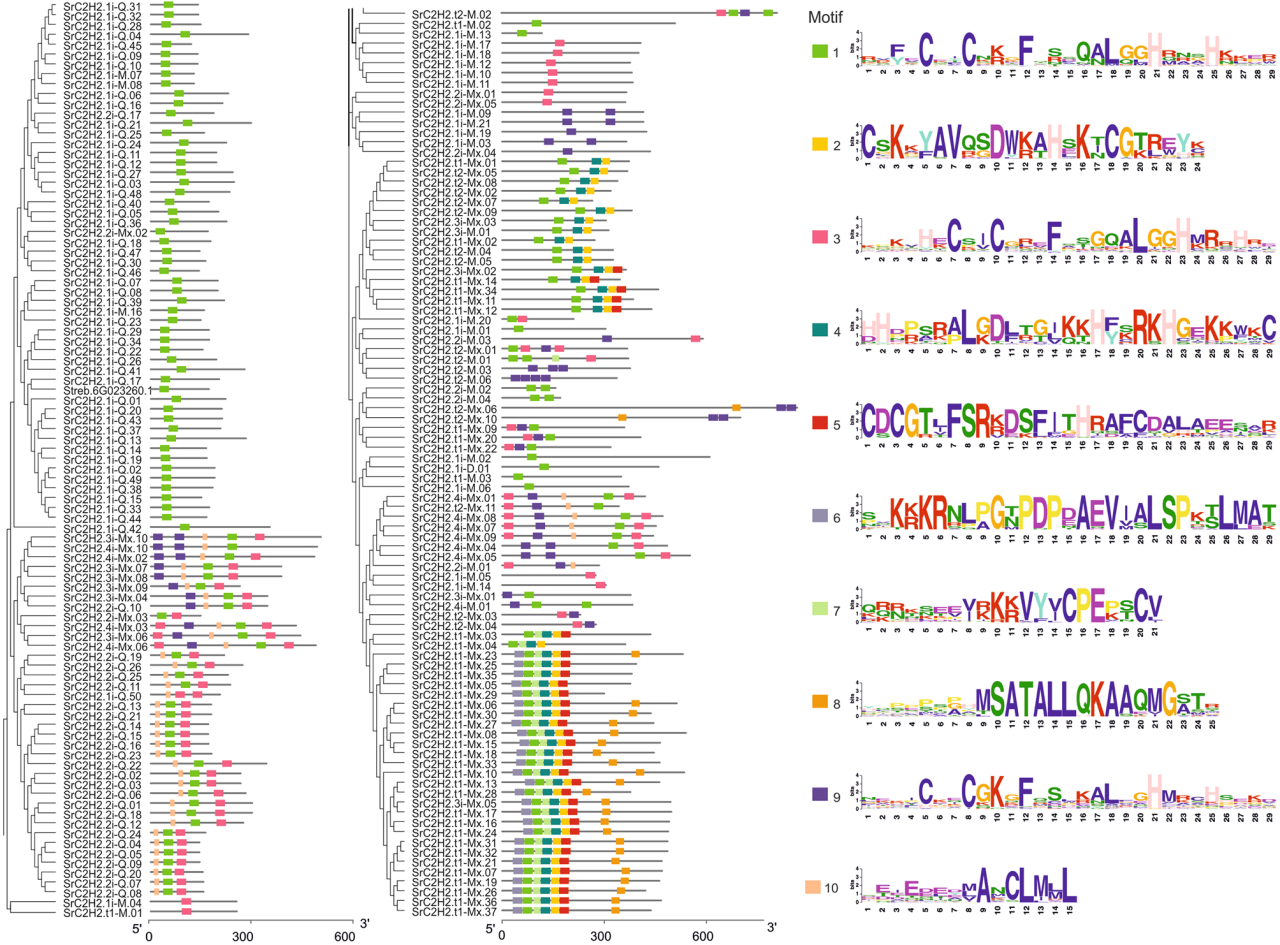
Conserved motifs of SrC2H2-ZFPs in *S. rebaudiana*. Ten conserved motifs determined using MEME are shown from which motifs 1, 3, and 9 are C2H2 zinc finger.

We generally classified the stevia SrC2H2-ZF proteins into non-tandem and tandem groups. Each of these two groups was further divided into subgroups based on the number and pattern of ZFs and the conservation rate of the QALGGH domain (Fig. 1). For example, 1i subgroup contains a single C2H2-ZFPs domain (e.g. 1i-Q, 1i-M), 2i harbours two C2H2-ZF domains (e.g. 2i-Q and 2i-M), and ni-Mx contains at least two types of n non-tandemly repeated C2H2-ZF domains. C2H2-ZFPs containing 3 or 4 non-tandem ZF domains were defined as 3i and 4i, respectively. Proteins with three or more than three ZF domains with at least two tandemly repeated ZF domains were presented as t1 and t2, respectively. For instance, t1-M has three M-type ZF domains and t2-Mx has at least four ZF domains composed of at least two different ZF types (Supplementary Dataset S1). Conserved motifs of SrC2H2-ZF proteins in *S. rebaudiana* were also identified by MEME. Ten conserved motifs were determined as shown in Fig. 2 from which motifs 1, 3, and 9 are C2H2 zinc finger proteins.

### Additional domains outside of the C2H2-ZF domains

To improve the classification of the 185 *S. rebaudiana* C2H2-ZFPs, the complete protein sequence of each SrC2H2-ZFP was examined using the SMART tool to identify any other domains besides the C2H2-ZF domains. In total, 14 other known domains outside the C2H2-ZF domains were recognized on 21 different SrC2H2-ZF proteins (Supplementary Dataset S1). These domains include the transmembrane domain, Zinc ion binding (ZnF_U1, ZnF_AN1, ZnF_Rad18), Histone binding/phosphorylation/methylation domains (PreSET, SET, PostSET, JmjN, JmjC), coiled coil, EXOIII, DnaJ, JmjN, JmjC, PUG, UBX. The first group consists of 164 proteins which solely harbored C2H2-ZF domains, without any other additional detected domains. Seven SrC2H2-ZF proteins contained coiled-coil domain. Each of ZnF_U1 and EXOIII were present in three different SrC2H2-ZFPs. Each of transmembrane and ZnF_AN1 were present in two SrC2H2-ZF proteins and 4 other SrC2H2-ZFPs proteins contained a combination of two or three other domains. Based on the subcellular localization predictions, 179 (97%) members of *S. rebaudiana* C2H2-ZFPs are localized in the nucleus, 5 (3%) C2H2-ZFs are localized in the chloroplast and nucleus, and 1 is localized in Golgi apparatus and nucleus (Supplementary Dataset S1).

### Phylogeny of the Stevia C2H2-ZF proteins

**Figure 3:**
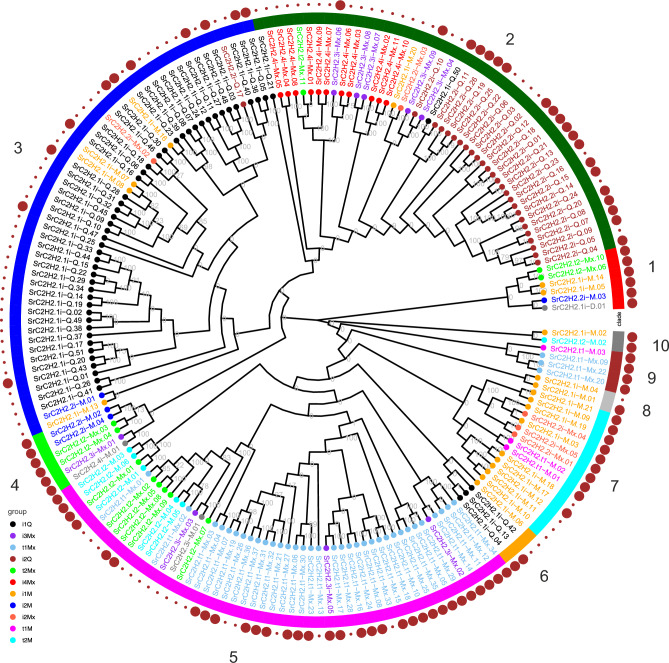
Phylogenetic classification of *S. rebaudiana* C2H2-ZF proteins. The proteins were aligned by Clustal-omega and the phylogenetic tree was determined by using the ape package in R with the Neighbor–Joining (NJ) algorithm and a bootstrap analysis of 1,000 replicates. The tree represents 10 *S. rebaudiana* C2H2-ZFP clades as shown by numbers 1 to 10 outside the circle. The color of the protein names is based on C2H2-ZFP types. Brown circles outside of the tree indicate the mean expression of the corresponding genes based on RNAseq analysis of seven different tissues^31^, where small and big circles indicate means less or more than 1 FPKM, respectively.

We assessed the phylogenetic relationships of the stevia SrC2H2-ZF proteins. The result is presented in Fig. 3, and the detailed gene IDs is available in Supplementary Dataset S1. The phylogenetic tree shows that the bootstrap supports for some main clades are considerably low, however, most of the SrC2H2-ZFPs in the same subgroup were clustered together with a high bootstrap support indicating that a high structural variation exists among different types of SrC2H2-ZFPs. Generally, the peptide sequences were relatively conserved among different members of SrC2H2-ZFPs of each subgroup, suggesting that the phylogenetic classification led to a reasonable tree.

### Structural properties and evolutionary divergence of the *SrC2H2-ZFP* gene family

Gene structure analysis (Supplementary Figure S2) revealed that out of the 185 identified C2H2-ZFP genes, 92 (50%) lack intron. The remaining genes contained 1 to 11 introns with length ranging from 31 (in Streb.Contig00126G000030) to 19,496 (in Streb.1G026630) bases. Only three genes had intron longer than 9000 bases (Supplementary Dataset S3). No correlation between the number of C2H2-ZF domains and the number of exons in corresponding gene (Supplementary Dataset S1) was observed for stevia C2H2-ZFPs (R^2^ = 9.0%). We analyzed the evolutionary time divergence between coding sequences of 15 most recently duplicated pairs of the SrC2H2-ZFP genes (Supplementary Dataset S4). The result revealed that these genes have been duplicated and diverged 0.08–14.2 million years ago (MYA). However, for most (73%, 11 out of 15) of the analyzed genes, the divergent time ranged from 0.08 to 2.9 MYA, suggesting a recent duplication event for *SrC2H2-ZFP* genes. All the studied gene pairs had a Ka/Ks value less than 1, indicating that they have been subjected to purifying selection.

### Analysis of chromosomal locations of *SrC2H2-ZFP* genes

**Figure 4:**
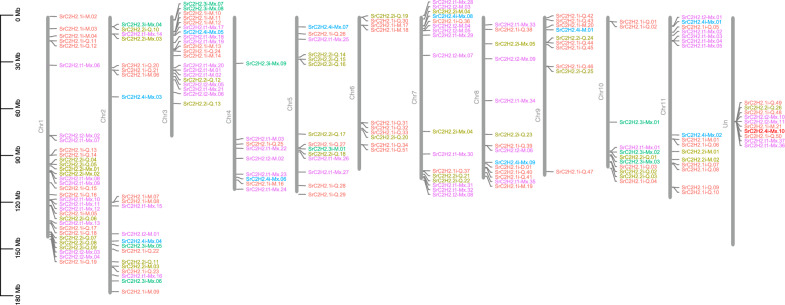
Physical map of *C2H2-ZFP* genes on the *Stevia rebaudiana* chromosomes. The density of *C2H2-ZFPs* along the chromosomes is highly accompanied by general gene density in the *S. rebaudiana* genome^31^. The genes are colored according to the Fig. 1 classification. Ten *C2H2-ZFP* genes were located on unassembled contigs and were not presented on this map.

A physical map of the location of the *SrC2H2-ZFP* genes on the *S. rebaudiana* chromosomes is illustrated in Fig. 4. The *SrC2H2-ZFP* genes were mapped to 11 stevia chromosomes plus the unassembled part of the genome. The *SrC2H2-ZFP* genes showed uneven distribution across the chromosomes with an average of 16 genes on each chromosome and a higher density on chromosome 1 which harbored 32 genes. *SrC2H2-ZFP* genes were mainly localized toward the distal ends of the chromosome arms. The genes with similar ZF domain architecture are mostly clustered together on the chromosomes.

### Cis-regulatory elements and miRNAs

**Figure 5:**
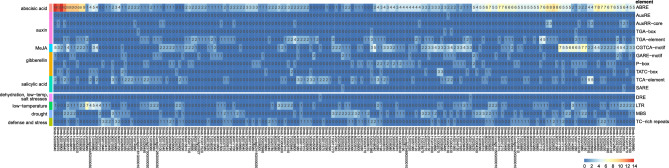
Heatmap of possible cis-acting elements in the 2-kb upstream of the *SrC2H2-ZFP* genes of *S. rebaudiana*. The elements were predicted using the PlantCARE website^37^. The number of the identified elements (shown on the right) for each gene is presented inside the cells and the stimuli are indicated on the left.

To illuminate how *S. rebaudiana SrC2H2-ZFP* genes respond to external cues, the 2 kb promoter region of 185 *SrC2H2-ZFP* genes was analyzed using the PlantCARE database. In total, 5319 potential regulatory elements possibly responding with light, hormonal signals, defense and stress-related, etc. (Fig. 5 and Table 1) were identified. Considering only the factor presented in Table 1, each *SrC2H2-ZFP* gene promoter harbored 2 (for Streb.2G014040 promoter) to 61 (for Streb.2G005970 promoter) cis-acting elements. While light-responsive elements predominated, binding sites for hormonal, drought, and low-temperature signals were also identified. Although cis-elements responding to light was most widely distributed, we only presented the number of identified cis-acting elements related to hormone and environmental stresses in Fig. 5. Streb.5G008640 and Streb.2G005860 had the most abundant cis-acting elements related to hormone (22) and low-temperature (7). The maximum drought related element (MBS binding element) was 3 which was observed for the promoters of Streb.9G010930, Streb.11G011770, Streb.11G018970, and Streb.7G004510 genes (Table 1). A considerable number of cis-acting elements related to abscisic acid (ABA) compared to other stimuli was observed in SrC2H2-ZFP promoter regions (Fig. 5). Different types of promoter cis-components were observed, such as ABRE (abscisic acid response element), AuxRR and TGA (auxin response element), CGTCA (methyl jasmonate response element), GARE, P-box and TATC-Box (gibberellin response element), TCA and SARE (salicylic acid response element), DRE (dehydration, low temperature and salt stress responsive element), MBS (drought response element), LTR (low temperature response element), and TC rich repeats that may be involved in the defense and stress response.

**Table 1:** The identified cis-regulatory elements related to different stimuli on the 2 kb upstream of *SrC2H2-ZFP* genes in *S. rebaudiana*.

Stimulus	Number of elements
Light	2465
MeJA	689
Abscisic acid	610
Anaerobic induction	438
Auxin	132
Gibberellin	131
Low-temperature	123
Drought	113
Salicylic acid	112
Zein metabolism	104
Defense and stress	84
Meristem expression	78
Endosperm expression	47
Circadian control	41
60K protein binding site	28
Flavonoid biosynthesis	23
Seed-specific regulation	18
Cell cycle	16
Protein binding site	16
Wound	9

We found miRNA targets in intronic sequences for five genes including Streb.1G035890 (ath-miR5658), Streb.2G029620 (pta-miR171), Streb.11G004260 (bdi-miR845), Streb.6G002350 (gma-miR5035-3p) and Streb.5G019230 (ath-miR5658), all at the expectation value of 1.5 for Streb.1G035890 which was 1. At the cDNA level, 6 SrC2H2-ZFP genes were the target for ath-miR5658 and one was the target for osa-miR2055 with cut-off expectation threshold of 0, 1 or 1.5 (Supplementary Dataset S3).

### SrC2H2-ZFPs expression patterns

**Figure 6:**
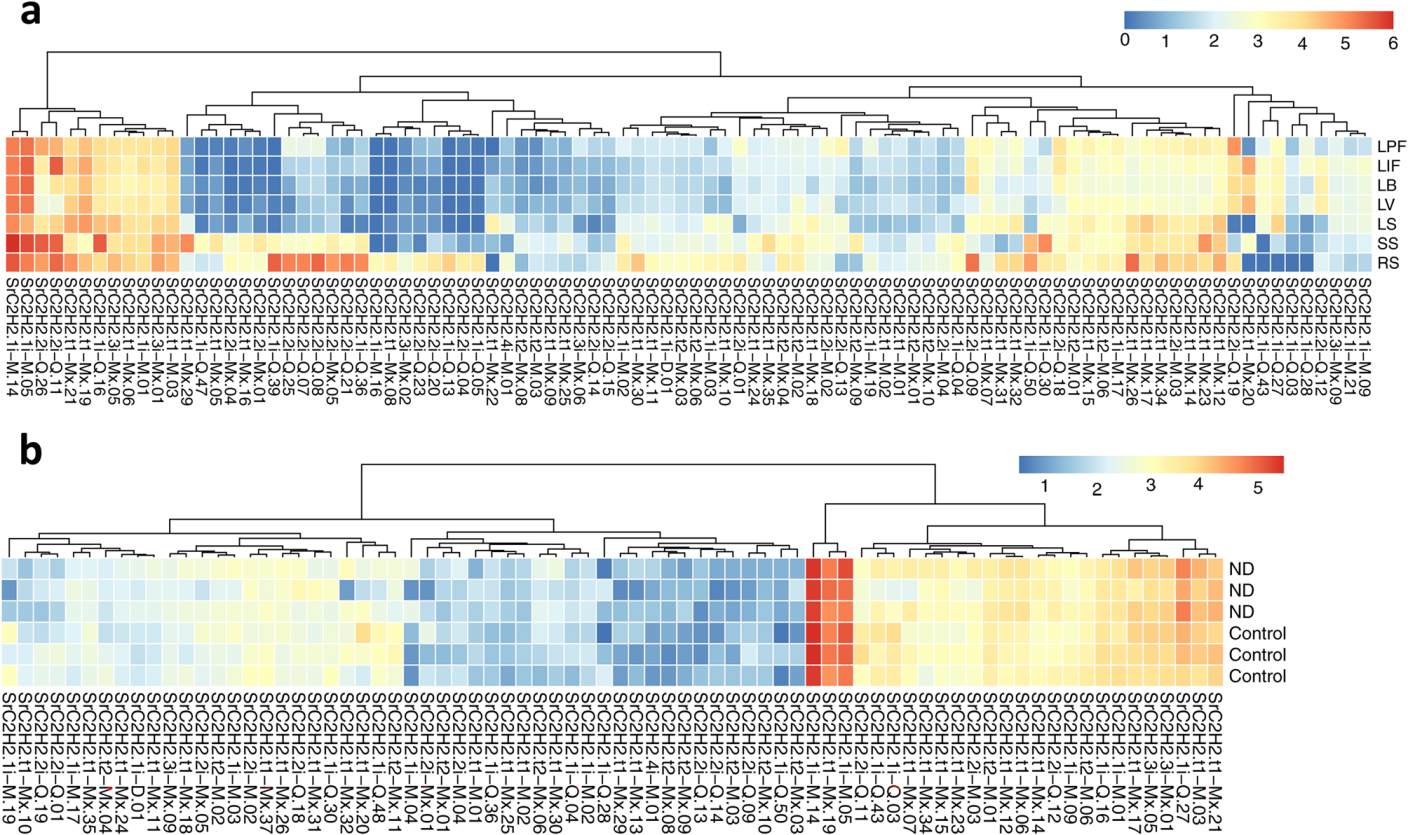
SrC2H2-ZFP gene expression patterns. **a**) Expression patterns of SrC2H2-ZFP genes in different tissues of *S. rebaudiana*. RS, SS, and LS: root, stem and leaf at the seedling stage, respectively. LV, LB, LIF, and LPF: leaf at vegetative, bud, initial flowering and peak flowering stages, respectively. **b**) Expression patterns of C2H2-ZFP genes in stevia leaves under Control and nitrogen deficiency (ND). The expression rates are based on Log2(FPKM + 1) and C2H2-ZFP genes with a mean expression of less than 1 FPKM were excluded.

To get insight into the SrC2H2-ZFPs expression patterns, we used two independent set of publicly available RNAseq reads, one from seven different developmental stages of *S. rebaudiana* including root at seedling stage (RS), stem at seedling stage (SS) leaves at seedling stage (LS), leaf at vegetative (LV), leaf at bud (LB), leaf at initial flowering (LIF) and leaf at peak flowering stage (LPF)^31^ and the other set from leaves under control and nitrogen deficiency^18^. From 185 identified C2H2-ZF proteins, 91 and 109 had a mean expression of less than 1 FPKM (fragments per kilobase of transcript per million fragments mapped) in different developmental stages and under nitrogen deficiency conditions, respectively. These low transcriptionally active genes were discarded from the expression analysis. The expression heatmap of the remaining genes generated from RNAseq data of developmental stages (Fig. 6A) indicates that two groups of *C2H2-ZFP* genes exist based on their expression patterns. Group 1 genes are highly expressed in all *S. rebaudiana* tissues while group 2 genes are expressed specifically at seedling or flowering stages. Among the latter, some genes expressed specifically at the RS (root at seedling) stage and switched off in aerial parts of *S. rebaudiana* while a few genes (e.g. *SrC2H2.1i-Q.43*) showed a reverse expression pattern. Some genes such as *SrC2H2.t1-M.03* (*Streb.4G011560*) expressed evenly in all stages. Some genes such as *SrC2H2.2i-Q.26* and *SrC2H2.2i-Q.11* showed extremely high expression at the RS (root at seedling) stage. We further intended to analyze RNAseq data from experiments conducted under various stress treatments to elucidate the expression patterns on *C2H2-ZFP* genes under stress conditions as well. Based on the RNAseq data from the nitrogen deficiency investigation, none of the members of the C2H2-ZFP family was significantly affected by nitrogen deficiency in the leaves of stevia under nitrogen-deficient conditions (Fig. 6B), although three members of the C3H-ZF family were among the upregulated genes^18^.

**Figure 7:**
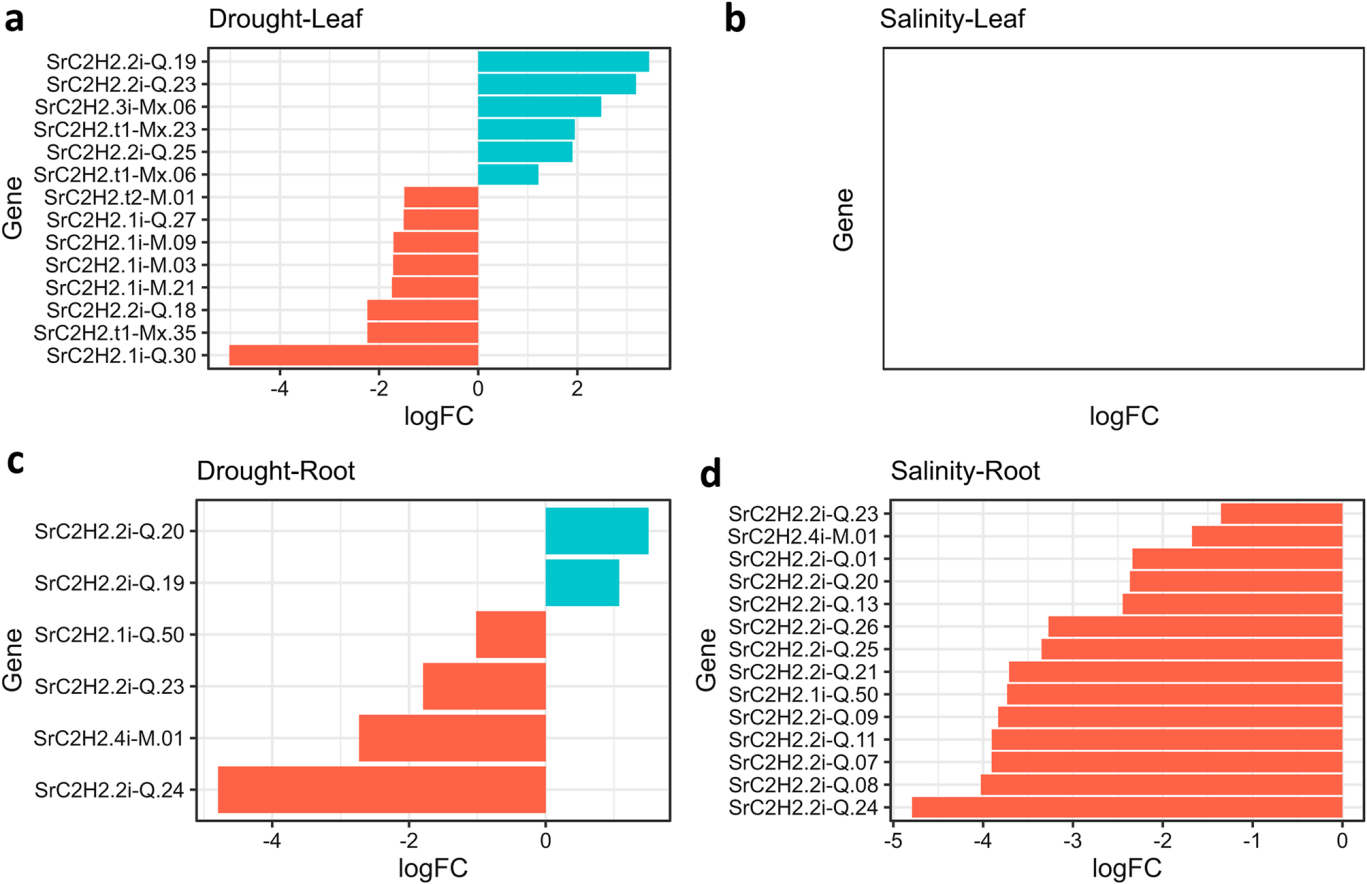
Barplots analysis of differentially expressed *SrC2H2-ZFP* genes (FDR < 0.05) in leaves (**a** and **b**) and roots (**c** and **d**) of *S. rebaudiana* under drought and salinity stresses based on published RNAseq data^6^. Up- and down-regulation are indicated by cyan and orange colors, respectively. Under salinity conditions, no significantly up- or downregulated C2H2 genes were found for leaf (**c**) but many of them have been down-regulated in root (**b**).

**Figure 8:**
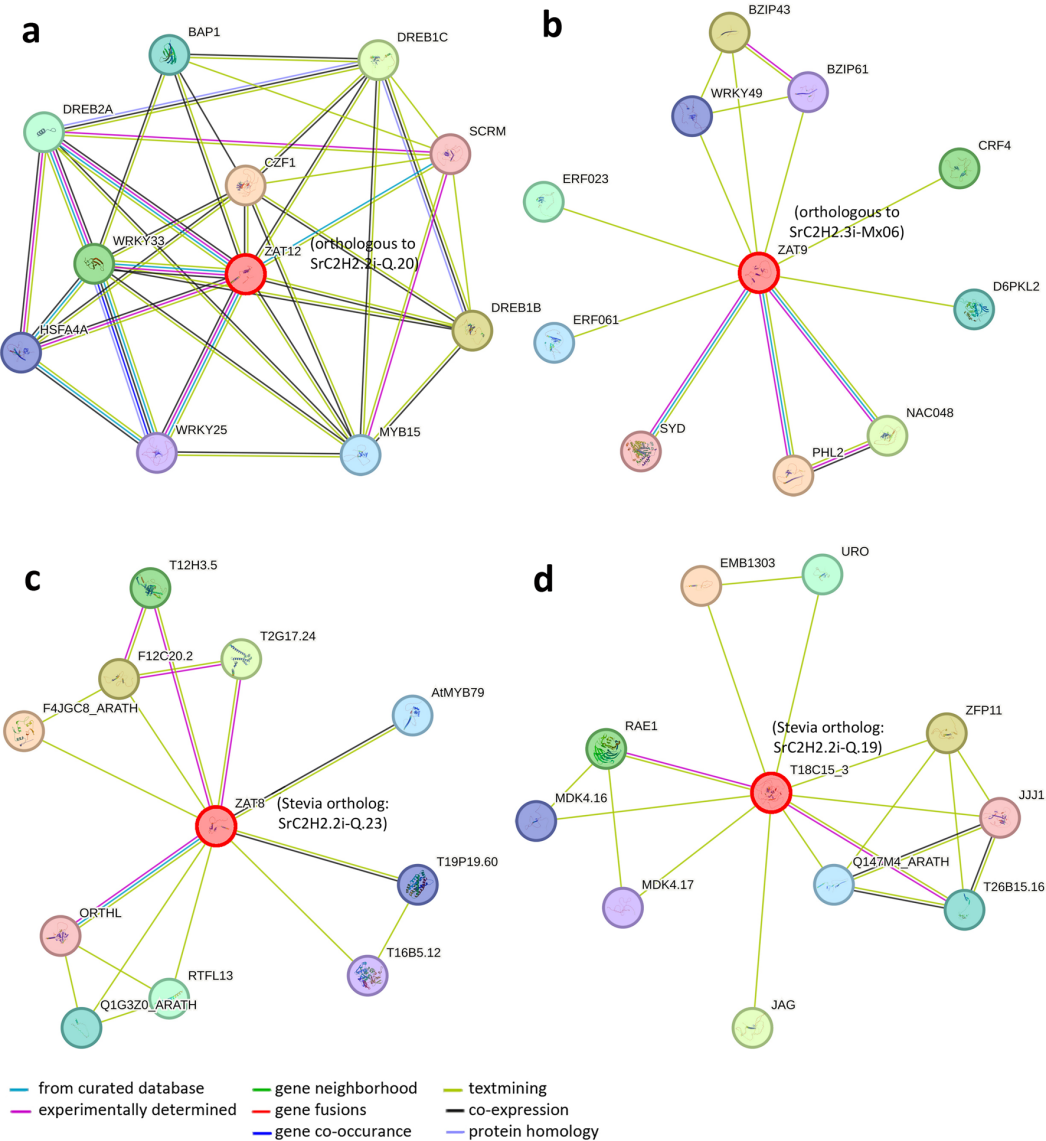
The protein–protein interaction networks for the Arabidopsis orthologs of four significantly overexpressed stevia genes under drought stress: (**a**) ZAT12 or RHL41 (AT5G59820) which is orthologous to SrC2H2.2i-Q.20; (**b**) ZAT9 (AT3G60580) is orthologous to: SrC2H2.3i-Mx.06; (**c**) ZAT8 (AT3G46080) which is orthologous to SrC2H2.2i-Q.23; (**d**) T18C15_3 (AT1G49900) is orthologous to SrC2H2.2i-Q.19. Nodes are proteins, and edges are interactions, colored by evidence type.

We further investigated the expression changes of *SrC2H2-ZFP* genes under drought and salinity conditions from the RNAseq results of a recently published study^6^. From the 185 SrC2H2-ZFP genes, 25 were up- or down-regulated in stevia leaves or roots by drought or salt stress (Fig. 7, Supplementary Dataset S5). Of these, six SrC2H2-ZFP genes were up- and eight were down-regulated in stevia lives followed by drought stress. No significantly overexpressed SrC2H2-ZFP gene was detected in leaves or roots under salinity conditions, although 14 were down-regulated in roots. Arabidopsis orthologs of the stevia SrC2H2-ZFP genes which were differentially expressed under drought or salt stresses were determined using BlastP (Supplementary Dataset S6) and protein interaction networks for the Arabidopsis orthologs of four significantly overexpressed stevia genes under drought was constructed using STRING database (Fig. 8). The corresponding proteins are ZAT12, ZAT9, ZAT8 and T18C15_3. ZAT12 or RHL41 (AT5G59820) is orthologous to *SrC2H2.2i-Q.20* (Fig. 8A), ZAT9 (AT3G60580) is orthologous to *SrC2H2.3i-Mx.06* (Fig. 8B), ZAT8 (AT3G46080) is orthologous to *SrC2H2.2i-Q.23* (Fig. 8C) and T18C15_3 (AT1G49900) is orthologous to *SrC2H2.2i-Q.19* (Fig. 8D). The predicted functional partners for the above network clusters are presented in Table 2, showing the score and number of nodes and edges in each cluster, and, localization of the partner proteins. Most of the partner proteins were transcription factors and predicted to be localized in nucleus, although a few proteins showed cytoplasm, endoplasmic reticulum (ER) or chloroplast localization (Table 2).

**Table 2:** STRING database details about the protein–protein interaction networks predicted in Fig. 8.

Cluster and cluster input	PPI enrichment P-value	Nodes	Edges	Predicted functional partners (Score)
A (ZAT12*)	0.000	11	37	CZF1 + ^ (0.873), DREB1B* (0.87), DREB1C* (0.865), WRKY33* (0.847), DREB2A* (0.844), BAP1* (0.844), MYB15* (0.843), HSFA4A* (0.818), WRKY25* (0.81), SCRM* (0.798)
B (ZAT9*)	0.144	11	14	PHL2* (0.684), BZIP43* (0.679), NAC048* (0.643), CRF4* (0.599), ERF023# (0.583), D6PKL2* (0.54), ERF061* (0.54), WRKY49* (0.54), BZIP61* (0.518), SYD* (0.506)
C (ZAT8*)	0.028	11	17	F4JGC8_ARATH* (0.796), F12C20.2* (0.79), T2G17.24* (0.786), T12H3.5 + (0.786), RTFL13* (0.784), Q1G3Z0_ARATH# (0.695), AtMYB79* (0.645), T19P19.60 + (0.643), T16B5.12* (0.631), ORTHL + * (0.619)
D (T18C15_3*)	0.009	11	19	EMB1303# (0.784), ZFP11* (0.716), JAG* (0.597), RAE1 + (0.558), URO* (0.547), T26B15.16* (0.544), Q147M4_ARATH* (0.544), MDK4.16* (0.544), MDK4.17* (0.542), JJJ1* (0.541)

Protein localization: *Nucleus; + :cytoplasm; ^Endoplasmic reticulum (ER); #Chloroplast.

### Relative expression of SrC2H2-ZFP genes under drought stress

To get insight into the expression of SrC2H2-ZFP genes, three C2H2 genes (including *SrC2H2.t1-Mx.26*, *SrC2H2.1i-M.01*, and *SrC2H2.3i-Mx.01*), were selected for expression analysis. Supplementary Dataset S7 details the sqRT-PCR primers and their expected amplicon sizes. SqRT-PCR was performed with the cDNA samples prepared from leaves total RNA. Although low-level variations were observed, none of the analyzed genes showed significant expression levels between the control and drought-stressed conditions as revealed by statistical comparisons after quantifying the RT-PCR band intensities (Supplementary Figure S3).

## Discussion

### Classification of the SrC2H2-ZF proteins

The C2H2-ZFP gene family is one of the well-studied gene families and has been studied in many plants such as Arabidopsis^41^, petunia^42^, wheat^43,44^, cotton^45,46^, soybeans^15^, rice^47^ and sorghum^17^. These proteins are well-known for their involvement in various developmental processes^48,49^ as well as in response to abiotic stresses such as drought and salinity^10,11, 50–52^. We identified a total of 185 putative SrC2H2-ZF proteins from the genome sequence of *S. rebaudiana* that in total encode 283 transcripts. All the SrC2H2-ZF proteins were predicted to localize in the nucleus supporting their role as a transcription factor in the nucleus. 5 C2H2-ZF proteins were also predicted to be localized in chloroplast suggesting that they may be also involved in chloroplast gene expression.

We followed a generally accepted method for the classification of stevia C2H2-ZF proteins based on the number and patterns (tandem or dispersed) of zinc fingers distribution^41^, and the plant-specific QALGGH conserved motif^13^. Based on this method, the C2H2-ZFP family in stevia is divided into 5 main groups and 10 subgroups (Fig. 1 and Supplementary Dataset S1). Alternatively, the C2H2-ZFP gene family in Arabidopsis has been divided into A, B, and C groups where A1 (containing tandemly arranged ZFs) and C1 (one or two to five dispersed ZFs and with a QALGGH motif in the ZF domain) compose the two largest and evolutionarily youngest and active C2H2-ZFPs^41^.

Most of the stevia C2H2-ZF proteins contain one or more C2H2 zinc fingers with the QALGGH motif and were classified as Q-type. The Q-type C2H2-ZFPs are plant-specific and their abundance suggests their involvement in plant developmental and physiological processes as well as responses to biotic and abiotic stresses^11–13,53^.

### Phylogeny, physical distribution, and gene duplication

Phylogeny, physical mapping and domain and motif analysis of the stevia C2H2-ZF proteins indicate that the genes with similar ZF domain architecture are mostly clustered together in the genome suggesting tandem gene duplication and possibly their similar biological functions. Gene duplication is a major drive of evolving the paralog members within gene families^54^, commonly undergoing new functionalization or pseudogenizations^55–57^.

Analysis of the evolutionary time divergence between coding sequences of 15 most recently duplicated pairs of the SrC2H2-ZFP genes (Supplementary Dataset S3) revealed a duplication time divergence of 0.08–14.2 MYA. However, for most (11 out of 15, 73%) of the analyzed gene pairs, the divergent time ranged from 0.08 to 2.9 MYA, suggesting a recent duplication event for *SrC2H2-ZFP* genes. All the studied gene pairs had a Ka/Ks value less than 1, indicating that they have been subjected to purifying selection, thus, they are conserved and expected to retain conserved functionality, while pairs with a high divergence time value may acquire new functions^58^.

### Evolution of *SrC2H2-ZFP* gene family

Cis-regulatory elements are non-coding regions of DNA that govern the transcription of nearby genes. Cis elements including enhances and silencers are present in variable distances from the gene and can be several kilobase away from the gene^59^. A reasonably higher number of cis-acting elements for abscisic acid compared to other stimuli was observed in SrC2H2-ZFP promoter regions emphasizing a general involvement in abscisic acid pathways related to abiotic stress responses. In fact, C2H2-ZF proteins can induce abiotic stress tolerance through ABA-mediated signal pathways^10,11^.

### *SrC2H2-ZFP* gene expression

The analysis of the publicly available RNAseq data revealed that the stevia *C2H2-ZFP* genes are interactively expressed in different tissues and developmental stages. C2H2-type zinc finger proteins play widespread functions in plant tolerance to different abiotic stresses, such as drought, salinity, cold, osmotic and oxidative stresses^10,11^. Genes of the C2H2 family have shown differential expression under salt and drought stresses in *Brassica rapa*^16^. Responsive C2H2 genes were also identified under cold and drought in *Sorghum bicolor*^17^ and Chinese cabbage^60^, and under salt and drought stresses in *Solanum tuberosum*^61^ and *Larix kaempferi*^62^.

We also looked for the expression changes of *SrC2H2-ZFP* genes under drought and salinity stresses^6^. Out of the 185 SrC2H2-ZFP genes, 25 genes (~ 13%) were up- or down-regulated in stevia leaves or roots by drought or salt stress. The analysis of the Arabidopsis orthologous of these genes (Supplementary Dataset S5) showed that they are involved in abiotic stress tolerances possibly via gibberellin (*GAF1-2*, *AT3G50700*)^63^ and abscisic acid signaling^10^. *SrC2H2.2i-Q.23* is one of the genes up-regulated in leaves (Fig. 7A) but down-regulated in roots under drought stress (Fig. 7C). It has been reported that overexpression of the Arabidopsis ortholog of *SrC2H2.2i-Q.23* (i.e. *ZAT18*, *AT3G53600*) improves drought tolerance as revealed by higher leaf water content and antioxidant enzyme activities, but lower content of reactive oxygen species after drought treatment compared to control plants^64^. ZAT18 also is connected with RAS1 (Fig. 8A) which is a negative regulator of salt tolerance probably by enhancing abscisic acid sensitivity^65^.

We further analyzed the expression of *SrC2H2.t1-Mx.26*, *SrC2H2.1i-M.01*, and *SrC2H2.3i-Mx.01* genes by sqRT-PCR. The sqRT-PCR reactions were performed with the cDNA samples belonging to the total RNA isolated from the leaves. Although low-level variations were observed, the expression of none of the analyzed genes significantly changed by the drought stress (Supplementary Figure S2). So, the analyzed genes were not among those that are affected by drought. We selected these genes because they showed expression in leaves based on the results of the analysis of publicly available RNAseq data for different tissues and developmental stages which was not under any stress condition^31^.

### Cis-regulatory elements and miRNA targets

The analysis of cis-acting elements in SrC2H2-ZFP promoter regions suggests that the stevia C2H2-ZFP family members generally responds to light and phytohormones indicating that they could play a role in abiotic stresses via hormone responses such as abscisic acid (ABA), methyl jasmonate (MeJA), salicylic acid (SA), and auxin. Some SrC2H2-ZFP genes harbours considerable number of ABA responsive elements and some genes contain both ABA and drought response cis-elements in their promoter regions suggesting that they may respond to drought stress through the ABA signaling pathway^66^. At the maximum expectation value of 1.5, we found miRNA targets in intronic sequences for five genes and at the cDNA level, 5 SrC2H2-ZFP genes were the target for ath-miR5658 (Supplementary Dataset S3). In Arabidopsis, miR5658 is mainly seen in the nucleus and activate the expression of AT3G25290 (an auxin-responsive family protein) directly by binding to its promoter^67^. Hence, possibly there are other regulation layers for SrC2H2-ZFP genes involving auxin pathway and miR5658 miRNA.

## Conclusion

A comprehensive genome-wide analysis of C2H2-ZFPs in *S. rebaudiana* led to the identification of 185 SrC2H2-ZFP genes distributed over all the chromosomes. SrC2H2-ZF proteins were grouped into 5 groups and 12 subgroups based on the number and pattern of C2H2 zinc finger domains. All the SrC2H2-ZF proteins were predicted to localize in the nucleus. The Q-type C2H2-ZFPs were the most abundant C2H2-ZF proteins. As supported by physical mapping and phylogeny, we argued that gene duplication events have significantly expanded and evolved the stevia C2H2-ZFP genes. The SrC2H2-ZFP Expression patterns in different tissues and developmental stages as well as under drought and salinity conditions confirmed that Q-type C2H2-ZFPs along with the M and Mx types are interactively involved in development and drought stress responses. Some genes such as *SrC2H2.2i-Q.19* were upregulated in both roots and leaves under drought condition. The study identified some target gene for further genetic improvement of stevia. The protein–protein interaction networks can further clarify the potential functions of C2H2-ZF proteins. In conclusion, our results provide a root for in-detailed characterization of C2H2-ZFP functions in growth and development and abiotic stress response in stevia.

### Supplementary Information

Supplementary Information 1.

Supplementary Information 2.

Supplementary Information 3.

Supplementary Information 4.

Supplementary Information 5.

Supplementary Information 6.

Supplementary Information 7.

Supplementary Figure S1.

Supplementary Figure S2.

Supplementary Information 10.

Supplementary Information 11.

Supplementary Figure S3.

## Data Availability

All data generated or analyzed during this study are included in this published article and its supplementary information files.
