# Supplementary material for: Genome-wide analysis of the C2H2-ZFP gene family in Stevia rebaudiana reveals involvement in abiotic stress response
Source: Sci Rep. 2024 Mar 14;14:6164. doi: 10.1038/s41598-024-56624-y (PMC10940304; doi:10.1038/s41598-024-56624-y)
Supplement: Supplementary file 9 — Supplementary Figure S2. [file 41598_2024_56624_MOESM9_ESM.pdf]

Genome-wide analysis of the C2H2 -ZFP gene family in *Stevia rebaudiana* reveals involvement in abiotic stress response

Shahla Nikraftar, Rahman Ebrahimzadegan, Mohammad Majdi, Ghader Mirzaghaderi

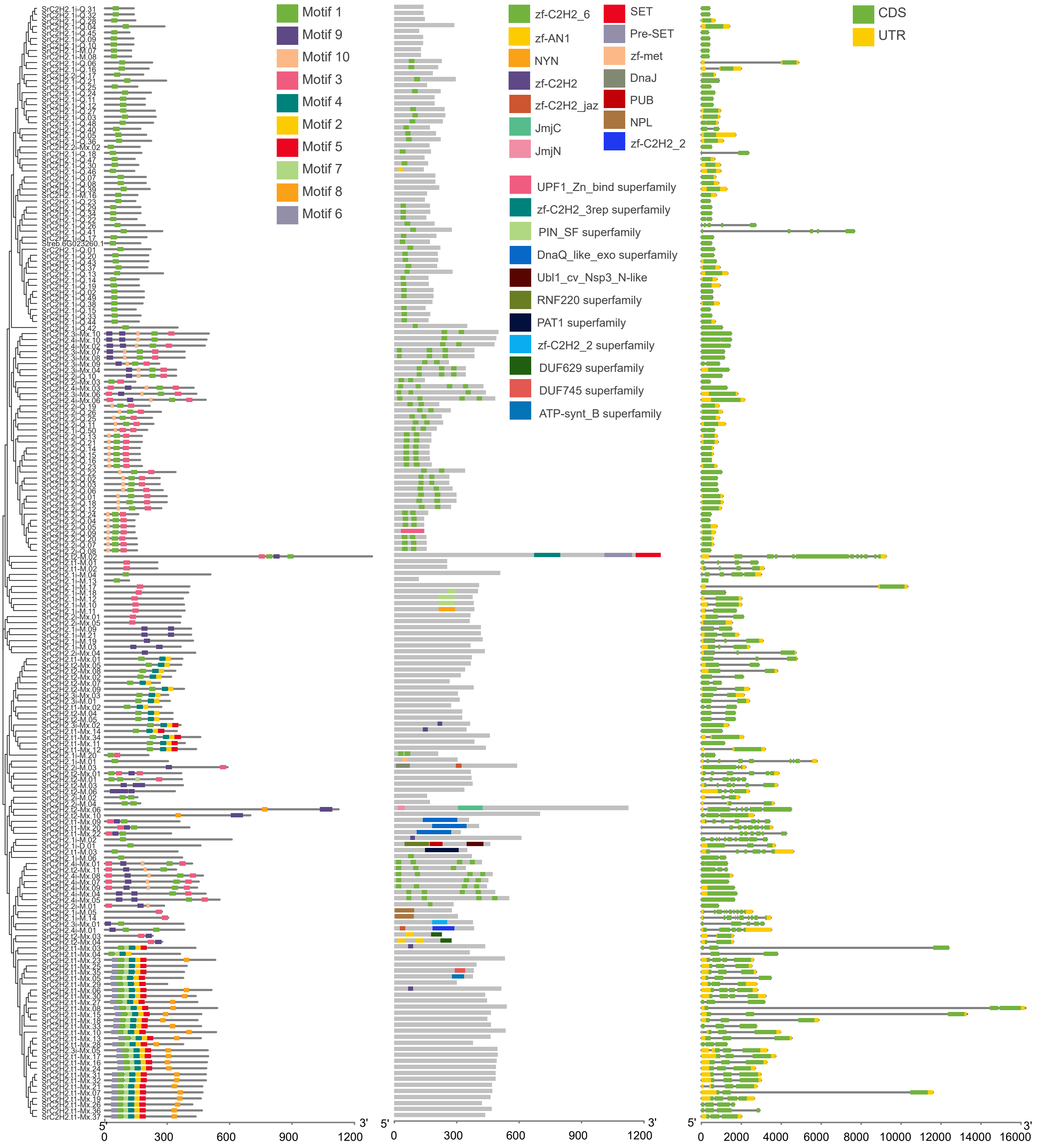

Supplementary Figure S2. Motif, domain and gene structure of the stevia SrC2H2-ZFPs.
