# Supplementary material for: Genome-wide analysis of the C2H2-ZFP gene family in Stevia rebaudiana reveals involvement in abiotic stress response
Source: Sci Rep. 2024 Mar 14;14:6164. doi: 10.1038/s41598-024-56624-y (PMC10940304; doi:10.1038/s41598-024-56624-y)
Supplement: Supplementary file 10 — Supplementary Information 10. [file 41598_2024_56624_MOESM10_ESM.pdf]

Genome-wide analysis of the C2H2-ZFP gene family in *Stevia rebaudiana* reveals involvement in abiotic stress response

Shahla Nikraftar, Rahman Ebrahimzadegan, Mohammad Majdi, Ghader Mirzaghaderi

a

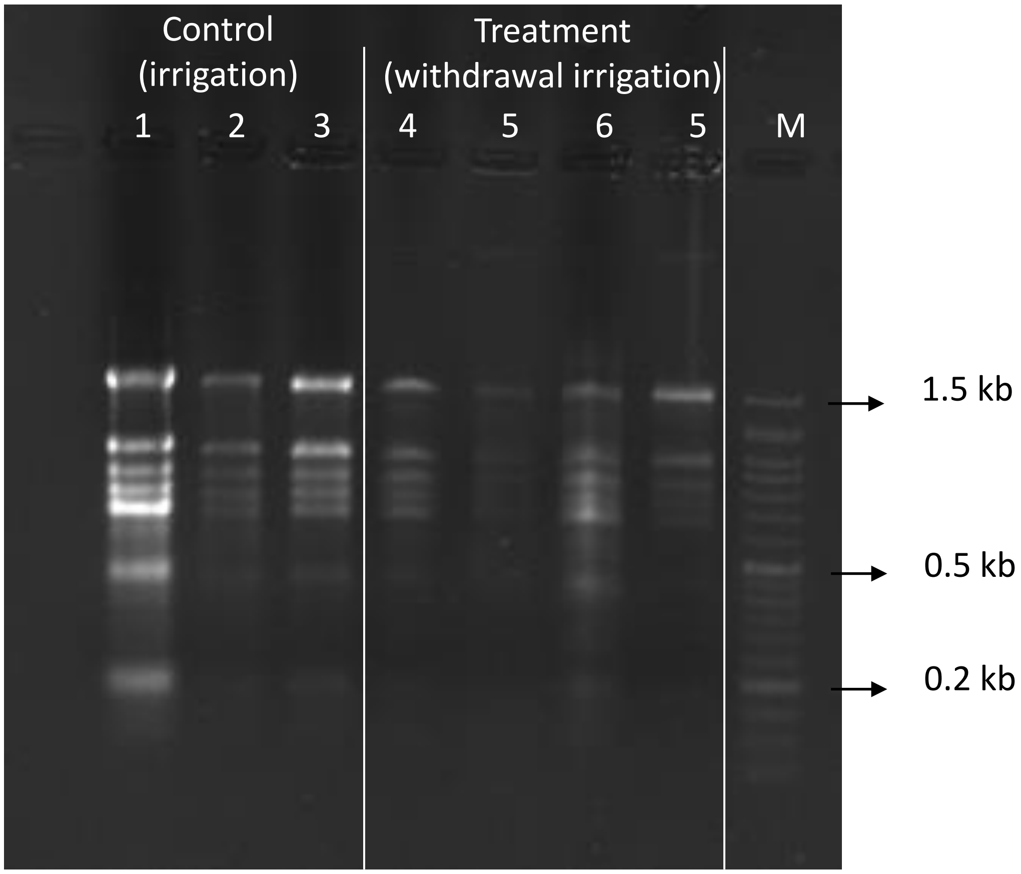

b

	RNA Samples	Concentration (ng/µl)	260/280	260/230
Control	1	703	1.87	2.11
	2	239	1.73	1.94
	3	480	1.79	2.07
Treatment	4	230	1.69	1.84
	5	189	1.52	1.79
	6	245	1.71	1.88
	5	243	1.81	1.91

Supplementary File S1. a) Total RNA extracted from the leaves of control (irrigated) and treatment (withdrawal irrigation) plants. **b)** The Concentration and the ratio of absorbances at 260 nm and 280 nm for each sample.
