# Supplementary material for: Genome-wide analysis of the C2H2-ZFP gene family in Stevia rebaudiana reveals involvement in abiotic stress response
Source: Sci Rep. 2024 Mar 14;14:6164. doi: 10.1038/s41598-024-56624-y (PMC10940304; doi:10.1038/s41598-024-56624-y)
Supplement: Supplementary file 11 — Supplementary Information 11. [file 41598_2024_56624_MOESM11_ESM.pdf]

Genome-wide analysis of the C2H2-ZFP gene family in *Stevia rebaudiana* reveals involvement in abiotic stress response

Shahla Nikraftar, Rahman Ebrahimzadegan, Mohammad Majdi, Ghader Mirzaghaderi

Supplementary File S2. SrC2H2-protein sequences of *Stevia rebaudiana*.

>SrC2H2.1i-Q.01
MAETGSLSNYNMNNKSMLKDHTSGLKSTNKAIITYGKSALECCREDHNNGEESFGEFSWPPKSYSCSFCKREFRSAQALGGHMN
VHRREKAKLRQVNPQRYLPFLYPQSQFLDLNLDQNPNPNSNLNPNPNTTNTSSFSTMFPSFTYSCCLPLSDPCSFHRLCLSNRA
RFEAFGSRKTDLVGDFTHERDGVIVKKKLETGLHVESEFDDLDLELRLGCS
>SrC2H2.1i-Q.02
MGSDQKHGDSETSSDQETSRPLSPNSSKDGLGRSYECTFCKRGFTNAQALGGHMNIHRKDKAKAKQHTTASAKLNKDHRLIITS
SKSFSHDPIEDSQYLGSSFAIRTSDDHGYQFIPSNPNFQWALAASQFDHNRALHEEHLRVNLNLGIGSSETENNINIESTKVGI
NEDWFENGVDLELRLGHYP
>SrC2H2.3i-Mx.01
MTGLSCNACNKELEDENDQKLHYKSEWHRYNLKRKIAGVPGVTEALFLARQSTLAEEKSKHNGPPMLYTCRLCGKGYRSAKAHA
QHLKSKTHTTRASQIGHEDDSNAIIKQLPPRVVKKSPQQKEEFDEESDESDEWVEVTENDDVAGDMASSSNPMEVNGDDDDSDD
DMDEDEDELDPTSCFMCDKEHKTIESCMVKRDYICLYCNSNCQPFNSLDAVRKHMVAKSHCKVHYGDDDEEEEAELEEFYDYSS
SYVDANGNQLVSSDGTSGGIELGSGGSELIITSVTDDTISTKAIGSREYLRYYRQKPRPSPDGVPITAVLAARYRSMGLSIVQS
KENMVRMKVMKQMNRSGVEHMRSKMGMKSNVIRNLPKNVTH
>SrC2H2.t1-Mx.01
MSSEYNPYYNKSCFNLNQQYLNPLSSSSMSSSSLYHYHELSPPSPHKEALPLLSLIPSKRVNHDHQNPNDSCAQFMEIDTNNNK
FSDDHETVTLHLGLPNHNLSEADLNSRFSNNNSHNDTEIDHKLEVVEEGSTNGNEYFTTTLNKGQYWIPTPSQILIGPTQFSCP
LCFKTFNRYNNMQYYVYSRGICLFCEFKSSKFGEPNLQYQKRTMWWFILQFSRKMHMWGHGSQYRRGPESLRGIQPTAMLKLPC
YCCAPRCHNNIDHPRAKPLKDFRTLQTHYKRKHGIKPFMCRKCSKPFAVRGDWRTHEKNCGKLWYCKCGSDFKHKRSLKDHIKA
FGNGHGACGVNVESCFEVEEEETANSEVDHQENESC
>SrC2H2.3i-Mx.02
MAVEPNYCFTNTYEDVLASSLQSTTNNNIVFNLSILKEKAQHVHSTAAVFFTQSQAQSVVVSNSTSVSLENIANLLQEIIAIAS
NVMFTCQNIVPGVNHSNPTNVDHDNTLEFDQLTQPNMMFEWPDGDGSDNRFGNVTYNARNKDSCLEKRKQFQVETIRQEERGSG
SRSTSKSGSGLRTTLGQNLSPKSYDIIELDAADLLAKYTHYCQVCGKGFKRDANLRMHMRAHGDEYKSVAALANPVSMNDPTST
KIARKYSCPQEGCRWNRKHAKFQPLKSMICMKNHYKRSHCPKMYSCKRCHQKQFSVLSDLRTHEKHCGDVKWRCSCGTTFSRKD
KLMGHVSLFIGHTPVIDTGSLINGHSN
>SrC2H2.2i-Q.01
MEMEFMAEFTEGMSRDRARQIIKRKRTKRQRPNSPSGDCLIDNFTSDQSPVMSSEISTEEDEDMANCLIMLARSVSPIKEVKSD
QIRQKTKRLSDRRFTEITTATAVGKTGCFDVFECKTCNRTFPSFQALGGHRASHKKPKVNVEEKKSNSVNIQIPPPLTEYSEEE
DHVQQKSVVEDEDNKTSIVISNKGKVHECSICGSEFISGQALGGHMRKHRPIPFSGNRTGLMMNTDGSSTADQIIEKSPGSSSS
SMLSLDLNFPPPEMADNDQSEFQFTGGSSQRHLVFSAPALVNCHY
>SrC2H2.3i-Mx.03
MYFEASPFFQCLNPSSTSSSSSSSSPSSSYDTMMFSKEQDFPYETIQCLPLLNRLLDRDDNDTVLKIKEEDNTDKVTISLHIGL
PSYGTETSGDSVVSNKLFDEFEEEDERKKSDIKVDFDNDHNNWYNVNMSDHDQTRFWIPTPSQILVGPMQFVCFLCNKSFNRYN
NMQMHMWGHGSEYRKGPESLKGTQPAAMLRLPCYCCAQGCKNNINHPRARPLKDFRTLQTHYKRKHGAKPFTCRKYCTKQFAVK
GDWRTHEKNCGKLWYCTCGSDFKHKRSLKDHIRSFGNGHSPRSFPEGFDHDE
>SrC2H2.1i-Q.03
MEKSNRETHDFMNVESFSQLPFVRPLSLKEKGIRLFGKEFGSDPDRRPDSTGVTTDEFDTINNSDTSDICVVDREEFKDTNEIS
RKFECHYCCRNFPTSQALGGHQNAHKRERQLAKRAHLQSTMVNGSFSEAQMYGLMNYHRFTTTPPSFYHQTTTSSTTTNYNNRF
NYGHNSSYSSHQTPINGSPLALWKYPNYAQNRMINTSPLISSSNEGLRASRIQTNSSYMYDSKPSVQDQVSLDLRL
>SrC2H2.2i-Q.02
MEAHDDHGDTMIMGVMKSHMMIKGKRTKRRRPSPALTLTLATTSSSTTTSASETSVPTNHNGFFQMPTTTTIEFTNIVQDSHTT
NDQELASCLMLLAYGHHPLAQFPSVPVAPRSYVYECKTCDRGFASFQALGGHRASHNKCKDVYELATNKTLSCNPSGSTKGPKV
HGCSICGSVFSSGQALGGHMRRHRSMTMATTTSSTVATTCNTSNERHESKKHKTLLPLDLNLPAPIENDHKETDNSFGSNNQII
VFSAPALVDCP
>SrC2H2.2i-Q.03
MEAHDDHGDTMIMDVMKSHMMIKGKRTKRRRPSPALTLTLATTSSSTTTSASETSVPTNHNGFFQMPTTTTIEFTNIVQDSHTT
NDQELANCLMLLAHGHHPLAQFPSVPVAPRSYVYECKTCDRGFASFQALGGHRASHNKCKVVYELATNKTLSCNPSGSTKGPKV
HGCSICGSVFSSGQALGGHMRRHRSMTMATTTSSTVATTCNTSNERHESKKHKTLLPLDLNLPAPIEDDHKETDNSFGSNNQII
VFSAPALVDCP

>SrC2H2.1i-Q.04
MSDQEQLLSSSDHPLKIHLTSDDHVNDQEDEEEEDDDDETKIESSDGGHDINDSKVNHEELNLIDSLNMMGNCNSPATKEAADT
SEPRVFSCNYCQRKFYSSQALGGHQNAHKRERNLAKRNQRMGFLSGPMISAAAAFGHTYMHPQHHHHQNYCSTLSSLPLHGYNN
NNNRSLGIQVHSMIHKPANSIFPTSSSWSGFKGFYGNKGWPSRPHADQQPAIGKLNMTRSLAPTAPTWASGAHGGAAHFEVVRT
GMSSTAEDEIGGFWWPELKTNQEESHKLDLSLKL
>SrC2H2.t2-Mx.01
MEEEMTETKRELPIPSDIRRYTCAYCGIVRSKKILISTHIQSHHQDEIKVEGGGEGMVNVCKECGVSFRKPAHLKQHMQSHSLE
RPFICPVDDCNISYRRKDHLNRHLLQHEGKLFNCPIENCKKKFSIQGNMTRHVREIHDDVESTVDDVDDRKQHACLEPGCGKVF
KYASRLQKHEESHVKLETVEAFCAEPNCMKYFSNAKCLKAHLQSCHQHVTCEICGIKQLKKNIKRHLRTHEKIVSKDKIKCSFD
GCNSTFTTSSNLQQHVKAAHFQEKPYVCSISGCGMRFSFKHVRDNHEKSGRHVYTLGDFVEDDDQFQSRLRGGLKRKLPDKIDT
LMKKRVLPPDEFDTIHGSDYISWLLSTEDND
>SrC2H2.4i-Mx.01
MEEDQDELRFVCKLCDKRYPSGKSLGGHMRSHVIAANSSESDEKFTTLMTNGNGIETRNPKKTWKTVDSSTIPYSNEKICKQCG
KGFQSLKALCGHMACHSEKDRNFKDYDHSWTSENLDHDEKVVIDSYSDIEEPEFQDPARVTRSKSKRYMQVIVKPYSFNYNYGS
SSVSEIDELEQEDVAMCLMMLSKDSSNWGGVNSVVETSDNDSGVLEMNSDSRFYGNGNHTSGKLESEFSIEEWLRNGDRNKSKL
TYEQKLEIRRNLFKEFAYRDSLRKNTRVEDDSYTPELEREGSNKKRNKYECLNCNKIFTSFQGLGGHRPCHKKNNSFGSKNNND
EDRLESEFAANRKAKYDNKIKAKRIKGHECPICFRMFKSGQALGGHKRSHFLNRGIDHYAVMEHEAPSCSDMIDLNLPAPEEE
>SrC2H2.1i-Q.05
MYNMEESDHYQSKNTNNTRLKIFGFNVSEDEEVESSKTPSGSPESATTDGRKYECQYCGREFANSQALGGHQNAHKKERQQLNR
AQMQASRNAAVSYIKNPMISAFAPPSHLLTHTGPMVVPSSSVASPSWVYVPRAAPSFQVLSSASSSPSGARGPESVPYASVTNV
GPQPSNRAHHDDRFGPSFDDRFGLDLHLSL
>SrC2H2.t1-Mx.02
MSTTSESNVCMECDEMDEDSVLLSLSPPGQPNTSTYANTWIIIIILKLSTTKPATSVGDQEGGSSYVTNPNINIASDHLQYWIP
TPAQIMLVQHSSLAQFCNKTFNRYNNMQMHMWGHGSQYRKGPESLRGTKPSSSMVRLPCYCCAEGCKNNIDHPRSKPLKDFRTL
QTHYKRKHGEKPFACRKCSKPFAVRGDWRTHEKNCGKLWFCVCGSDFKHKRSLKDHILAFGNGHGPHYTSEDLIDDEHEDENVY
DEDKKSISNGGHDCRFFMI
>SrC2H2.t1-Mx.03
MASGSSSSSMLFVGTMEIEDNMEVTTYSQQEWNLEQQASLFCFIYEDPDADLVIAVSPTTLLDTNKYVCEFCFKGFARKQNLLL
HRRTHNIPFALKAKAPNDHVPRKVYLCPEPTCVHHNRSRALGDFGGLKKHYLRKHSTEKMYKCDTCRKAYSVESDLRSHSKICA
ERNNNNHRGNLFSRENNFTTHQVDNDARDPQTIYMGDDSVKSIINDNIPIGSSMPYQQTLGTENNTIINNSNENINTLFDPQIS
WIQNQTNSTPFESQLCQFEVNHSSFMTNTSMGVNMNQTPPEFFPPTSTRINPYGSQRTFDIGFYADLLAADKMPIQNANLDQDM
SLMGYCGNTYRTIEGSNSILDGGYMRDPISDFHMTQAMDNLSTYGLQNPVIGSSDGGCYDQMVYQDYDYGVGSGQLSYGGGLME
MFKNDYHHQQRQDDN
>SrC2H2.t1-Mx.04
MYPNADVIAISPTTLLDSNRFLCEVCYKGFSRKQNLTLHRRAHNLPFTLKTTTPKDPVQRKLYVCPEPTCVHHNRSHALGDFGG
LKKHYLRKHCTDKKYKCDTCYKAYSVESDLRAHSKTCTKKKHLCHCDSEFSRGYHLNVGEIKDPQTLYMGESSIQNTRREDIPE
GPPISSIKNQSNFYSCVQSQFCQFGADQSSLKTYPSMSLNLVETPAFFSPTSSNHNQHGCQENSNIGLYANLLLDKIPLQNVNL
DMSLMRDSGYIYGHVEGSKSISDGGFKLDPNSLNITQTMHTASFNGMMNPMICSSDAVRDSYEQTGCSGYGGHLNYIGGMTVLN
YERSTTTKGSLAPSEQHPNQQGFWF
>SrC2H2.t1-Mx.05
MLGNNNHHHITAPSSSSNPFLSPENDLSSKRKRRPAGTPDPDAEVVSLSPKTLLESDRYVCEICNQGFQRDQNLQMHRRRHKVP
WKLLKRESPEVKKRVFVCPEPSCLHHDPCHALGDLVGIKKHYRRKHSNDKQWVCEKCSKGYAVQSDYKAHLKTCGTRGHSCDCG
RVFSRVESFIEHQDACIVRRVHPDIPTFQPACSSLTASSTSPSSDTNPNTIRSLQRVTQIPLSETPLLHTHDTQNNLELQLLPS
SSSYEQNNVRSTNLKLSIGSEITFTKEEEKMKMAMVEKEFAEEARQQAKRQIENAELEFANAKRIRKQAQSELERAKSLREEAT
NKISSTILEITCHSCRQRFQTGATITTDEMSSAITEGEGD
>SrC2H2.4i-Mx.02
MANANDQHSNLKHFCRICKKGFMCGRALGGHMRAHGLDDDTGNLDDEDPTSESEDKLGNQRMYALRTNPNRLKSCRVCENCGKE
FLSWKSFLEHRKCSSDDGETLVSSPESEGVEQKGYDDENHGNRRDCGWLKRKRSFNSICPSSEDEDLALAKCLMDLSNSRADTT
DTDVEDSCASPSRVEQRRNPTCTTDACMAPFMRPASSLDEAKGVAINTNGMFACKACKKVFTSHQALGGHRASHKKVKGCWHET
IYLMKTSDDIMHDEFILSPKPISSYPFHDGPGTTSLAGVAHRKAKVHKCSICDRVFASGQALGGHKRCHWLTSNMSDTSSIAKI

NFHEHIEQLHQRALALPSRILNQSKPLNLHPPVSEPYIAGPRKDPERYPLSLEVSTEINLHSLNVDHKHQSYKIGDQEHDHKNT
NHHDIKGEVGATMEDDEVDSKLKLAKLSELKDMSNISGSSSSWLQVGIASTTDVGPPCDS
>SrC2H2.1i-M.01
MDAQAKKKALYRAKLKAQKQDKRIDSPLVRYNESDQPVCKVCDIVLKSESAWSAHQISAKHREAIKNVKANAAAASQANNVKPV
GPTESNKPKPDRSSESYKSQTRPPQPQPQSALPSNFFDNPTTKTKQNETSNAKLNDTDKKKKLVSFDVENKRVEISSDIVAETT
NDETQASLTRALPEGFFDDKDADLRARGITPVKLDIKDEYKEFEKVIQEDLKEVDNRLEEEEYDAAEMIEEAETVEQKFCRERV
EMFKRKKMELKAARSAKRGKNLQVVEKEISNDESSSDEDSDDDVDWRAKHL
>SrC2H2.1i-Q.06
MALSKSINLEIEFHEDDIDSNTAIHRTTKTTYEEVSLDLSLNFNSNIDKFERASSTNETESTTTTTTVSRVFPCNYCQRKFFSS
QALGGHQNGHKKERMLAKRAMRMGMYSYDYGNQLADFSSHGTWFRSLQIEAHSSHHQTFAPPVMRIPKESSIMLPKQPNGFMGL
PTYVQDDEPDMVFWPGSFRQVAATSVDVGSQLETSEPRMVEGIRPVYDCHATPDLTLRL
>SrC2H2.2i-M.01
MNGDQEQKCHSCRFCKQNFSNGKKLGGHMRTHLALISSCRKQGTQESNQEAINVEKALDVHHFDDRIIMRREGINRDYDTDYRK
HTKQSIQESDQELINGGQGLEIDDVMIKKSDQEYGANVHVLRENPKRSWRVSSSISDSSRITHKRFDSRKESMKNAACKQCGKV
FDSMKALYGHMRCHSIKRSTESSSSMPSSEEHDDDDEVENGALCLMMLSRGVTNLDGIKLIDSNQAKFSVLKMDLIVQSGSGQL
NKGQTRSISMNFKSTNKRKSCHQDLTLKRRKR
>SrC2H2.2i-M.02
MDEEMIAYHNIMPVELAIKREMEYRTKIEVLKNRRLINLNPLLPSQAQPSRLATLKRKEPSSSGTSLERLSTGLVCKICQITFN
TVTHLKQHSYTLKHKGNVLQLKKRGQNVSTPFLCELCNSSCSSGIIMDEHLKGTRHLTLLQELENVKRSRSE
>SrC2H2.1i-Q.07
MGKVVFNLLSSTRDRAHVKNSQYPLIISHKKIRLFGFEFDPNKSQLSSPTTISSDKKKHFMETKKFKCQYCFKGFVNSQALGGH
QNAHKTERMKKKRLLLQARKAAIEYHLQPYDQIVNNHGINISFGHCVDQEFSKPDVTFGLEDEDVVTFQDRCNSGNNHTSRSIT
MISSSSDNSKESYEDLDLQLALSSYSTK
>SrC2H2.1i-Q.08
MGKVVFNLLSSTRDRAHIINSQYPLIISHKKIKLFGFELDPNKSQLSSPTTISSDKKKYFMETKKFKCQYCFKGFVNSQALGGH
QNAHKTERMKKKRLLLQARKAAIEYYLQPYDQIVNNHGINISFGHCVDQEFSKPDVTFGLEDEDVVTFQDRCNSGNNHTSRSIT
MISSSSDNSKESYEDLDLQLALSSYSTK
>SrC2H2.1i-Q.09
MSFLSLKLPEDDPNWSSSSYYSRLSYHEQRVFSCNYCRRKFYSSQALGGHQNAHRTERNLAKKNRKLGSTDDRYHLGYMSHEPT
SKPGSSGSSHRGLVQPPVMMVVNHQEHNVGSGDNNFGKGLDQEDFQLDLSLKL
>SrC2H2.1i-Q.10
MSFLSLKLPEDDPNWSSSSYYSRLSYHEQRVFSCNYCRRKFYSSQALGGHQNAHRTERNLAKKNRKLGSTDDRYHLGYMSHEPT
SKPGSSGSSHRGLVQPPVMMVVNHQEHNVGSGDNNFGKGLDQEDFQLDLSLKL
>SrC2H2.1i-M.02
MPVAKLLGASTPDAMKTDEGNDSLDTIIRQAVGKEPLFSFSRNGDSPTQWIQLLHTFDQPDLPGWPLLAPMKVQLQKCDKCSRE
FCSTINYRRHIRVHRRSMNFHKDPQKYRDLLGAFWDKLNYDEAKDLMSFKDVTLEEVPGSSIVKNIAANLRKPIFLSLPQVYVK
AGSALVDIIQGRPSMLPVSSQELFSVLDDASERTFLCAGTAESLKKYVFDGEAGKIGLEMKNLIACTSFLVEQILVKAWLADKD
AEALKCQKLLVEEEEAAQRRQAELLERKRQRKIRQKEHRAREQSNGMKEELSTASDIFESIASAENSSHQIDPLIPDENITSSF
DHTQLSSNEEALDHADSAVNEQHKTNNNNTSQHFIARWQVPKSQRNGRHNNQNGNFVKWEPTHKHREQRLNNGGKIWTKKPKPE
KYYCQLMIGSISVTVKNSTSQQQVNSSAAEFQGNDNTKSGQNVPKRSNLKLWRTRHENRGKLADVCKNQTLEEDVASTRGVDQT
LTADLSLDLNGNNREDTNVQVDGGELINFSIDAAKDFLSQRWKEAVSGEHVELVLTTAVKVDVEDDHPESAKVQAGDANVKPKF
RTKPDKNIKTKYIPKQRPPVY
>SrC2H2.1i-M.03
MDVVGSNKVHDPVKNANGYSKMSSCSSICSFRDVVHGNTRVVHRADNSPESSTVGMDSGLLSRKKQLTDGGSSSSSKSLSRSAR
STDHGSSGSSSRSMQFRKLSGCYECHAIVDPSRYTIPRSTICVCSQCGEVFPKMESLEHHQAVRHAVSELGPDDSGRNIVEIIF
KSSWLKRDHPIFTIERILKVHNTRRTIQRFEDCRDTVKTRATATASRCAADGNELLRFHCTTVSCSLGARNSSSLCSTVPGCGV
CTIIRYGLQSPKAGDNGIGLQSPKGGVRTTASSGRAHDCLGVDPHGRRAMLVCRVIAGKVKRVAENALTEEDGSYDSVAGYGGL
YSNLEELYVSNPRAILPCFVVIYRVPES
>SrC2H2.1i-M.04
MDIEDQRKPTTPSGHENLEVHVCHRCRWAFPKPNPSVKRRRSHNRICGTIDGFDNIIDSKANSDDDDHHSDSDSDSDVQEKDKT

PSHEVHICLKCGWPFPKPNPSFKHRRAHKRICGSIEGYTKLIINSDTVSDDENQSDSDDEQQEDKTPSSKIEKKIINESSSSVG
KTEEKSVRSEEDMCSALTQFSDTQISPDVTSEFKKAVKFEEHDRNTKRSVPLVKDTDGSHNKDQVENVHKLSEPIDIPIVDKVE
TLMEDFKDKKTSHSNAYRGDKQAENRSVVDMFEVKTVHERVHERQTSEHERVQEPKSGKLLTGVDASFKWSNSSLDGNWGSVSG
LSDVFGKSELSDDKSRLGRSDAFEGGSLVKSDQEIIEQKASEIKANEEAITKVNNWSTSEAKSSDPKRLSNLIKKIEDTAINSE
GQKSNLTQETPKVNQERSNLTPKTVKVNHKRSNSQGNPKVNHGRSNLTQEAPKVNQERSNLTQETVNVNHKRSNSKETQKSTRD
GQL
>SrC2H2.1i-Q.11
MEPDPHNFINIKSSSQLSFIRPSPLKEKTIRLFGKEFVGTAGADSSTGIITDDSDSNDTTFIREQSKQTARECRQKFKCRYCCR
NFPTSQALGGHQNAHKRERQLAKRVHRHSVMFSGGLTAAQTYGRMNYHHLPWYHHQQPPYWGTTNGSPTAMWRFPAAGKSSLSD
TLVTRYPYEQSLVVQDQVSLELRL
>SrC2H2.1i-Q.12
MEPDPHNFINIKSSSQLSFIRPSPLKEKTIRLFGKEFVGTAGADSSTGIITDDSDSNDTTFIREQSKQTARECRQKFKCRYCCR
NFPTSQALGGHQNAHKRERQLAKRVHRHSVMFSGGLTAAQTYGRMNYHHLPWYHHQQPPYWGTTNGSPTAMWRFPAAGKSSLSD
TLVTRYPYEQSLVVQDQVSLELRL
>SrC2H2.t1-Mx.06
MTTDVDSSSAVNESTVSGEASFSSPAKQTALPPEKPVKRKRNLAGMPDPDAEVIALSPKTLMATNRFVCEICNKGFQRDQNLQL
HRRGHNLPWKLKQRTSTEIRKRVYVCPEPSCVHHDPTRALGDLTGIKKHFCRKHGEKKWKCDQCSKKYAVQSDWKAHLKICGTR
EYKCDCGTLFSRRDSFITHRAFCDALAVESAKGQPEDETLKEDKDPKSVFIESTPQSTSTASPPPPPTPLPLPPPPPPPVAAPA
IPVTSSVLCISKTPDLPEISKLNQDSRPVLDNPPQLLAGQPGSCNRTSSSTNTGSTSSVFASLFASSTASKKFQPQPTGFKDLI
QSMTRPTQTPDLTLLSPPQEPVSLCLSTNQGSSIFRTAGQELRQYAPPPQPAMSATALLQKAAQMGASASGASLLRGYGLERDD
TDGGASIGLGLGMGYDGRSGLQELMMGTPSVYGPKHATHDFLGLGMAVGGGTTNGLSALMTSIGGSLDVTSAVSSFGLGGTGEF
SGKNMGPQ
>SrC2H2.t2-Mx.02
MSNDNSYPTYLNPSWSILKYPHCQYISSSSSSSSVHPPSPPLKAALPLLSLSPTKQPNFYPQNPSTITDACSTTTTATYTTPHI
CPESSVTVSLHLGLPTLTLSEADLISRFSTNYAHVSDDHKEDKIGTNINGYVTSTINKGQYWIPTPSQILIGPNQFSCHLCCKT
FNRYNNMQMHMWGHGSQYRRGPESLRGRQPTAMLKLPCYCCAPGCFNNIDHPRAKPLKDFRTLQTHYKRKHGDKPFMCHKCDKM
FAVRGDWRTHEKNCGKLWYCSCGSDFKHKRSLKDHVKAFGNGHAAFEFVDCCFEVDEDEGASDIEQ
>SrC2H2.t1-Mx.07
MLSGDGFSSPPSSLSSFIHDPVPNPNPNNNSSVSGKRKRNQAGNPDPEAEVIALSPTSLMATNRFVCEICNKGFQRDQNLQLHR
RGHNLPWKLKQRNKNEVVKKKVYICPEKTCVHHDPSRALGDLTGVKKHFSRKHGEKKWKCEKCSKKYAVQSDWKAHSKICGTRD
YKCDCGTLFSRKDSFITHRAFCDALMEENSRMASRSIVPSMGSLGYRSDLMMMMNGGGDGGGGSGGERPIQAVFGGGLESNLDL
NGGKARLPIWLDHNANNHQLDNPNNSTFLGSSSSSFNNGGMLPPEMLNWLGRFNGQPQGLSLKEEEHAQKEIQINSMYNFDSST
SQNNLMCSSMPPVATALASPVANMSATALLQKAAQMGSTRTTNSVVSGGNDTGFGLMTSLRNGDRLMMMADRTDVPPPTSKGFS
GSEFGDGDFTRDFLGVERNERSVSYSLQQELNKFACSTMGFNQVNRNQE
>SrC2H2.1i-Q.13
MDQSRFWIQNEETCFNRPPRVFGDSWEEQAFAEDATGPLGGCIWPPRSYTCSFCRREFRSAQALGGHMNVHRRDRARLKKISTS
NNNQVVVETHHTSDFDICVQLPNPNFDHHHPPLLSSSSSSTSVSILRPFTCSFGRQQHQKVEPEKTFHIADFGNQDSSKVLETA
IKKNNHNQIELHLASDINSLIRKRCDIDDTPETFSSKKDDASQDHGLIFKKRRIEGGVLQSFLPRNSNLLLNSDEVESVSPGSE
LTTNTTSDNLDLELRLGDRPRSSDVRV
>SrC2H2.1i-Q.14
MEIKNSESTIPFQDFVSTINGEFGEEGRSFTCTFCKRGFSNAQALGGHMNVHRKDRARLQETLITTKGVNLNSMDQLHEARSCD
DHDDDKGVSVTKRPWILKDDQGVNIKKPTLQLSLWVESSPTSDSRIRSDIFSSSSLAEVDLELRLGTDSDTTDHAIRD
>SrC2H2.2i-Q.04
MTVKRLLEDNIEIENIDMANCLMRLSRVGNSDPTPEPDRLFRCKTCNKPFQSFQALGGHCASHKRPKVNESIGQLKLKPKTHEC
SVCGQEFALGQALGGHMRRHRDDAATVKTGQMLVKKQSVTTGLCLDLDLDLKLWPVFS
>SrC2H2.2i-Q.05
MTVKRLLEDNIEIENIDMANCLMRLSRVGNSDPTPEPDRLFRCKTCNKPFQSFQALGGHCASHKRPKVNESIGQLKLKPKTHEC
SVCGQEFALGQALGGHMRRHRDDAATVKTGQMLVKKQSVTTGLCLDLDLDLKLWPVFS
>SrC2H2.2i-Mx.01
MANLHPIFKNFISLTFILLHLGCFIIFPTSRHHRHRPKPPRKPAAVVTSLRVAATKLKCHKSISSSWSFIKRIFSSKPTSQLIT

GNNSIFHEIPSTDMITRSISTTRPGSLTGSDYFPIRNDTHEIHPCLNCGEIFQKPGLLEQHRSIKHAVCELSDDDPGKNVVRII
CESGWVGKPPTIYRVMKIHNSPMILARFEEYRESVKLNAVLRQGGSGRRNERCIADGNEQLRFHCTTFLCDLGQNGNSSICNHQ
YCSVCRIIGSGFSPKLDGISTLSNSWRAHVAIPDDIEDEFRFMNVKRAMLVCRVIAGRVGCDPEIGDKDDPGYDSLIGRESGVV
QTRLDDEDELIVFNPRAVLPCFVIVYTV
>SrC2H2.2i-Mx.02
MEDGNIVHEEHKSIKCFPCQFCSRKFYSSQALGGHQNAHKKERSMARKAKRATVINNNNNNIQCNYSTCLFPQSPMFFAPTNHV
GILNPSIYINTHANAHAANFCQFQSQPFGADNIMFYRGNYINQFNREDEHQSFLNSSRDMFVMNESKGVEDKDTQKLDLSLHL
>SrC2H2.t1-Mx.08
MSNITADEGSFSSGEEGQEKQIHDLKTSLHGGSTSNVSTALPPPPPKRKRNLPGTPDPTAEVIALSPTTLMATNRFICEICNKG
FQRDQNLQLHRRGHNLPWKLRQRTSTDARKRVYVCPEPTCVHHNPARALGDLTGIKKHYSRKHGEKKWKCDKCSKKYAVQSDWK
AHQKTCGTREYKCDCGTIFSRRDSFITHRAFCDALAEENNKVNQEVLQPQHLNDHMLSSKFNDSRNKGLMGLPDFNSYDPKTQP
KSLSEDLIPMPFKPLIMCEGKYSSSSGTLFGNIRGGSSSSSGLQLSAPNGTSGYGYLQNESNDGGLLSGPPGHMSATALLQKAA
QMGATASNGINSPMMQKSFVSSMSVPGELRSTGLIPSPYGIHRARTYDNFHVQSNQTTMAGLNASNEGYNHFENPNQQDMFGQG
FGTESSRVMNDIGIYSEMLIVGDQSNGFVKNIENYEDRSVDNSILIHGSGNSGTSKLGGGQCRNDTLTVDFLGIGGSRPPNIQE
QQRLNGFESSSQVMNPFQQLIHGESSGHEKPVWDE
>SrC2H2.t1-Mx.09
MADLEPAPPKTLMITKRHKCVSCFQQFKKKKHLVAHMKISHHSVHDPKCGVCQKHCKSFESLREHILGPLAKSQCSSIFSQYGC
QLCMEVFENPIILGEHKDQCLLTAPMFLGTINIPNIDYEALEDEIYISKCFKAVAIDCQMVGCGSDGSLDLLARVCLVDEDENI
IFHTYVEPQISVTDYRYDLTDLTEEHLTDAMSLKEVQEKIYKILYNGESIGKARLDGGKAKLLVGHNLDHDLDCLLMSYPAKLL
RDTATYRPLMKTNLVSHSLKYLTKTYLGYDIRLGKHDPFQDCVSVMRLYKRMRSQKHQDDKSATSSCAKQNLNFETATSKDLEN
MSPDELFRISTPNYQCWCLDSN
>SrC2H2.1i-Q.15
MEPDHSFPESSDDAITSTTFEESSTQTSIIGGRSYECNFCKRGFTNAQALGGHMNIHRKHKAKLKESPSSPPPNAAATTTSNPF
PSSPQGKKKPLSLFGEGLYDSSNINQENIVTVDPPSSMPEVDLELRLGHTRSSGNKITTTRKFF
>SrC2H2.1i-Q.16
MESHNNISEPEEESEINNQIPTTVSESTPPLQPVTLDLTLSFNPMMVVDHAATELPPTNTATGSPTPRVFSCNYCRRKFYSSQA
LGGHQNAHKRERTMAKRAMRMGMLSEQYPSLASLPLHGSTYHSLGIETHGSFHQRIVPQETGFYTVRGGSRFNQQPYVRLPMFM
EEDEPEIFWPGSFRRIDGVGGSSMVPPTGRDSTATPDLTLKL
>SrC2H2.t1-Mx.10
MEATSSSTSFFGARSEDQEQNVSSIIPSTDSNHKKKRNLPGNPNPDAEVIALSPKTLMATNRFICEVCNKGFQREQNLQLHRRG
HNLPWKLKQKSKKEVRRKVYLCPEPSCVHHEPSRALGDLTGIKKHYSRKHGEKKYKCEKCSKKYAVQSDWKAHSKTCGTREYRC
DCGTIFSRRDSFITHRAFCDALAQESVRHPSAIGSIGSHLFGSHLSTSMNLGRSQLESHVPHLHEQSNNQTSTSNILSLGSSNF
DQVIHPQGQTHLFSQPQHQMSNTTSSYFLSDTNHGLNHVFDYDQQNHQHGLMSNKPNHIHGLLQLPDLHSNMTKIPPNLFNLGF
FSNNINNPTNDLRNNDQNHNTNFTSSGFIIPNHRFNIGGNSNGNSNDFAFINSNNQHQDVQMLPQMSATALLQKAAQIGSTTSN
HHKIVGGDEKSQTQSEMVSDHNQLQGLMNTFRSKYGDSTMFGGYDGCNNRNLNFGNQNETGNSNRPTLDFLGVGYSQKEQQHSS
HLNINSLDQKLDSSAQTSQLHGGSSKMIGN
>SrC2H2.t1-Mx.11
MDGGNKHDKALCSNEINHELYDSQQQRRGNSVVHDYEAMIRKHIPSSLTQINDQRRSQLWDPKTMLSNLSIMEEKIHQLQELVQ
LIVRTTQPNQLVIQQQQLVTADLTSIIIQLISTAGNLLPSVNNPHFPAPAFGNEINVPDNKYNDNDSNNYVSDSNTIKNIVSKV
EDDSNDMDIHDEDVDDLPPGSYEILQLEKEEILAPHTHFCVICGKGFKRDANLRMHMRGHGDEYKTPAALAKPHKEIGSEAKLI
KRYSCPFVGCKRNKDHNKFQPLKTILCVKNHYKRSHCDKSYTCSRCNTKKFSVIADLRTHEKHCGRDRWLCSCGTTFSRKDKLF
GHISLFQGHTPAIPLDAFIHDNAQGDHDTNTYTKLILILILILILILN
>SrC2H2.t1-Mx.12
MDGGNKHDKALCSNEINHELYDSQQQRRGNSVVHDYEAMIRKHIPSSLTQINDQRRSQLWDPKTMLSNLSIMEEKIHQLQELVQ
LIVRTTQPNQLVIQQQQLVTADLTSIIIQLISTAGSLLPSVNNPHFPTPAFGNEINVPDNKYKDNDSNNYVSDSNTIKNIVSKV
EDDSNDMDIHDEDVDDLPPGSYEILQLEKEEILAPHTHFCVICGKGFKRDANLRMHMRGHGDEYKTPAALAKPHKEIGSEAKLI
KRYSCPFVGCKRNKDHNKFQPLKTILCVKNHYKRSHCDKSYTCSRCNTKKFSVIADLKTHEKHCGRDRWLCSCGTTFSRKDKLF
GHISLFQGHTPAIPLDAFIHDNAQGDHDTNTNIQGSKEVEFFDFNFNSNGSSGSGIGEPSACYSPFQGSMFEESDSLSFLSSGS
GNYLWKNGGESSSKDLQG
>SrC2H2.1i-M.05

MEFWGVEVKSGQPLKVNIKEDKVLHLSQACLGEIKNNKSNESIYLQINIDNKKLVLGTLNSERLPQQLFDLVFEKDFELSHNWK
NGSVYFYGYMAEQPADDFSDYGEESDDDEELELPIPNGKQEVKKDGKQKVKIVEPEKGKKADEDDSDEDEDGSDSDEDDDESGD
TDDSSDEEEETPQKQQSGKKRPIESAAKTPANEKKAKLATPQKTDGKKAGGHVATPHPAKQGKTPANKSNQESPASASGGAHSC
KSCNRNFKSDMALQSHNQAKHK
>SrC2H2.2i-Q.06
MEANQDDYCTIMANSRNMIIKGKRTKRPRPSSPLSLTIASTSSTTTTTTTSTARSDDNGTENYPNNTFHTTTDSIEFTKIIQEN
DEEDMANCLILLAQGQASHSPHKSGLYVYECKTCNRGFSSFQALGGHRASHKKPSKPNLEDKIMPNMVTHDVHVDHRSSIELSL
QIGSSQLTQCNPSGSIKVVKIHECSICGAEFGSGQALGGHMRRHRSMPLARTSCHYYNGCQEFKKPKTLLSLDLNLPAPVDDDV
RETKFPFGSKDQMIVFSNSSLVHCHI
>SrC2H2.t1-Mx.13
MMFSASVYNNSPISNKTSTSVSEEAATTTVISPVITSKIHHEFTPSIALSSISNHIQEHHHLYPNTQKIKKKRNLPGNPDPDAE
VIALSPKTLMATNRFVCEICNKGFQRDQNLQLHRRGHNLPWKLKQRNNKDEIKKRAYVCPEPSCVHHHPSRALGDLTGIKKHFC
RKHGEKKWKCDKCSKIYAVQSDWKAHSKTCGTREYRCDCGTLFSRKDSFITHRAFCDALAEETARLSAAAAAAAISVTNNNHPS
TINNLTQTPFNFQLSSQNPPSSLFPFSPNPTWIPTQNPTQIKPEAIHHQIPAISLPFHQEQEKGMMVVSRPPVTFSGCHVSTGY
HHLATHLSATALLQKAATVGAVGDHVGSTMSGLDMGELGHVTTAGVIPPEYHQHVGLSSGNVYSWQKTDRLTRDFLGLTGDQED
DQHGGGVEGNVHVRGMLTYAGGIQIPANGFGASDTEAWGNC
>SrC2H2.1i-Q.17
MDHKLKSKWEHNNFIFNKDYTYDGSSWRQRSFMCNFCKKEYKSAQALGGHMNVHRRDRARLRQSSPSLDHPNPNPNFSSPSSTS
SIRCLPFKTFQPSLFSLDFSSPSLSLNNDQEKQKMFPFVSHSAWNFLGDVRKNMNTDQELVASANRGNFNDNFVDEENESRVWK
KRESFRVEMESGLLKDGKIEMGLDLELRLGRS
>SrC2H2.1i-Q.18
MGHRLDRRLKQMEGENVVHDEPKTMKSFPCQFCSRKFYSSQALGGHQNAHKKERTATRKAKRAVNNNNIQCNNFTSMISQQPPL
LFAPTNHHIGLFNPSVYNLWQFQGQPFGSDMTRFDNIMFYRDSYINQNHYNQFDQEDENQKLLNSPRNMFVMSESKGIDKDSQK
LDLSLHL
>SrC2H2.2i-Q.07
MAIKRSWDDDREIENLAMANCLMLLSRVGQSGSSPGRVFNCKTCNKEFKSFQALGGHRASHKRPKMTDGNDIQLPVKPKTHECP
VCGLEFAIGQALGGHMRRHRDGGDEKSGRREVANRPPVTEKRGLFMDLNLTPYENDMKLWSSSVNTALAM
>SrC2H2.2i-Q.08
MAIKRSWDDDREIENLAMANCLMLLSRVGQSGSSPGRVFNCKTCNKEFKSFQALGGHRASHKRPKMTDGNDIQLPVKPKTHECP
VCGLEFAIGQALGGHMRRHRDGGDEKSGRREVANRPPVTEKRGLFMDLNLTPYENDMKLWSSSVNTALAM
>SrC2H2.2i-Q.09
MVKRSLEDDTEIEQMAMANCLMLLSRVGKPGSLSEPGRLFYCKTCNKPFGSFQALGGHRASHKRPKLNDFRSQSPLKPKTHECL
VCGQEFAIGQALGGHMRRHRDEVTGKVVPVTVKKESETTALCLDLNLMPNDWKLWPIVT
>SrC2H2.t2-Mx.03
MSKSNKNDRTVVICPLCAKGVHLIPNQDPNITWESHVNTDCDPSNYEKATKKKKCPVRGCRETLTFSNTIKCRDCTIDHCLKHR
LGLDHGCPGPQKPEPTFPFWGFSSSIKQNRQPSRATSARAPSSSSSSSWTTSLLNAVSSLMVSDDRSGEVGQNGGGSGGQVEEC
PICNMKFSKVAKLIDHVQMVHEKKGVMKVTVDVCPKCSKGFKDPVALVEHVEKEHRGVSKA
>SrC2H2.t2-Mx.04
MGTPAFPDLGKHCSVDDCKLIDFLPFTCDCCNKVFCLEHRSYITHKCPKANKNDRTVVICPLCAKGVHLIPNQDPNITWESHVN
TDCDPSNYEKATKKKKCPVRGCRETLTFSNTIKCRDCTIDHCLKHRLGLDHGCPGPQKPEPTFPFWGFSSSIKQNRQPSRATSA
RAPEPSSSSSWTTSLLNAVSSLMVSDDRSGEVGQNGGGSGGQVEECPICNMKFSKVAKLIDHVQMVHEKKGVMKVTVDVCPKCS
KGFRDPVALVEHVEKEHRGVSKA
>SrC2H2.1i-Q.19
MEIRNLESSQDHLVSTNTINNNINGEEISRCYTCSFCKRSFSNAQALGGHMNVHRRDRARLQAESTCTTNLNLNLNSAVDRSLD
DHKSHVSILKADDDHQHRRKKDLDHGVFNSRKPILQLSLCIDSSSNPSNQVSSLSSSPTDLDLELRLGMDSETLSRVKRD
>SrC2H2.3i-Mx.04
MNNHTITTTPTSPELQLRPASPDPHDVFRRTVDDADVDVSGWNLQIQPITTVNPDHHQNPRKKRTKTIRFNHNTKSIVSGGSGP
SGSHSGVITKKKPDQSAPKITRPCTECGKQFPSEKALFGHMRCHPERPWRGINPPANIHQPPPAKNDVTRFITEEDEYVAACLL
MLANTPTVSQTFHTTTGTTITSTSYHHNSGSQDIFPVQINQVNDSETRFECSSCKKVFGSHQALGGHRASHKNVKGCFAITRPD
SGPGSGPEDLEYGELLSMNQQHKCNICLKIFSSGQALGGHKRCHLEKEEGVLPTTILSISQTFKFDLNTPANLHDDSISSTSDL

DLRLCL
>SrC2H2.2i-Q.10
MNNHTITTTPTSPELQLRPASPDPHDVFRRTVDDADVDVSGWNLQIQPITTVNPDHHQNPRKKRTKTIRFNHNTKSIVSGGSGP
SGSHSGVITKKKPDQSAPKITRPCTECGKQFPSEKALFGHMRCHPERPWRGINPPANIHQPPPAKNDVTRFITEEDEYVAACLL
MLANTPTVSQTFHTTTGTTITSTSYHHNSGSQDIFPVQINQVNDSETRFECSSCKKVFGSHQALGGHRASHKNVKGCFAITRPD
SGPGSGPEDLEYGELLSMNQQHKCNICLKIFSSGQALGGHKRCHLEKEEGVLPTTILSISQTFKFDLNTPANLHDDSISSTSDL
DLRLCL
>SrC2H2.t1-Mx.14
MDPAGDRRLPLLNLQNLQSRMNNVQRFISDSLNSNTIISDTQMQIISNEITSAIKDVIFNGSALISGASLISSAGFANSRSETE
TPNLSIPEPELNCNDLKDETDLGFPKTEVLDDEIGGDWDIVELDAVELLAEHLHFCEFCGKGFKRDANLRMHMRAHGNKFKTLE
ALSKPEKSVVDSESNGVRKTRFSCPFAGCSRNKLHKKFRPLKSVICVKNHFKRSHCPKMYSCNRCHKKNFSVLADLKSHLKHCG
ETKWKCSCGTSFSRKDKLFGHLALFEGHMPAAAAEDDSKAKGAVPPPPAVENVDVKGVEWADDDDMFFNGLGSIDDDFCLQDLL
VDGSIDWGL
>SrC2H2.2i-Mx.03
MEESRRIVINHQSSSDQLGAYKCRTCGKRFDTYQALGGHQGIHKKVDKTMFNLSLIPPNSSCSSGLHPCKICPEVFRTGTALGG
HMTRHRIVKDLVSVKIGERKQEVVVAADQAETPRLKAEEDDDQDCKLRQELIWAAKEWRLCI
>SrC2H2.1i-Q.20
MWNPNSVSANSHEEDDSWEVKAFAEDTRNVMGTTWPPRSYTCTFCKREFRSAQALGGHMNVHRRDRARLHHAPPNMINTNISSS
NSSTLLIPTQELVANGGLCLLYSLPKPNALFNPSSLNNYKGNPSTLLSISPHPNIDDVIDFPAAGVAPHSFNSSMSNSSYTEAS
ASAKCQSNEKVIIDKKRRRTDSAIEGIDLELRLGCSSLGF
>SrC2H2.1i-Q.21
MDQKHKPAEQCRSEASSISAEGESLKNKTMDIKPLEDGSKTVFVDQSSSRVLDLKFTSMEDNKLELNLFNPSDLEVATSSHASE
SSKEMTQEKTRVFSCNYCKREFSTSQALGGHQNAHKQERQIAKRRQMEVPPYGHLMQLPPPNYGNYTYYPSFANFTSSQLNRSS
LAIRNESYIERPSSWSTPPLNYRFAPTPRHEQFATRLTYFDRSKMLESFQGNITNNGGGFCSPMMASSSNFKSEGGMGLTHDFF
GDSSRGGNPTARVNHGIKQEISANNISDQDGSTSGLDLSLKL
>SrC2H2.1i-M.06
MHETNHTQDSSRHLHSRCTCKNVKGKHDASRAHISTNTNEKINHKHKVVVTSKSTSSFDNDTREEKRFSCSSCSRKFSTQQALA
GHRNTHRREWESILDKPDNRKINQCCKIKVPFQMHEASHLSNPITDLKRQLSSRNSSSIQRDASSTIHKPLVCTSHYSLSNPIN
TLTFGCVERCTLVKPTNTNPTNNHNFRSRRHSLANPNDTLKESSTQYSLSNRVNAFNDSLTFPSNIFPNSINTFLGPSLIANQV
SFFGLIKTPHMPSIQDPNHTLYNCAKPHGKGKQMMGGNIRRVGTNSSHVDLRSLWRSTSPISSSKSTEDGGSSKGIMTHDLFPL
TLAHDNRHHDIVHSSQGLLPKLDHNGESVDLDLKL
>SrC2H2.4i-Mx.03
MLNSDRNSVEKSDQQVMKHVCKLCNKGFPCGRSLGGHMRSHVINSTDHHQKMKLSSLNNNGGKHTEVTNGSSNSNDLGYELRKD
PKKTLKANSDDFVVLDKLCKECGKCFQSWKALFGHMKCHSDKVSKTAISNQDSWISKPDNGNSGAQLKKSKSKSKSRSRNEATK
RCLVTVTTASSSVSMNANHASTSVDSDDQETEIAMCLIMLSKDMGKWGEKMIENGCKMKRIAGFHEIKTTTIDHEEFDDQIKRN
FECSTCNKSFQSYQALGGHKASHKKLKGCFESKTDFKDEPMLDHDHMINGCYEKTSENHQSSSSFNLGNSLKNTMVVGAHECSI
CLRVFSSGQALGGHKRSHLIAEAKLNQQNPNLIEKFDKPVRGFLDLNMPPDDFIDEEQETMMMKTSSTNTGYNPWCYNHESPLL
GLLSTR
>SrC2H2.1i-M.07
MSFLNLHQPENSHTSSSSSLLLSPLPHSEHRVFSCSYCSRKFYSPQAFGGHQNAHKLERTLAKKSRELTSDSNGSTQVGRVQPP
MVDGIQYQDLSGAHEMRTNVDYGYNGESVEDGSNHLDLSLRL
>SrC2H2.1i-M.08
MSFLNLHQPENSHTSSSSSLLLSPLPHSEHRVFSCSYCSRKFYSPQAFGGHQNAHKLERTLAKKSRELTSDSNGSTQVGRVQPP
MVDGIQYQDLSGAHEMRTNVDYGYNGESVEDGSNHLDLSLRL
>SrC2H2.t1-Mx.15
MSNISGDEGCSFSSGNNNNTGAVVKDGQQRQPPIVNSTDFGSVSQELAPDSSKKKKRSLPGTPDPNAQVIALSPTSLMAKNKYV
CEICNKGFQRDQNLQLHRRGHNLPWKLRQRTNTEIIKRVYICPEPTCVHHNPARALGDLTGIKKHFSRKHGEKKWKCEKCCKKY
AVQCDWKAHSKICGTKEYKCDCGTVFSRRDSFITHRAFCDALAEENNKLTQTMQQDHHNDQSTQNINLTTSITSPEFSHGCMQD
SKTSSELLPLNIMQCGRGSLFVNSPRNASPSSLHLGGTSATALLQKAAKMGATSSNSNNNNNNHNSSMNNFVTTMAPHSYGGNG
AYHFTETLVDQYNPHQSQLAGVFGGGLLSQFQENSLPGFFNQSVNGGGNIDELNVYSGFTNPGKEAVMNNNNNNVSHDANLEFS

DSRNPLLRFKRDGNGDNLTVDFMGVGGMRLRSFNEQHHQGMEI
>SrC2H2.t2-M.01
MGEETTITGRDFPIFRDIRRYTCGYCGIVRSKKTIINAHIQSHHQDEIKETEVLEQNGTKMNACEECGASFKKPAHLKQHMQSH
LLERPFTCPIQDCNSSYRRKDHLNRHLIQHQGKIFECPIENCKSKFSIQGNMTRHVKELHHESQDNADDTKSKKQFACSEPGCE
KVFKYASKLKTHEESHVRLETIEAFCGEPGCMKYFTNEQCLKAHIQSCHQHINCEICGSKQLKKNIKRHLRTHEKVVSNEKIEC
SFDGCNLTFSTGSNLKQHIKAAHFQEKPFVCSVSGCGMRFSFKHVKDNHEKSGKHSYTVGDFVEADDDFVSRPRGGMKRKLPVM
IDTLMRKRILPLGDSENVQGSEYVSWFLSAGNEE
>SrC2H2.4i-Mx.04
MERITGDVMIGSPSSDGKMVIKLKIRQLASPEVMEGDESPTEDVIGILSSKSMEEEKRVCLECNKEFSSRKALGGHMRVHVQVQ
SANKNPNSLKPATKLRKTVHQDFNNGDRDLKVIKPYYMNSVNDEGKPTCCQCGKSFPSMKSLFGHMRCHPERVWRGILPPSNTA
TTVARRRKIDDDDGGGGSDQVVDLTKFLRGWPVTERRGRKALKTADDDEGLLEAVEDLMILAHGGASMAESGVTQRQLKAEDEI
DGKLPEKLELEDVNSTHMYANKIKRRKKMKLMMELEHTAVVTGGTTAVSPLPEQQPVTDPCKYKCNTCNKCFATHQALGGHRSS
HNKFKITSSDHHQIEEHDYQSFLMNQSCENLLKDIEFAGGFPASDSANSNSNSSVHQCKICDKVFATGQALGGHQRCHWTGIIE
PQTEAPSSQITFTGEEGSRTGIGRKVLDFDLNEDPPIMMEEDEAANVNDNGNGYASSSYNSNMG
>SrC2H2.3i-Mx.05
MKGIFLDDNMSNLTSASNEASLSSSSNRNEIGTLYPPSQIHQSSFGSVPISTNNQTQSNKKKRNLPGNPDPEAEVVALSPKSLM
ATNRFLCEICNKGFQRDQNLQLHRRGHNLPWKLKQKNKLEIVKKKVYVCPEPSCVHHDPSRALGDLTGIKKHFSRKHGEKKWKC
EKCSKRYAVQSDWKAHSKICGTREYRCDCGTLFSRRDSFITHRAFCDALAEESGRFSSSTLNHLPLHLPINFPLKTEPQLLLQN
PQLSFNVGSSTPNHNHQIPSWLNHHHQQQQEQENPNPNNLHLPSPSSHMSATALLQKAAQMGVTMSNPPPPSTATSIILNGSHQ
TLQHDHMCAPLLSPHHHHHHPSALSNLSSSDNHHVMLESSSRFANVTTSCMDQLLLHPNLGSTNIHEIFNGMFNSSKKDHNSYQ
EQVFTQTQASIEPISNTRKDGSSGNKELTRDFLGLQAFPNPNDQQFLNMNGLDHMNQMNPMNPNHNPNQIPWQG
>SrC2H2.1i-Q.22
MLNHSDQPLHSIDDQVHDDGQSGLQDERSYECTYCKHGFTNAQALGGHMNVHRKERAKNRLDYSTNNKSSTRLQESKFMLRSSP
MMNDSSLRNYENPNGLITCPSREEMRLSLSLSLRFGQSCEDEESIQGGNEDDELDLELRLGQDPWKCS
>SrC2H2.2i-Q.11
MALEALNSPTAPPTPLFRQDSFNHLHYLESWTKGKRSKRPRIDHPPTEEQYLALCLMLLARGGAPASDSTAQPANRAGSGSGSP
VDLKLVYKCSVCDKAFGSYQALGGHKASHRRNNTGGSDVEQSSAAMMTFSATTGGNGKSHECSICHRCFPTGQALGGHKRRHYE
GTVGGGQTSTGMTSSNHSQRGFDLNVPAFSENMFSGFADEEVESPHPAKRSRPFAPAKLQISVDH
>SrC2H2.2i-M.03
MSSPEKRCLYEVLGLHRDCTADEIRSAYRKLALQRHPDKLIKSGLSEAEATASFQELVNAYEVLSDVRERAWYDSHRSQILFSG
TNSNNSASGSAVVPDLFSYFSNSVYSGFSDKGKGFYKVYADVFDKIYRNELNFARTLGLGDVVKEAPLMGNLDSPYAQVNAFYG
YWLGFVTVMDFVWADQYDSMAGPNRKSRRLMEEENKKIRKKARREYIETVRGLAEFVKKRDKRVIDMQMKRNEELEKKKEEERA
KKKELEREKAEKARAYVEPDWAKVDDDDVADEVVDEEEDVRKNELYCVACGKKFKSDKQWKNHEQSKKHKDKVAELREAFGEED
QEDDDDDNNNDVGDADGFLSADEVEKLKEQFEGLELEKEEKGDDQESQSEEFVDVDNGLKEVEEEEEEDGDDDDDENSVLKAML
KNRKNVGSTRKQKKVYVEVEAEEEVDLMEYNNVKGRRKRRGKKETAKRDEDEEHERNDKPEISKKAEVDEKHDDGLQTEIPSFA
DNENNGGGDNGPTGKIKVSKQAPAIKVTNKKETNSKAKAPSKGKKQKATSINSGHECDTCGVDFDSRNKLHKHLNDTGHAAIKS
R
>SrC2H2.1i-Q.23
MAVNASNLESIELVCNKKLKLFGFLIDPCAKGSKDGESVDQKPVFASSNLKKYKCTYCCKKFLNSQALGGHQNAHKKERLRKKK
MELQAKKAKFNLYFESILDDNHDLSSSFKSFSSYFSFYYYQNMNFSNTRITNLPTLVSFSRF
>SrC2H2.t1-Mx.16
MMRQLRVMMLEDNLNSASGDLHLHANTVNSCSESKDQENGSNHHLNHQQYSDHPPPSQTQPLIKKKRSLPGNPDPEAEVMYLSP
NTLMATNRFVCEICNKGFQRDQNLQLHRRGHNLPWKLKQRNKNEIVRKKVYVCPEVNCVHHEPSRALGDLTGIKKHFCRKHGVK
KWKCDKCSKSYAVQSDWKAHSKTCGTREYKCDCGTLFSRRDSFITHRAFCDALAEESARAITGLDPTTIPGQIPTHFNDHLHLL
NPIHTSFLKKEENPFPSWLQTQTQSQDQDSSGPTLVAYHPAPSAYMSATALLQKAAQMGTSHLSKGTSIQAPCTTSLRAHHQPY
IAHMSAVNNTTCGTCGSRGLTGDGACACGPTMSTLTPPLAHHPSSSVNLHPINMSMMMMMNQDSFADVSTFEDAFGQVLDAKKE
GDDDGNGSLNVHESRMMMMHGSKSGNCGNIGVGVMGKEGMTRDFLGLEAPSHSYGDIVSSNQTLKLWQAK
>SrC2H2.3i-Mx.06
MEEIGGELKKHMCRNCNKSFPCGRSLGGHMRSHVINSTDHHHHHNHHHKYHHHDQVKNKTCLSVLDKICKECGKGFQSWKALFG
HMKCHSIKLLINKNKKIASLDQDSWARHSDNENSDSKDRSIKRSKSRSTNRRNKRYIVTTASSSISMNANCNQISSNNASTSMV

SEIEQEQEAEVAMSLMMLSRDMGRCGNEFESSNYCYKSSVLENLREVEDKDLIRNDSKMNRFAKIEVGFDFLGRSEVGIAKINE
FGSNLVKFNDSNKRKFDHDQAICGHKAGHQQPKRDFYSKIQIENKIEHKPVLDHDQTINGYVSKTSNDHQESSSFNLDVGSLKK
KMVLRSHECPICFKTFTSGQALGGHKRSHMISEAKLNQENKTIVIKKQDERVCDTRVLLDLNMLPEEEMNVSSSTTEYKSYYWN
DNDDHQSHESRILCLLSTS
>SrC2H2.1i-M.09
MSTVWFSLKKSFHCKSGSSDVHDPKSRNHLSPILTRRPGRCGCSKSIANLKDVINGGSKRHSEKLVNCSPRSIGSSEFLNPITH
EVILDNSTCELKITSFSGGFHDGFALLPETPTSGGVPTMQQFKNTPPKRKMESLDGNGIGNSGHLGIFGKSMNNTVLQKARRSS
EKDSGGCTGGGVTCHKCGKQFRNLENLEAHHLSKHAVTELTEGDSSRKIVEIICRSGWLKSENSSSRIEKILKVHNMQKTIARF
EEYREFVKTKASKLPKKHPRCLADGNELLRFYGATIACSLGINGTSSLCDFNKCCVCQIIRDGFSTKKELNGGIGVFTSSTSVK
AFQSIEVFEEEPNIRKALIVCRVIAGRVHRPLENIQEITNQTGFDSLAGKVGLYSNIEELYLLSPRALLPCFVVIGKP
>SrC2H2.3i-Mx.07
MACSDDQQPNFKHFCKVCKKGFMCGRALGGHMRAHGVGDEIDNLEDGDDPSTDWVSSPETDDDEDGYGHYGGGGSGWCKRKRSS
RICFSENEEDITLAKCLMALSNSRVDFMETESDAQKPNNIFSPVFLSNFNKPPPPLDKAKGVVTTTTTPKGMFECKACKKVFSS
HQALGGHRASHKNVKGCFATRNDHFDENLTDEDMHGDIKHSNTSRWDHEPITGPVPLAGRKSKVHKCSICSRVFASGQALGGHK
RCHWLTSNYTSDNYVGKLSFHEHIDQLHKRALAIPKELDLNLNLPVSGNNTIASKAPMNAKEDHKNQSNKNNGDQDDHVATTTM
ESVDGEGESKMKLAKLSELKDMNNTNEGSSSWLQVGIGSVANVAPDP
>SrC2H2.3i-Mx.08
MACSDDQQPNFKHFCKVCKKGFMCGRALGGHMRAHGVGDEIDNLEDGDDPSTDWVSSPETDDDEDGYGHYGGGGSGWCKRKRSS
RICFSENEEDITLAKCLMALSNSRVDFMETESDAQKPNNIFSPVFLSNFNKPPPPLDKAKGVVTTTTTPKGMFECKACKKVFSS
HQALGGHRASHKNVKGCFATRNDHFDENLTDEDMHGDIKHSNTSRWDHEPITGPVPLAERKSKVHKCSICSRVFASGQALGGHK
RCHWLTSNYTSDNYVGKLSFHEHIDQLHKRALAIPKELDLNLNLPVSGNNTIASKAPMNAKEDHKNQSNKNNGDQDDHVATTTM
ESVDGEGESKMKLAKLSELKDMNNTNEGSSSWLQVGIGSVANVAPDP
>SrC2H2.1i-M.10
MPRLTMVNLVRFCVSDRRLDFSTTQTLTALVRLIRHYHFNTNQTLKTPDQFDSVHNTSRNIVAVFWDLDNKPPRSVSPFDAAIR
LKKAAESFGVVRYKIAYANQHSFDYVPPEIREHRRDRKTLNQLENKGVVKPADPYICRVCGRKFYTNEKLINHFKQIHESEHKK
RVSQIESARGSQRVKLVGKYSMKMEKYNNAARDILTPKVGYGLGDELKRAGFWVSVVSNKPQAADVALKNHMVDMMDRCQMECL
ILVSDDSDFVEVFKEARLRCLKTVVVGDSNDGALKRVSDAAFSWQEIIIGKAKKEAVSVVGRWKDSKILKRLEWMYDYERERKL
YGSCSEDDQNDDLDVGNLVSDEVKTDQSRAWWKLESDSDVAASS
>SrC2H2.1i-M.11
MPRLTMVNLVRFCVSDRRLDFSTTQTLTALVRLIRHYHFNTNQTLKTPDQFDSVHNTSRNIVAVFWDLDNKPPRSVSPFDAAIR
LKKAAESFGVVRYKIAYANQHSFDYVPPEIREHRRDRKTLNQLENKGVVKPADPYICRVCGRKFYTNEKLINHFKQIHESEHKK
RVSQIESARGSQRVKLVGKYSMKMEKYNNAARDILTPKVGYGLGDELKRAGYWVSVVSNKPQAADVALKNHMVDMMDRCQMECL
ILVSDDSDFVEVLREARLRCLKTVVVGDSNDGVLKRVSDASFSWQEIIMGKAKKEAVSVIGRWKDGDILKRLEWTYNYERERKL
YGSCSEDDDDDQNDDLDVGNLVSDEVKTDQSRAWWKLESDSDVAASS
>SrC2H2.1i-M.12
MVNLVRFCIYNGRLDLSTPQTLTALVRLIRHYHFNTNQTPKTPDQSASVHNPTRNIVAVFWDLDNKPPRSVSPFDAAIRLKKAA
ESFGVVRYKIAYANQHSFDYVRPVIREHRRDRKTLNQLENKGIVKPADPYICRVCGRKFYTNEKLINHFKQIHESEHKKRVSQI
ESARGSQRVKLVGKYSMKMEKYNNAARDILTPKVGYGLGDELIRAGFWVSVVSNKPQAADVALKNHMVDMMDRCQMECLILVSD
DYDFVEVFKEARLRCLKTVVVGDNNDGALKRVSDAAFSWQEIIIGKAKKEAVSVVGRWKDSDILKRLEWTYNYERERKLYGSCA
EDDQNDDLDVGKLFSDEVKTDRSRAWWKLESDTDVATSS
>SrC2H2.t1-Mx.17
MKGIFVDDNMSNLTSASNEASLSSSSNRNEIGTMYPPPQMQQSFGSVSITTNNQTQSNKKKRNLPGNPDPEAEVIALSPKSLMA
TNRFMCEICNKGFQRDQNLQLHRRGHNLPWKLKQKSKLEVVRKKVYVCPEPSCVHHEPSRALGDLTGIKKHFSRKHGEKKWKCE
KCSKRYAVQSDWKAHSKICGTREYRCDCGTLFSRRDSFITHRAFCDALAEETVRSSSSTLNHQLPLHLQMDFPLKTEPQHLLQQ
PQLSFNIGSSSTPHHNHQLPSWLDHHHQQEQQQQQKNPNPNNLHLHSPAHMSATALLQKAAQMGATMSNPAPSHDPHSNASIII
NGSHQTLQQDHMCAPLLSSSHHHHHHPSGLSNLSASDNHHHVMLEASNCMDQLIHPNSLTSTTVGSTNIHEILNNLLSSKKDIN
GYQDQVFSQSQATTQTNLSTLKDGNANDELTRDFLGLRGFPNPNDQHLLNMGHMAGFDHMSQLNPNHNQNQVQWQG
>SrC2H2.4i-Mx.05
MDWDDDRDKMIGSPSSEGKMVIKLKIPKLVPAVVEGERPPEEMIEISSNSKPANEGKKICLECGKEFSSGKALGGHMRVHVQSA
NRNPNFQKSPRTNKVKKSKTAHQDHNNGGDVHSKPYYMNCVNDEGKPTCSQCGKTFPSMKSLFGHMRCHPERVWRGILPPPNTS

TSGGDKIQNQNFGSGSSYSSENELSMSEDGGNQVVDLTKFLRGWQVTDRRGRRAALKAVDDNEGLLEAVEDLLSLANGGGSMAE
SGVTQPRLKVEVIEGSNSNSLSNKEHEIDPNSPSINLGVTGKGKVVMEEELEVKLPAKLEVEDGDMNHMIKIFSDHEFDCRNNT
NEQFLINYKYKNNHNKIKKSKKMKLMMDLEPNDIITITNRGPTTIQSKQQPVVTDCKYKCTTCNKCFTSHQALGGHRSSHNQPK
ITSTDHETDYKSFLMNRYGENSDKEVAFEGGFMVALTSGGANNSISTSNIHQCKICDKIFATGQALGGHQRCHWTGPPSSQITS
TEEAASQTGDRKVLDFDLNEVPPVMMEEDEGGNNGYASSSYNSNIMC
>SrC2H2.t1-Mx.18
MSNISGDEGGSLSSGNHGGVIEQDQERLQPIVNSHQEVVTDSSKKKKRALPGTPDPNAQVIALSPTSLMAKNKYVCEICNKGFQ
RDQNLQLHRRGHNLPWKLRQRTSTEIIKRVYICPEPTCVHHNPARALGDLTGIKKHFSRKHGEKKWKCEKCSKKYAVQCDWKAH
SKICGTKEYKCDCGTIFSRRDSFITHRAFCDALAEENNKLTQTIKHDHNIHDHHPTQINTIKTSITSPEFSHGYTQGGRGNLFV
GSPRNTSSSSLQLGGTTLSSPLTSATALLQKAAQMGATSSNGNNNNSSNNNSMNNIVTTMAPPSYGVGGAYHGTETLVDVYPPH
QSQLSMIIGQGFSSQFQESSLSSFFNPSIIGGGDNDGTEAYWSMNHNSKEVNMENNDNNVGHNANSGNKNTLLTFKRDGNGDNM
TVDFMGVGGMGLMSFNEQHHHGMEI
>SrC2H2.t1-Mx.19
MMSGDGFSSPPSSLTSFINDPISNPNPNSSISGKRKRNQAGNPDPEAEVIALSPASLMATNRFVCEICNKGFQRDQNLQLHRRG
HNLPWKLKQRNKNEVIKKKVYICPEKTCVHHDPSRALGDLTGVKKHFSRKHGEKKWKCEKCSKKYAVQSDWKAHSKICGTREYK
CDCGTLFSRKDSFITHRAFCDALMEENSRMASLPMVPNMGNLAFRNELMMMNRGSGGGSGGGGGGDGQFQAMFGGALESNLDVN
GTKPRLPIWLDHNDNTPNLENPNNSTFFGSSSSHYNNGGMLPPEMVNLLGRYNGLPHGMSLKDEEQAQKEMHLNSMYNFNADST
SQNSLMCSTLPPVSPSPVGNMSATALLQKAAQMGSTRSTNSGIAVGNDTGFGLMSTSLSNLSAASPARPPPSKGPAGGEYGDGD
LTRDFLGVARNERSVSFNLQQELNKYASSSMGFNQFNRNHE
>SrC2H2.1i-M.13
MVTNGSNIAKGPNPNPPPPPPPPPPPPPPSAGTKRPMTPGKVKAKTVICPICNRNMYHEKALNGHIRWHTAEEREAAGLGIARS
LASTVVVEEQDTAKRFKVPDLNRSPPPEDGEK
>SrC2H2.1i-Q.24
MEKLNSQPHELMNIEFSSQLPYTPPIRLFGKQLGGSDPTIIITNDSSSTVTAAVAVATTTVTATVTTTATTIRHRTKQNIETQR
TFKCHYCSRNFQTSQALGGHQNAHRKERLHAKRTYIQSVTIHHSYPNHHNRPLTTATSSPYHHTGTGSIINYNTSFNFKSTSNQ
TPIYGRPLAVSRFPKSNGGGLLNYSNAGSSSRRLHMHESKTSFNDQVSLDLHL
>SrC2H2.1i-M.14
MEFWGVEVKSGQPLDVVLDEHKVLHLSQACLGEIKNNKSNESVCLHINIHNKKLVLGTLNSERLPQQIFDLVIHKGFQLSHNWK
NGSVYFYGYLAEQPYYGSSESEESDDDELEEFPVPNPIPNGKDIKKEDNPVAVKKAESSLAAANKKDGKQKVTIVEPEKDAKAD
EDDSESDGSDSMSEDSDSDEDDEDSSDSEEGSDEDEETPNKQQSGKKRPNESAMKTPVNEKKAKVSTPQKTDGKKAGGHVATPH
PSKPNKQLKSENKSDQKTTASASTDGVHSCKPCNRNFKSDSALQSHNQAKHK
>SrC2H2.t1-Mx.20
MTNIEGVQWAIDKGCECNHTISTRFINIFYSFAGYKYLHHSFISSSSSPLFIPSFPKTTRENSIKNKCAACYRQFNKKEHLVEH
MRTSYHSVHEPICGICGKRSRSFESLREHLIGPLPKAECERVFRELGCDICLTILSSRNALRAHRDACKLSYGNGLLYRFSNLG
IKDDLRIDNGKTRAVALACKMVGGGSDGSLDLCAKVCITDEYENILFQSYVKPYLPVTNYRYEITGIRPEYLRDAIPLRQVQRK
IQDFLCNGEPIWKIRPRGGRARILVGHGLDYILKCFELEYPAIMIRDIANYPPLMKTSKLSNSLKYLTKAYLGYDIQTGIQDPY
EDCIATMRLYRRMRSQNHRNENYPLATDPQNKNNFASWRQSELERMSPEELLAISRSDFYCWCLDNKDYV
>SrC2H2.t1-M.01
MTMTDNHLQTQLNSEFQYWFPVLRRFPPDDPFFASGNIERELLAKQVALDLTEEEKQQLRNLDDEDSEVTCPIVGCGAHLRSLA
EFEDHYNARHTASCSVCCRVYPTSRLLGIHVSEAHDSFFQAKVARGYPMYECLVEGCGVKLKSYKSRHQHLIDKHNFPSSFEFF
KKAQPSKKERFKKKQQKQRDARFTTENEEESSAMQVEEETLNGLVYAVSKLSTSDSTPSSISFGRRNTRGLTFVPRVVQRETKK
>SrC2H2.t1-M.02
MTMTDNHLQTQLNSEFQYWFPVLRRFPPDDPFFASGNIERELLAKQVALDLTEEEKQQLRNLDDEDSEVTCPIVGCGAHLRSLA
EFEDHYNARHTASCSVCCRVYPTSRLLGIHVSEAHDSFFQAKVARGYPMYECLVEGCGVKLKSYKSRHQHLIDKHNFPSSFEFF
KKAQPSKKERFKKKQQKQRDARFTTENEEESSAMQVEEETLNGLVYAVSKLSTSDSTPSSISFGRRNTRGLTFVPRVVQRETKK
>SrC2H2.2i-Q.12
MDFMPESTAVTTFDRAQHIITRKPTDHRSMVESSSSSYYSGGDGGCMLSYHQSPAMSSEEEEEEDMANCLIMLAQSVSHAKIDK
PDSDIFCQKTKKLKVRSVTEMAAITGGENSGYQSYYECKTCNRTFSSFQALGGHRASHKKPKPTVDDKNSDEDQLQRLVVDEEN
KTITNKLSSSPVTHDYMQTVYKNSKSTKVHECSICGSEFLSGQALGGHMRRHRTAPALVSPIKAKLSPESKKMNEKSPVLSLDL
NLPPPEFVFSASALVDCYY

>SrC2H2.t2-Mx.05
MSSNHPYSTYFNGSWFNLNPNFNPNPNYHCLYPPISYSNFTHYYDYQSSPPSPPLREALPLLSLSPTRKPSNQDIDFTNSTTND
ASSTTTMEVDDHKNKVAAGAAAADDDDHHHHDVHDDHKNVTVALHLGLPNASFSEADLISKLSTNSDMNVTEADDHYKHVEEEE
GTNSNAYLASALNKGQYWIPTPAQILIGPTQFSCPLCFKTFNRYNNMQMHMWGHGSQYRRGPESLRGTQPTAMLRLPCYCCAPG
CRNNIDHPRAKPLKDFRTLQTHYKRKHGIKPFMCRKCGKAFAVRGDWRTHEKNCGKLWYCSCGSDFKHKRSLKDHIKAFGNGHA
AYGIDDCCFELEDEEAASEIEQDNESSHCDN
>SrC2H2.t1-Mx.21
MMSGEMFPIPLQDRHHQPPSHQPNPNSKPISNLKKKRNLPGTPDPDAEVMALSPKSLMATNRFVCEICNKGFQRDQNLQLHRRG
HNLPWKLKQRNNKEPIKKKVYICPEKTCVHHDPSRALGDLTGIKKHFSRKHGEKKWKCEKCSKKYAVQSDWKAHSKTCGTREYK
CDCGTLFSRKDSFITHRAFCDALAEESVRISSVTPNNLGLKNEGMNESVMNPNFLHGFSGMTQFGSGFRGDFGDQNKPGLSLWL
NQANSQLNLSPMDQMTMNSNPYMSSSSSSLADMVNAYGSSSMANFTNTNQVPEDLKEETLASLYSMNQPHQTESTGPMSATALL
QKAAQMGSTRSNPSAFGTSFGLMNSSTTSNTAVSLVKNNITTQVQVSSAMNNNSSKSSPRVSNVDQQQMIMSRNETMAPLMMDR
NFSGVDNGLTRDFLGMGGEGGRPLLPQDLVNYASFGSTMGSMRQFASN
>SrC2H2.t2-Mx.06
MRDVEVPNWLKELPLAPVFYPTDTEFADPIAYISKIEKEASAFGICKVIPPLPKPSKKYVINNLNKSLLKSPELGSDVKLNNVV
DSGLTRAVFTTRHQELGLNNSNKRAKDGLSGSGQPSPPVVNKQVWQSGEIYTLDQFESKSKTFARNQLGINKDVSPLVAESLFW
KSACEKPIYIEYANDVPGSGFGEPVGPSRFLRKHRRRRRMFNRYNKDCSDISEQPDNKTVKTNNCDNLKGEPVGPSSTSSSVDM
LEKDDGSPDGDYDIEGTSGWKLSNCPWNLQVIARSPGSLTRFMPDDIPGVTSPMVYIGMLFSWFAWHVEDHELHSLNFLHIGSP
KTWYAVPGDYAFTFEEVIRSKAYGGDIGRLAALTLLGEKTTLLSPEIVVASGIPCCRLVQNPGEFVVTFPRAYHIGFSHGFNCG
EAANFGTPQWLSVAKEAAVRRAAMNFLPMLSHQQLLYLLTMSFIPRVPRSLLPGIRTSRLKDRQKEKRELLVKKEFIDDLLKEN
KLLNNILQKNPSYHAVLWDLETLSPSVIKESVVTNNENVQIQKEDYLDPETLADIEDDDMSSDFQIDSGTLPCVACGVLGYPFM
SVIQPSTKAVIENITVTGHGFVQPHAIETESVKVDKNWNMSNVYLRPRIFCLEHACKIEELLDSMGGAKLLIICHSDFQKIKVQ
ASTIAEQIGSAFRYNEVQLNDATQDDLDLINFAIDNEQKEDESVKEDWTVKLNVNLRHSVKLRPKLSTDKIHHALSLTMDALFA
DTTRASSVGASATVLKWEATKFRSKRKSNWSVKKLSNSEKDVEFMEKLSEPQMIKKEKVLIHYSRRKLKSKPQDLVTDFSKNTN
DVGFLVSSDLSTVGQSDIAAIIENDGIEKDDINNETCMQNENSSVVTKNGSELTKMGGSSTDECDTDSNDGVKKNDDNTLESNS
TGGNKRKRELELLQTNENSAFGGFIKSPCEGLRPRGCKDLHKVGIFINKKPIPEKPTKKSRNPNVTERPSAQPNGSNARNPEKT
DHRAHRCNHDGCKMSFKTKTEVILHRKNRCPHEGCGKKFSSHRYAVLHFRVHEDSRPLKCSWKGCKMTFKWAWARTEHLRVHTG
ERPYKCKVEGCGLTFRLFLILADTEGKPGIS
>SrC2H2.2i-Q.13
MVCLSMKRSREDEYDTTITNMANYLMLLSRGTTSTEPYGADSVSRVFECKTCNRQFTSFQALGGHRASHKKSRLVDYDMTGHRD
ESLQSKPKSHKCSICGLEFAIGQALGGHMRRHRAALTADDQSSPQIELTTPVVKKVNSRRIFSLDLNLTPFENDMGFRVDDEKL
TPITVDFFL
>SrC2H2.3i-Mx.09
MNNDVIMGGGATSSTSSDFQLCPSSPESQQIFQPTSHFQITTMNPQQQNAPKITRSCSECGKKFWSWKALYGHMRCHPERPWRG
INPPVVAVEHSGSTSTSTNEERYVASCLLMLANGPTRFEKYEEFGNHHGWFECTSCKKVFGSHQALGGHRASHKNVKGCFAITQ
KEGGEEVDEGEIEGHFEYNISSEHKCGICSRVFSSGQALGGHKRCHWEKEDDVSLAALTSVPSHGRYRFDLNSTAPREDYPSYS
IGLGLRLGL
>SrC2H2.4i-Mx.10
MDDQNTNFKHFCRICKKGFMCGRALGGHMRAHGIGDDTGILDDEDPASDWEDKQGNKRMYALRTNPNRLKSCRVCENCGKEFLS
WKSFLEHKKCSSDDGESLVSSPESEADEDDGYDDENHGARRDCQSSGWSKRKRSLRAKVGSFNSNCPSSEDEDLVLAKCLMELS
NGRVDPAETDLEDSCTSPSREEQRQNPMVPMTTLSPPFARAPPPLDYKAKGVATTPKGMFACKACKKVFTSHQALGGHRASHKK
VKGCFAARNDQSDDNVGDDDVITHDELYPSPKPISSYQFNQGPSTGPSVGLARQISKVHKCSICNRIFASGQALGGHKRCHWLT
SNMSDTSSIAKFNFHEHIEQLHRRALALPSRILDKSKALDLNQPALEQAGTGLRKDPRYPLSFEVSTDINLHSWNVDHKIVTDG
EVKDQKRTGDYQDHDQKASNNNEKEAVLGAIMEDDEADSKLKLAKLSELKDMSNISGSSSSWLQVGIGSTTDVGSSHDP
>SrC2H2.t1-M.03
MGKKKKRVSSDVWCYYCDREFDDEKILVQHQKAKHFKCHVCHKKLSTAGGMAIHVLQVHKETVSKVPNAKDGRESTEIEIYGMQ
GIPPDVLAAHYGEEDNDNPSKVGKLQIPSSQVGLLPGSLAYTQSTMQSIYNPGLAVPRAGWPVPPRPQTWYAPPPVGSVPPTAP
LGMVQPPLFPVQTMRPPTAPLGLQTSFPIPPPVSQPLFPVVSTNGIPSAPTSIPLNSPSDLNHPTDAHNINMPRAYLTPGFQGG
VTSSHSYASGPNTGGPSIGPPPVIANKAPAIQPPTNEVYLVWDDEAMSMEERRMSLPKYQVHDETSQVSSTLYHTHSFNTPHAT
VLNIFFINVMVFK

>SrC2H2.1i-Q.25
MADQQLYDFMKTHSHPYPHPDAAAGKHPNQPSSRLFSCLYCPRTFYTSQALGGHQNAHKRERAAARRSYITAAADNYLTETTDG
NTPITAAPRSYVSSAGVDSYLSPYSDINTPVTGAYNWSYDRQFQDGASASFVFYVAQPPPAENSDVDLTLRL
>SrC2H2.t1-Mx.22
MRSRRSFRHRNKCVACYRQFNKKEHLVEHMRISYHSLHEPMCGICGKRCRSFESLRDHLIGPLPKAECERVFRDRGCTFCLNIL
SSPNALRFHQDKCQLSRGNNKTRVVALACKMVGGGSDGSLDLIARICIIDEYENILFHSYVKPQLPVTNYRYESTGIRPEYLRD
AMPLRHVQRKIQDFLCNGEPIWKIRPRGGKARILIGHGLDHDMRCLELEYPLVKIRDTSKYPPLMKTSKLSNSLKYLTKSYLGY
DIQNGIQDPYDDCVATMRLYKRMRYQTHRIEDYPLANELERMSPDDLLAFHRSDYYCWCLDSKDVA
>SrC2H2.t2-M.02
MQVLPCNIMHYAGESNSPNQASEKAFVYDEGANNVKPEIVEGGNTNLDNVETSDDEQFGEHDEGHPNNGPLLELDVSYNTRDSG
VDSLDGDTVGRELPAGNQECESSRSEPEWLEQDQPMAVWVKWRGKWQAGIRCARSDWPLSTVRAKPTHDRKQYLVIFFPRKRNY
SWADVLLLRPINEHPEPIAYRSHNVAVKVVKDLTVARRYIMQKIAVSMINTIEQLNSEALNEDARSVIVWKEFALESSRCKDYS
DLGNMLIKLEKMILQCFIDSYWLENSLETWVQRCQTAHGAESIEMLKEEFNDAIKWNEIHTLSNSSVNPEVGTEWKTVKPEVMK
WFSMSNPSFNTENTEQQNNDGFHNTSPQVSRKRAKLEIRRAEVAPPQLETGSRSLNGPIGPTSGDVSLLGTTEPDRWGEIVVET
GNNNQETRFRSLETNSLNKNKQCTAFIEAKGRRCVRWANDGDVYCCVHLSSRFSATLVKPEVNTPPNDALLCDGITVLGTKCKH
RALPGSSSCKKHRSNKDILVISPSPPENKLKRKLEDESRGDSEASNCKEIVLSGYFGTQVADQAQIEYNGPGTGPGTGPGFGLG
PMNCVGDDVVCNEAPTRHTLYCDKHLPNWLKRARNGKSRIVSKDVFVDLLKSCQSHEQKLHLHHACELFYKFFKSVLSLRSPVP
KEIQLQWVISEASKDSKTGHFLMKLVCSEKERLMRIWGFDNDNIAQYVEPVNVVRPNYNDNDDHGNNNYDNIVIKCNICSLEFV
DDQTLAKHWIDNHKSEAQSVFKRYVCAICLDSFTKSNLLEAHVQERHNVEFVEQCMLYQCIPCGNRFGSPDQLWAHVGSQHPAH
FKQLQNNVVIPPNGEGYNGNNGNGVIQVQNVNNSNQLGSKRYICRFCGLRFDLLPDLGRHHQAAHMGLNPGGSRKRGAGSTFLA
NKLKPGRRFKKGLGMRNRGLVTIKKHNQEPFNQVPEGVYQSSESITLGRLPESACSDVAKLLYSKISKTKPHPGIIELLAIARS
SCCKTSFETSLANKYENLPERIYLKAAKLCSERNIVIEWHQEGYICPKGCKLITDDSHDLPPLKNLPESSLRPDTETSAPPEWP
MDECHYIVNFSNPRLESNERSGIVLCDDISFGKESVPIACVVDEHLLGSLHDDDGQSNSCFLPWESFTYVTKSLIDKSVNLGSQ
SLQLGCACAHSTCSPKGCDHVYLFDNDYEDAKDINGKSMKGRFPYDDKGRIILEEGYLVYECNAYCSCNKYCPNRVLQNGVKVK
LEVFKTEGKGWAVRAGQPIARGTFVCEYIGEVLDKIEANKRHNRLGKEDCSFIYEIDARVNDMIRLIEGEATYAIDATKYGNVS
RYINHSCSPNLENHQVLIESLDSELSHIGLYASRDISAGEELSFDYMYKAPPGEGRNCKCGAVNCRGNVR
>SrC2H2.t1-Mx.23
MVDFENSSAMADSAASVEGTECSSVGNETGPVPEIQPMKKKRNLPGMPDPDADVIALSPTALLATNRFVCEICNKGFQRDQNLQ
LHRRGHNLPWKLKQRDGKEIRKRVYVCPEKTCIHHDPSRALGDLTGIKKHFSRKHGEKKWKCERCSKKYAVQSDWKAHMKTCGT
KEYRCDCGTLFSRRDSFITHRAFCDALAQESARSQPNFPNINSDNHTHLQEADKSDAAVISPSCSPPPPPLTPSTGVLSPVQSV
LSSELPETSMGILRHQKSTVMETEAMETCMASATTATTTTTVSGGSTTSGNHNVTNTTGVFASIFASSQSSPASYSNLICGLTG
ADRNNITEPMSLSLSSSLYRTTTTTSSLFPPPDHHQPHHQHHRQYAHAPQPALSATALLQKAAQMGATSSNTSFLHALGLTPPQ
PPSSSVDHYQEPNIGHWNNIIQTKQERTDNSAVGPGVSANGRDYQLLMGPPAPTTVDFLGVGTGSSSGYSAYLNSIGGSRINVA
AAAGVRFDGLNDNDWDDSGDKKPTLL
>SrC2H2.4i-Mx.06
MSNLIQETGQGSMNHICVLCDRSFPSGRSLGGHMRSHVINSTDRHNHDDDDHHQMKKKKKQMKKLSSVNNGDSNSSDFCYELRK
DPKKTPKVKDSSELENNSNCLVVLDKLCKECGKGFQSWKALFGHMKCHSEKVSNNHTKHNLNQDSLISHSENENSGTELHPIKK
SRNKKMKRYIVTSTTITTTTTTITTASSSISMIVNNHASTSIKSEIEQDQESEIALCLMMLSRDERKWGNEIESSDHCNSSAFE
RLTKVEGKKPTGNGSKIKKIAENQISADYLGRSELGFTRLEKIVNQNDKFDEFQDSSKRKFECVTCNKSFHSYQALGGHKASHK
KLKESLDSKLENENKMENKPVFDHDHIINGFAAKASQNDQTTKRFNLGVGSLKKSVVLGVHECPICLKIFSSGQALGGHKRAHM
IADAKLNQQTSMNLIEKVNDSVHETRGLIDLNMLPHDTMEEDMIMNSSITNHESTTLLSLLSTS
>SrC2H2.1i-M.16
MEAISSNIESLDIVSDKKLKLFGFLIDPCANVSKNGESIEEKGVFLSSNRKKYVCQFCCKKFVNSHALGGHQNAHKKERLKKKN
MKLQAKKDKFNLYFGSLHAPNFLSFDSFSSHFSFYHSDDYQNINFSNTHIFNLPHLTCCSKFQQDDVTFLIS
>SrC2H2.t1-Mx.24
MMKKQNVILEDHLSSSATDLHTTTIAINSCSEKVDQENENGSIYYPNHQPYFAHPPPSQSQPLTKKKRSLPGNPDPEAEVISLS
PKTLMATNRFVCEMCNKGFQRDQNLQLHRRGHNLPWKLKQRNKNEVVRKKVYVCPEVNCVHHEASRALGDLTGIKKHFCRKHGE
KKWKCDKCSKRYAVQSDWKAHSKTCGTREYKCDCGTLFSRRDSFITHRAFCDALAEESARSITGLDPTTIPVQIPAHFSDHLLN
PIDHHTSFLKKEENPFPSWLQTQSQDQDCSPNTCHGVSGPTLISYNPPPSAYMSATALLQKAAQMGTSHLSRGTSLQAPCTTSF
RPHHQQYIAHVSAAAGNNTCGTSGSSGLLEDGACAGGPSTPSPPHHPSSFLHPVNMSMMTMMMMGQDSFADVSTFDDAFSQILD

TKKEDDDDHGNHLHEGHMMMHDSKSGNIVMGNVGMTRDFLGLKAPSHEHGDHINVSIDQTLKPWNVK
>SrC2H2.4i-Mx.07
MNEDNDELKFVCKLCDKRFPSGNSLGGHMRSHVIAASAAANSAESYHKFEFKKKHYDSIINGNYNGNLNSNSNSSLYGLKENPK
KSKRVSSSSTLPFTNDQICKQCGKGFQSLKALCGHMAFHSEKYKSLKYHDYSCTSKNLDHEDVSIENFDPVCVTRSKSKRCKKV
VIKPTSFCLNNNNSNNRNYKYESSSVSENDEFEQEEVAMCLMMLSKDSANWAGVHSVLESSNNNSVVFETKLSSIDMKNKNDTK
RFPIENLYSRYCRNGIKKLESGISVEGILWNGMKGFNYKDLSYEEKQEIRRNLFREFGYDNSLDKRIQDDNDDDGSYKRNKYEC
LNCNKTFNSFQGLGGHRPCHKKNNAFNSSGDYSDRVFARDQKAKCEKKTRPKKNKAHKCPICFRMFKSGQALGGHKRSHFISGT
EDKIDHIAVIEHAVATFTNMIDLNLPAPEED
>SrC2H2.1i-Q.26
MRNPLDLNNLPDDHFIRDYSKQSLDDSSSTASGIYRKKKNSSKDERWKVYECRFCSHKFRKSQALGGHMNRHRQERETETLNHA
RQLVFGTDNMVAPPHHQLGGQPAVHGDFHYAASLNMGATAYPTRLISGGSTTILPPPRPHHLIKTSMPSRLTDAYLSQYSTSRL
INDNFVDWYVSSNPQFSIQNLTNT
>SrC2H2.t1-Mx.25
MLDNHHHHHHFTIASGPEQGPSSSPDLFPSSDNGIPNKRKRRPAGTPDPDAEVVSLSPKTLLESDQYVCEICNQGFQRDQNLQM
HRRRHKVPWKLLKRDSPEIKKRVFVCPEPSCLHHDPCHALGDLVGIKKHFRRKHSNNKQYVCEKCSKGYAVQSDYKAHLKTCGT
RGHSCDCGRVFSRVESFIEHQDACMVRRIHADLPPFQQACSSRTASSTSPSNDMNFNSVQTMSRLPHSRTLFSQTNVDIENNLE
LQLLPSLSLYDQTKNHQTHLNLSIGNRNSLQNDEELKMATANNAYAEDARQQAKRQIEIAEMEFENAKRIRQQAQVELERAMVL
REQATKKIDSIMLEITCYSCRNRFEAARNNVAVMTADEASMAPSYMSSALTEGEGYY
>SrC2H2.2i-Q.14
MFLQMKRSRESELDATLTNMANCLMLLSKGPSGNEPYDNTLGRVFKCKTCNRQFASFQALGGHRASHKRPRLNSDGDLTKGTNL
VPAKPKSHECSICGLEFSVGQALGGHMRRHRTTTTNENQSFVHVESSKSVVKKVNSRRVFSIDLNLTPTENDLEFQLGISVMKL
>SrC2H2.2i-Q.15
MFLQMKRSRESELDATLTNMANCLMLLSKGPSGNEPYDNTLGRVFKCKTCNRQFASFQALGGHRASHKRPRLNSDGDLTNGTNL
VPAKPKSHECSICGLEFSVGQALGGHMRRHRTTTTNENQSFVHVESSKSVVKKVNSRRVFSIDLNLTPTENDLEFQLGISVMKL
>SrC2H2.2i-Q.16
MMFLQMKRSRESELDATLTKMANCLMLLSKGLSSNEPYDNTLGRVFECKTCNRQFPSFQALGGHRASHKRPRLNSDGDLTNGTN
LVPAKPKSHECSICGLEFSVGQALGGHMRRHRTTTTNENQSFVHVESSKSVVKKVNSRRVFSIDLNLTPTENDLEFQLGISVMK
L
>SrC2H2.2i-Q.17
MEESSSSPRNQNPSLSSSSSVMKLFGVAVRDGGIGPVTSQPDGDNKRFECQYCRRDFANSQALGGHQNAHKKERQRLKRVHFMN
HHPRRFAAPVSIFNAHAARSGEFQQPQMAVVDHYMCPQSQHQPQVLSGVPLRFPSRLYIGRQPQMNVVVQNSLRRNEQVDENND
DDDDGDGDGVDVDLHL
>SrC2H2.1i-Q.27
MEKTDKETHDFMNVESFSQLPFIRPSSLKEKGIRLFGKEFGGGGGLDSATMITDEYDTNNTASGIEESKETGENSRKFECHYCC
RNFPTSQALGGHQNAHKRERQHAKRAHLQSTMVNGSFSEAQMYGLMNYQYRFTNTPPPYYHHTNTNSTITNYNHRFYNNNSSYT
SHQTPINGNPLALWRYPNAHNSVFNRDNSINMLPVISANESRLQGSRIPTSSNYMYDSKPGVQDQVSLDLHL
>SrC2H2.3i-M.01
MCFEASPFFEYWLNPSSSTTPSSSNDTMMFSKEQDFPDESKQCLPLLNRLLDRDENKSVLNIKEEDNIEKVTVTLHIGLPSYHT
EIAGDLVVTNKLFDEFKEEDDQKKSDMKMKFYNEYNNGYNNVNSDDEQTRFWIPTASEILVGPMQFVCSLCNKTFNRYNNMQMH
MWGHGSEYRKGPKLLKGTQPTTMLRLPCYCCAQGCKNNINHPRAKPLKDFRTLQTHYKRKHGAKPFTCRKYCTKKFAVKGDWRT
HEKNCGKLWYCTCGSDFKHKRSLKDHIRAFGNGHSPHPCLEGFDHDDKECVTGSEDEIMR
>SrC2H2.2i-Q.18
MEMEFMAEFTEGMSRDRALQIIKRKRSKRQRPCSPADGDCLIDNFTPVMSSEMSSEMSTEEDEDMANCLIMLAQSVSPVTEEEK
SKSDQIRRKTEKLSSRRLTGDYVYECKTCNRTFPSFQALGGHRASHKKPKVNVEEKKPNLFNVAPPVMQNIEENYDQQQVVEED
EENKASMINNIQTGIVINNNTKVKVHECSICGSEFLSGQALGGHMRKHRPIPPTVNRTVISMTTDDSSGDTDRIIDKSPAGSTS
MLSLDLNFPPPEVVEDHHRSKFPFTGGDLPQRMVFSAPAFVDCHY
>SrC2H2.t1-Mx.26
MSGEDLLSSPSSLSAFIQDPISNPNPNSSNSSKRKRNLPGNPDPDAEVIALSPRSLMATNQFVCEICHKGFQRDQNLQLHRRGH
NLPWKLKQRNKLEVVKKKVYICPEIGCVHHDPSRALGDLTGVKKHFSRKHGEKKWKCEKCSKKYAVQSDWKAHSKICGTREYKC
DCGTLFSRKNSRMASFPIMSSGGNLNFRQDLLMMNGGGVGGGGGGGLRFPGMFGGGMLEVNLDSNPEKPRLPIWLEHNGNESHL

ENPSSSSFLGSSSSNNNNNGGMLPPEMVQWLNGGEEEPVAGAYSGMFLKEEEGVKGEMQFNSLYNYTTSTLGHTVSSGHMTTTS
SLQKPLSGHMSATALLHKAAQMGSTTSSNQDDHTTGFGLENGVNESHDSELTRDFLGVGGDESRSFNLQQKLFKFSSSMEDSSI
>SrC2H2.t1-Mx.27
MINKLIPEEIIVSNGVTDTNNPSSIPPTIKRKRNLPGTPDPEAEVIALSPKTLMATNRFMCEICGKGFQRDQNLQLHRRGHNLP
WKLKQRTSKEVRKRVYVCPEKSCVHNHPSRALGDLTGIKKHFCRKHGEKKWKCAKCSKCYAVQSDWKAHSKTCGTREYKCDCGT
IFSRRDSFITHRAFCDALAEETARVTASSHFNNLPPSGPVGTLNYHFVGPPVLAGPTMAQHLSSNIFKPVSTNPDTTRQDGLSL
WTSSNNNSNNNTELHNMHQLDYQNLPTHSEYQTNWGVFEAKTTSDHDVNLNTPSLFTTLNNLHQTHPSASMSATALLQKAAQIG
STSSAATTGSSFLINSNNNFNVTTTTVGSATTQVQDHHELCGLYDTSPMINNYRGSGLENEFAGLDEMYPPSKRRHIHIEEHHG
GQTRDFLGVGIQPICHPRIRFDHA
>SrC2H2.1i-Q.28
MSFFNLNLLDNEYDHDSSSSSTLSPRSNPEHRVFPCNYCRRKFDSSQALGGHQNAHKIERNLAKKNRELSSVVKTHSGFNQPAP
RSGRLGGSSHGRPMQAPVGMGINHQEHVGRFGASDLRGGVDYSYKGESVEEDFSQLDLSLRL
>SrC2H2.1i-Q.29
MLNHSDQPLRSIDDQVHDDGQSGLQDERSYECTYCKHGFTNAQALGGHMNVHRKERAKNRLDYSTNNKSSTRLQESSLITNFIH
DHNKQEKKTQEFMLRSSPMMNDSSLRNYENPNGLITCPSREEMRLSLSLSLRFGRSCEDEESIQGGNEDDELDLELRLGQDPWK
CS
>SrC2H2.2i-Q.19
MALEALNSPTTPTPPSVRHTYISGKRQRVETEEEYLAFCLMLLAGGHHPSTDAAAVQHNSPVAYKCSVCNKSFTSYQALGGHKA
SHRKNFPDDHHLDTSTTAARMSNSSVLKPSGKVHECSICHRSFPTGQALGGHKRRHYDGGSASDGAASSTPSQPRDFDLNLPAF
PDFQVESSVDCVGKSQHSIYEQEVESPIPMKKPRLSRTSDSFRTYRF
>SrC2H2.1i-Q.30
MMNSREDRSYEYTKGWLNLSLGQNLGVQSASKPMKVYTCNFCKRKFYSSQALGGHQNAHKRERDAARRYMSPNTTTLAINFRVN
QPLVIQAHSVYHTPSRDAEPTVTRFGDNGSIDVNLVRAYDGEEEQLGLKWPGGIYFEAQTASQPSDHHAIDLNLKL
>SrC2H2.1i-M.17
MNLIHQMNSLCKSIEVLSFNLQPPPLFLHQFHHQSFNPTTSLRQCHRPLPPLLASEHPSSPASISDIDMVPTAEGIYTVKLKKV
VILWDLDNKPPRGPPYAAAMALKTVANHLGEIVDVSAYANRHTFIHLPHWVRDQRRDQRQQDILEKKGVITPTELYICSVCGRK
CKTNLDLKKHFKQLHEREREKKMNRMKSLKGKKRQRFKERFINGDHKYMEAARNLITPKVGYGLAAELRRAGVFVKTVEDKPQA
ADWALKRQMQHSMSRGIDWLVLVSDDSDFVEMLKRARASNLGTVVVGDWDRALGKQADLWVSWNRVENGEITEDDLVIRSRNGG
FVDDVDEDVFVSSFDEQSMNLDGVLDELVRGRKEFNQSKISVFSEGEDDYDDASDYLSDSEDDEEDDWYI
>SrC2H2.1i-M.18
MNLLCKSIEVLSFNLQPPPLFLHQIPHQSFNPTTSLRQCHRPLPPPLASKHPSSPASISDIDMVPTGEGIYTVKPKKVVILWDP
DNKPPRGPPYAAAMALKTAANHLGEIVDVSAYANRHTFIHLPHWVRDQRRDQRQQDILEKKGVITRTELYICNVCGRKCKTNID
LKKHFKQLHEREREKKMNRMKSLKGKKRQRVKERFINGDHKYMEAARNLITPKVGFGLAAELRRAGVFVKTVEDKPQAADWALK
RQMQHSMSRGIDWLVLVSDDSDFVEMLKRARASNLGTVVVGDWDRALGKQADLWVSWNRVENGEITEDEMVIRSRNGGFVDDVD
EDVFVSSFDEQSMNLDGVLDELVRGRKEFSQSKISVFSEGEDDYEDASDYLSDSEDDEEDDWYI
>SrC2H2.1i-Q.31
MSHLDLNYLPQNDFTLDLLHDSSLSSSPSSSSSNPEQPRVFSCNYCSRKFYSSQALGGHQNGHKVERALAKKSRYMNSGVKLWD
QISGCGPDGSNHVGNWVQPPLMNQGHVGRRELGYWYRGETVQEDFHELDLSLRL
>SrC2H2.1i-Q.32
MSHLDLNYLPQNDFTLDLLHDSSLSSSPSSSSSSNPEQPRVFSCNYCSRKFYSSQALGGHQNGHKVERALAKKSRYMNSGVKLW
DQISGCGPDGSNHVGNRVQPPLMNQGHVGRRELGYWYRGDTVQEDFHELDLSLRL
>SrC2H2.1i-Q.33
MEPEQPPAETSDDQKPPFTTTNDQQQNTTHTPRSYECHFCKRGFTNAQALGGHMNIHRKDKNKFKHSSSTARSIPIPLAEPPS
PTGSQPSTTAMGVAYPFSFSHVTPQDEKKPLLALFDHHDTAILAPRNIHEPGNHPLPDADADVDLELRLGHVEPLEENRTTTTR
KFF
>SrC2H2.2i-Q.20
MKRNWDDDREIENLAMANCLMLLNRVNQSGSDSGRVFHCKTCNKQFSSFQALGGHRTSHKKVRSMVESPPKTKTHECSICGLEF
ELGQALGGHMRRHRDSQSEKSVPAKSTVAVVKEDDGGRRGLCLDLNLTPFQNELKVWSRSSGTAGIAT
>SrC2H2.1i-Q.34
MLNHSDQPLRSIDDQVHDDGQPELQDERSYECTYCKHGFTNAQALGGHMNVHRKERAKNRLDYSTNNKSSTRLQESSLLTNCIQ

SIHDHKQEMKTREFMLRSSPMMNDNSLGNYQNPNGLITGPSREEMRLSLSLSLQFGRSCEEESIQGGNEDDELDLELRLGQDPW
KCS
>SrC2H2.1i-Q.51
MDHKFKSKWEGSNFMFHQKDYSWPRRNFTCNFCKKEYKSAQALGGHMNVHRRDKARLGLSSPLSLDHDHHANPNPTYISQPSPP
ARYLPYHTYHSSLFPLVSTSNTKDEQHKLQLIACSPRAIEDGKEEEERGNGTKVWKNYEILNMEMEMGLLKDGNIELDLELRLG
RS
>SrC2H2.t1-Mx.28
MSNLVQDFEEDDVQLHNGDQSSHGSPNTGGIHPPKTLKVKKKRNLPGNPDPNAEVVALSPRTLMATNRYICEVCHKGFQRDQNL
QLHRRGHNLPWKLKQRPANIQVRKRVYICPEPNCVHHEPTRALGDLTGIKKHFCRKHGEKKWKCDKCSKRYAVQSDWKAHAKIC
GTREYHCDCGTIFSRKDSFVTHRAFCDALTERVVESPSTNPSIMMRSSHDKYSFRYNDFDQHAIQVSSETNNMAAYTSATALLQ
KAAEMGAKVSDNTIAPILLRGFTGYSISNNFNEVSSCNTTSGLHAINAEMFDNNIMENYGIINTTTTDKSVLDDSSLRMHSLKG
NRQGFMGGSQKMTVDFLGVEPDSLCGKRDYNGNMMGMHQN
>SrC2H2.t2-M.03
MENQTFPNHFERRAFANSKAPVVKWFKKWVPHDVVTPGGKCFVLKWVTETQLNAMKQNSKELDFVSPEHNPTTEVLFLCSHEGC
GKTFVDVGALRKHSHVHGERQYVCHYDNCGKKFLESSKLKRHFLIHTGERDFVCPHEGCGKAFSLDFNLRSHMKTHSHENYHIC
HFPGCGKRYNYEYKLKNHILSHHQKQNKNNTMVEAPVKYIHIQPVEKPPQKSTKARPYACPYERCEKAYIHEYKLNLHLKREHP
GHILEENTKNALGNVTDASIDHDEHTVKRAGLKSHKQNRPKPDLKPPPAKVIKRNAPFVVNHDLTSVISKQLWVVGKNMYEEDS
EETEEERGNGENRWGYRDINDDDDDDDDNDDDEETEYEY
>SrC2H2.2i-M.04
MEEEMIKMCNIMPVELAIKRELEYRKKMEVLMNQHQIDLNPLISSQVPFSEGQTMADRKRKVDPCNAQCSRPMRSSRSFRSSPR
FVCVVCRIAFATVYHLKIHGETFAHKAKLVSSKREGENTSNPFLCEICDIQCSASRVMESHVAGLKHATNLQEFEDAKRNRIYG
SN
>SrC2H2.4i-Mx.08
MEEDHDEMRFVCKLCDKKYPSGKSLGGHMRSHVIAAATVTATDTTAANSSESDEKFSTLMMDGNGIDNGNPNSYGLRENPKKTW
RAVGSSTFSFPNERICKQCGKGFQSLKALCGHMACHSEKDRNFKDYDHSWTSENLDQDDKLIIDSHSDTEEPEFQDPTRVTRSK
SKRYKKVVVKPYSFNFSITNNDSNNSNYNYGSSSVSETDEIEQEEVAISLMMLSKDSANWGGVNSVVESSDNNSAVLETKSSSV
DMKISKKSSTLDADGFQFDHTTRKLESDITVEELLRNGDPNKKSKRTKELLTYEQKLEIRRNLFKEFEYRELKKRTRDDDDAYN
PELERDEPIKKRNKYECLTCNKIFTSFQGLGGHRPCHKKNNIIASRNDSGDNSLENEYANARKAKYDKKMKAKKSKGHECPICF
RMFKSGQALGGHKRSHFINGSMDHFATMENGAPSCSDMIDLNLPAPEDDQN
>SrC2H2.1i-Q.36
MYNMAEIDHYQEKPTNNTSLKIFGFNVYEDEEVESTKTPTGSLDSGNCFPATDGRKYECQYCCREFANSQALGGHQNAHKKERQ
QLKRAQMQATRNAAVSYIRNPIISAFAPPPHLLTQTGPMVIPSSGVANPSWVYVPRATPAFHVSHGCVLPATSSPSGGRGPGSL
SYAGSVGESSLTNVGPQPNNRAYHEDGPSLSRFSKGDGGPSFDDSFGLDLHLSL
>SrC2H2.t2-M.04
MQTQSSFTNISTSESNYGCLESEEVDKDSILLSLSPPGQQPQHHQLPNYSAHDHHHHHHNVSSPSNSSFHQNPDTDLNINLNLN
LSLDHTSIKSCNDGVTVALRLNHQQNASSSVDANCLNTNPNMNQTPEHLQYWIPTAAQIMVGPTQFSCTVCNKTFNRYNNMQMH
MWGHGSQYRKGSESLRGTRSGSPMMRLPCYCCATGCKNNIDYPRSKPLKDFRTLQTHYKRKHGEKPFACRKCGKPFAVRGDWRT
HEKNCGKLWHCVCGSDFKHKRSLKDHIHAFGNSHAPYYTSDQDLMEVNDQEDENLYEEEKGSSSNGGHDGRFL
>SrC2H2.t2-M.05
MQTQSSFTNISTSESNYGCLESEEVDKDSILLSLSPPGQQPQHHQLPNYSAHDHHHHHHNVSSPSNSSFHQNPDTDLNINLNLN
LSLDHTSIKSCNDGVTVALRLNHQQNASSSVDANCLNTNPNMNQTPEHLQYWIPTAAQIMVGPTQFSCTVCNKTFNRYNNMQMH
MWGHGSQYRKGSESLRGTRSGSPMMRLPCYCCATGCKNNIDYPRSKPLKDFRTLQTHYKRKHGEKPFACRKCGKPFAVRGDWRT
HEKNCGKLWHCVCGSDFKHKRSLKDHIHAFGNSHAPYYTSDQDLMEVNDQEDENLYEEEKGSSSNGGHDGRFL
>SrC2H2.t1-Mx.29
MFENNDHHIHHLTSAPSSSSDPFLSPENGFSSKRKRRPAGTPDPDAEVVSLSPKTLLESDRYVCEICNQGFQRDQNLQMHRRRH
KVPWKLLKRESPEVKKRVFVCPEPSCLHHDPGHALGDLVGIKKHYRRKHSDDKKWVCEKCSKGYAVQSDYKAHLKTCGTRGHSC
DCGRVFSRVESFIEHQDACTVRRVQPDPPTFQPVCSSRSASSTSPSSDTNINTIPSLLQQVPQIPYTQTPFSQAQNMQNNLELR
LLPSSSLYDPYNDNQTTLKLSIGSPKQEEKMKMVMDEKELLAEEVDN
>SrC2H2.t2-Mx.07
MDLFASSSAVYEPFDFFKNKDEEQSFKTIPFFSFLPHEPCKLEVSSRSSTKVATSDQDESLELQIGISSSSDNIIVVDDESIVN

NNNKTVGNMRGYWIPSRAQILAGFSHFACHICDKIFNRYNNLQMHMWGHGSQYRKGAESLRGTQPRVMIGVPCYCCEEECKNNI
NHPRSKPLKDFRTLQTHYKRKHGAKCFSCRKCEKSFAVKGDWRTHEKNCGKRWLCVCGSDFKHKRSLKDHIMSFGLGHTPEPPS
FHDVNHQDQAIN
>SrC2H2.2i-Mx.04
MALLTFPFSSETQKVQPTKQESSRKKQLQQKPKTKPKSKQQNPSSFSSWDQLKNLLTCKQMDVVEPNKVNNGYSKLSSCSSICS
FRDVVHGNTRVVHRAEHSPESSGVGMDSGLLSRNKQVTDGGSSSSSSKILNSSIRHNGHGSYTSSSRGMQFRKLSGCYECQAFV
DPSRYTIPRSTICVCSQCGEIFTKMESLEHHQAVRHAVSELGPDDSGRNIVEIIFKSSWLKPDHPIFTIERILKVHNTRRTIQR
FEDCRDAVKFRATATATASRCAADGNELLRFHCTTVACTLGSRNSSSLCATIPGCSVCTVIRQGFQSTRSGGISGGDGGAIFSG
ISAARDGGSKGGVRTTASSGRAHDCLGVDPHERRAMLVCRVIAGKVKHVTGNVPPEEDGPYDSVAGCAGLYSTLEELYVFNPKA
ILPCFVVIYKAFES
>SrC2H2.t1-Mx.30
MTTDLDYSSAVNESTVSGEASASSPGNQTAVTPEKPVKRKRNLAGMPDPDAEVISLSPNTLMATNRFVCEICNKGFQRDQNLQL
HRRGHNLPWKLKQRTSKEIRKRVYVCPEPSCIHHDPTRALGDLTGIKKHFSRKHGEKKWKCDQCSKKYAVQSDWKAHLKICGTR
EYRCDCGTLFSRRDSFITHRAFCDALAVESAKGQPEDEHLKKDEEPQIIESAPQSTSTASPLAASPPPPLPPPTPAATVTPAVP
VTSSVLCISKTADLPESSNSDHDSRPVVDNPPQSMAGLTGSCSSTNTGTTSSSVFASLFATSTTSKTLQPQPQETGFTNLIQPM
TRPTQTPDLTIPLLSQEPISLCLSTNQGSSIFKSGQYIPPHQPAMSATALLQKAAQMGATGSGSSLLRGYGLERDDPDGGSSIG
LGLGVALTGGLVCKSS
>SrC2H2.1i-Q.37
MEQYSQYLMSMKSNNSKSNNSIVVNPVSSRSWEEQAFAEDARGPLGGFVWPPRSYTCSFCKREFRSAQALGGHMNIHRREKAKF
KQNVNDTTLTTKQNPNKVEILCKESLDLCLFSSRVSLVPTLGQESLDSSSDTKSCVYDEGLVVTHGIVDVETNLGLGFDLDSWA
SNKGADGVKRQKRNHVTPPVVGGLDLELRLASFHAC
>SrC2H2.2i-Q.21
MVYLAMKRSRGDEFDSTITSTANYLILLASGIASTEPYDANPVSRLFECKTCNRQFTSFQALGGHRASHKKPRLVDGDMDGHHD
EVLPSKPKLHKCSICGLEFAIGQALGGHMRRHRAVIATKDRPIPVIDLSSPVVKKVNSRRVFSLDLNLTPIENGFGFRVDDEKA
TPITVDFFL
>SrC2H2.2i-Q.22
MDPQQTTTTTTTSAGDDHGGPKDPTTIVKGKRTKRQRPTIVSHSSTSYNAFNSSPASSSNFPAGSTTAEEEDTANCLILLSKGH
NINPQNNINNNNNNNMEELSGYKFNSKRYIQTSTDAVNGPTGMYVYECKTCSRTFASFQALGGHRASHKKPRINELETRKPPRY
VSEEDEPSSRFELESRRNNFSSSSSLSLQLNHRMVSYNSKKPSSRLHDCSICGAEFNSGQALGGHMRRHRSVNGNATAISAGVI
ATNTTLSLVPYGTVTSVVADDHDNQKSMNDGLCLDLDLNLPAPPEMSVAVSDQDQRRESSFKFTANQNKQQPTVHLSATPTLVD
CHY
>SrC2H2.t1-Mx.31
MMSGEMFHIPLQDHHLHHQPPPPTPHESNPNSKPISNLKKKRNLPGTPDPDAEVMALSPKSLMATNRFVCEICNKGFQRDQNLQ
LHRRGHNLPWKLKQRNNKEPIKKKVYICPEKTCVHHDPSRALGDLTGIKKHFSRKHGEKKWKCEKCSKKYAVQSDWKAHSKTCG
TREYKCDCGTLFSRKDSFITHRAFCDALAEESVRLGSVTSNNLNLKNEGVNESVMNPNFPQGFSRMTQFGSGYGPDFGDQNKPR
LSLWLDQANSEVNISPMDQMTMNSNLYMSSSSAGLADMVNAYGSSSISNFTNSNQVPSLSLTPLTEGLKEETLVSLYSMNQNHQ
SESTGPMSATALLQKAAQMGSTRSNPSIFGSSFGLMNSASTTSNITTNLVNNNITSQLQVSTAMNNNSSNSSPQTSNLDHQHMI
MSRNEAMASLKMMHRGFSGVDNGLTRDFLGMGGEGGRMLLPQDLVKFASNYGTNMGSMRQFASNN
>SrC2H2.t1-Mx.32
MMSGEMFHIPLQDHHLHHQPPLPTPHESNPNSKPISNLKKKRNLPGTPDPDAEVMALSPKSLMATNRFVCEICNKGFQRDQNLQ
LHRRGHNLPWKLKQRNNKEPIKKKVYICPEKTCVHHDPSRALGDLTGIKKHFSRKHGEKKWKCEKCSKKYAVQSDWKAHSKTCG
TREYKCDCGTLFSRKDSFITHRAFCDALAEESVRLGSVTSNNLNLKNEGVNESVMNPNFPQGFSRMTQFGSGYGPDFGDQNKPR
LSLWLDQANSEVNISPMDQMTMNSNLYMSSSSAGLADMVNAYGSSSISNFTNSNQVPSLSLTPLTEGLKEETLVSLYSMKQNHQ
SESTGPMSATALLQKAAQMGSTRSNPSIFGSSFGLMNSASTTSNITTNLVNNNITSQLQVSTAMNNNSSNSSPQTSNLDHQHMI
MSRNEAMASLKMMHQGFSGVDNGLTRDFLGMGGEGGRMLLPQDLVKFASNYGTNMGSMRQFASNN
>SrC2H2.t2-Mx.08
MSSNHPYFNGPWFNLNPNYHYFTSATPSFSSFSHYYQPSPPSPPLREALPLLSLSPTPRSSNTTDSYSTTNMEELDHDKNKLAA
AAADDDDDHETVTVALHLGLPNPSFDLFPRVSTNSDVNNITEADDDYINKHVEEEEGTNGNGYFTSALNKGQYWIPTPAQILIG
PTQFSCPLCFKTFNRYNNMQMHMWGHGSQYRRGPESLRGTQPTAMLRLPCYCCAPGCRNNIDHPRAKPLKDFRTLQTHYKRKHG
IKPFMCRKCGKAFAVRGDWRTHEKNCGKLWYCSCGSDFKHKRSLKDHIKAFGNGHAAYGIDDSCFDLEEEAASEIEQDNESSHC

DN
>SrC2H2.t1-Mx.33
MSNLTLDDSFSSENTKELETQPRNQLSKLQTDFHGGITQLSSAKKKRNLPGTPDPTAEVIALSPSSLMATNRFVCEICSKGFQR
DQNLQLHRRGHNLPWKLKQRTSSDTRKRVYVCPELSCVHHDPTRALGDLTGIKKHYSRKHGEKKWKCDRCLKKYAVQSDWKAHL
KTCGTREYKCDCGTIFSRKDSFITHRAFCDALTEENNKVNQGTLQSQDMLSHYNPSMQMDPRKSMSIGLREFNNYNPESPFKLN
PQENPMFYGSMNMSAGLFSSSSGTLLNNPRGASSSTSGLEPNSSNGPSGFGKFQREIRTGGPISEPMAHMSATALLQKAAEVGA
TTSNFINPPIMQKSYVTSMEGLDQPNYNGPRPSLSYFDTQGPFHNIQTQPELESNVLQKPSRQDTFGPEPKRTLDVDPDMNDIG
IYSEILKGGDQCLGLTNMEHEDETHVSGGGNDTLIVDFLGIG
>SrC2H2.1i-Q.38
MESTTMGDHEDRFINLVEEDDPQNQDVGPVVRSYGCIFCRRGFTTAQALGGHMNIHRKDRAKSRPIYLSGNNSNNQENHSLYAN
HRIYHPVLARCSQTMDNQEGKPYLSTTSSTSQLVNHEKSHQDCYGVIKSPLMEEKTMNLSLQFGWIDGEDNRESRRRTRGGRNE
DEPDLELRLGHDP
>SrC2H2.2i-Mx.05
MANLRPVFTKLTSLLFILIHLGCFIIFPTSDHHRRRRSTTRKPSSPLPLLRSTATKRKSHKPLSTTWTFIKQLFSSRSDANSPI
IHHVPPSPASSTRSLQNSGVTRPGSLTESDFFPLRNDIYPCPNCGEIFQKPGLLDQHLAFKHAVSELFDDDPGKNIVRIIFQTG
WPCNGKNPMIYRVMKIHNCPKILTRFEEYREIVKSTAARYPGTRRRDERCIADGNELLRFHCSTFLCDLGQNGNSSICSHQYCS
VCGIIRVGFSSKMDGISTFSTSWKAHVALPEDIEDEFKFMHVKRAMLVCRVIAGRVGYETESGDKDDPGYDSLVGREPGGTQTR
LDEEDELVVFNPRAVLPCFVIAYTV
>SrC2H2.t2-Mx.09
MTDPYSNLYSQWFRLDQFSTNSPTNHPHHIHSNHPYQHPHPQSYYYNNFHPSSCVNIRTIHNFNLNPSTSFNNQHPTPPPSPPL
REALPLLSSSPSRKQQDYHQQKQPSCSSTTSHQHMDVDGDDVEDEDEDEDDETDVTVALHIGLPKASAADLASLISNSSNSPST
TSEIIVSNINKSKDGDGDEGSIGYSHISRLNKGQYWIPTPSQILVGPTQFSCPVCFKTFNRYNNMQMHMWGHGSQYRKGPESLR
GTQPTGMLRLPCYCCSPGCRNNIDHPRSRPLKDFRTLQTHYKRKHGVKPFMCRKCGKAFAVKGDWRTHEKNCGKLWYCICGSDF
KHKRSLKDHIKAFGHGHAAYGINGFEEEDEPASEIEQDDDDGNS
>SrC2H2.t1-Mx.34
MDGVNKHDSWTKASCSNEINHQLYDSQQQQRGNSDVDDYEVMIRKQLETIPSCLTQINDQRRSQLWDPKTMLSNLSIMEEKIHQ
LQELVQLIVGTTAMDQPNQLVIQQQQLVTADLTSVIIQLISTAGSLLPSVNNPHFPATSTPAFGNEINVPDNKYNENDSNTIKN
IVSKVEDHSNEIDHMDIHEEHDEDVDDGEHLPPGSYEILQLEKEEILAPHTHFCVICGKGFKRDANLRMHMRGHGDEYKTPAAL
AKPHKEIGSEAKLIKRYSCPFIGCKRNKDHKKFQPLKTILCVKNHYKRSHCDKSYTCSRCSTKKFSVIADLKTHEKHCGRDRWL
CSCGTTFSRKDKLFGHISLFQGHTPAIPLDEHKAGLIHDNAEGDVINTNTTIQGSKEVGFFDFNFNSNGSSGPGIGEPSACFSP
FQEFPRSLFEESDNLSFLLSGSGNYLWKNGGQSSSKDL
>SrC2H2.2i-Q.23
MMMYLPMKRPREAELDIAMTNMANCLMLLSERPTSNESFDNAPERVFECKTCNRQFTSFQALGGHRASHKKLRLNEDHGDLTRG
TNLVPMKPKSHECSICGLEFSIGQALGGHMRRHRAIKTNESLSSVSVSDDSSNSVVKKVNSKRVFSLDLNLTPLENDQEFELRN
VVPSTIKLFI
>SrC2H2.1i-Q.39
MEKNAFDLLSSTWEEGDASDQHSTQTYVEKKLKLFGFELHPQADETTIITNHLRSAEGDESVNSSSTTIEKNPSTNLKKFECQY
CFKEFVNSQALGGHQNAHKKERLKKKKLQLQARRARINHYLQPYTHYKHGVTFDFHGYYDPENESNISFSQFDDDLLSFRDPCN
FTLTHVDSLRGNYKSLAIRSPSSRVLDIKQNHVGLDLQLTLSSNSIK
>SrC2H2.t2-M.06
MKAMHEKTENPVNVEPEPEPTTEVLFLCSYEGCGKTFIDAGALRKHSHIHGERQYVCHYENCGKKFLDSSKLKRHFLIHTGERD
FVCPHEGCGKAFSLDFNLRSHMKTHSQENYHICPFQDCGKRYAHEYKLKNHIMSHHEKNVSNLSELPLKYVHQVQPVEKPPLNP
KPPKAAATASYATATSDRPYACPYEGCEKAYIHEYKLNLHLRREHPGHLPEENPKNAPRSKTNTDADMDAGSDHDGQYAVKRGN
TKSQKLTRPKPNIKLPPAKTVKRKPPVAAANAAKKQPWPVVVKPVQEEEDSEETEEDRDNGEDGWRYGGGLDNDDDDEETEYED
>SrC2H2.4i-Mx.09
MDEDHDELRFVCKLCDKRFPSGKSLGGHMRSHVIAAANSAESNDKFDQMNKHSSFIINGIDNANSNSYGLRENPKKSWRAFSSS
STLPSPNEQICKQCGKGFQSLKALCGHMAYHSEKDRNLKDYDYSWTSENLDLDHDQEDKLIEDSYSDTEENPVQDPICVTRSKS
RSYKKVVIKPSSLSLTNTNSTTSNYNYGSSSVSEIDEFGQEEVAMCLMMLSKDTANWVGVNSVVESSDNDSVELETKLCSVEMK
NNEKESYSGYCRNGIKKVESDNSVENQVKGFNYKNLTYEEKQEIRRNLFREFGYDNSLNKRIRNDDSCKKRSKYECLNCNKTFT
SFQGLGGHRPCHKKNNSLSACGNSLKNEYARDQKAKFEKKMRPKKNKGHECPICFRMFKSGQALGGHKRSHFINGYDDKINHMT

VMEQVSATYTDMIDLNLPAPEED
>SrC2H2.1i-D.01
MDEMKDKMKGFMKKFNTSSSSGKFKGQGRVLGSSSSSIPSGPVNSNPNRPTTHIQDRKPVPPPRPETRSPNVLPQKPATSEQPA
KSTNGFDPFDALITTGKRNKNGYDLNVFECPVCNRAFGSEEEVSNHVESCLSVNESQSQLRTDDINEKLETQSELETIVGTYVS
GKPSDGSIEIVLKLLKNIIKDPENVKFRKIRLGNPKIKEAIADVAGGLDLLECVGFELKEENGEMWAVMETAISEKIKLIKQAV
NLLEPPKDEIVPPATAHDKAVEPMEIKKVERQTRVFFSVSESVAAKIELPDSFYKLSIDEVKKEAELRRKKLAESQLLVPKSYK
EKQVKAARKRHEKTVIRIQFPDGVVLQAFFNPREPTTNLYEFVSSSLKDPSLEFELLHPVVIKRRVIPNFGEKVVTFEDEDLVP
SALIKFRPKETDSVVFTGLCNELLEIMEPLVSESAVAQQ
>SrC2H2.1i-Q.40
MAELDHYQATPTKTPSTRLKLFGFNVLEDEESKTRNVSSDSRKYECQYCCREFANSQALGGHQNAHKKERQRLKRAQILATRNQ
HHVLTHQTAPVTHGGRMILPANRFTSPSWVVPRVVNPIQLESRGCILADTSCADDRPNIGADRGLTDGDNGHGLDDSFGLDLHL
RL
>SrC2H2.1i-Q.41
MHVMEVRSEEKDDVVDGLTSQKWKNIFLHLGNPLDLNNFPDDFTKDNGKRPLDDDSSSSASRIIGKKKNGAKDESDKVYECRFC
SLKFCKSQALGGHMNRHRQERETEALNHARQLVFSNDNLIPPFRHQLGGQPMVHGGFHHQSVLCNINSSSTVYPTKPFHDTSTT
MLPPPPPPPPHIYTSPASRLSNHPYSSQTPPLHPLNDYVVGHACSDNPPPFILQNSNRPTAPPPDRTNNYTCIGTPVGQSFTLA
GGNGGGTEMSKSPVTRYHQDGFK
>SrC2H2.t1-Mx.35
MLESNHHHFTAASGSGPGLGFPLSLDVFHSDNGLPNKRKRRPAGTPDPDAEVVSLSPKTLLESDQYVCEICNQGFQRDQNLQMH
RRRHKVTWKLLKRDSTEIKKRVFVCPEPSCLHHDPNHALGDLVGIKKHFRRKHSNNKQWVCDKCSKGYAVQSDYKAHLKTCGTR
GHSCDCGRVFSRVESFIEHQDACTIRLTHADLPAFKPACSSLTASSSSQIISRLPYLQPPPQTIFPQIHVGINNNLELQLLPSL
SLSEQNIINHQTHLNLSIGNGHRFQKEEMKMAMADKFFAEDARQQAKKQIEIAEMEFENAKRIRQQAQVELERAKVLHKQATKK
IDSIILEITCYSCRQRFESFSHNADENSIAPSYMSSALTEGEGY
>SrC2H2.1i-M.19
MAVLQLPPSETTHKLQPTKRRRKHHSQNPKTKQQHPSSSSSSSSSSWDQIKNLLKCKQMDVTGSDQKVQQDPVRNPNGYSKLSS
CSSICSFRDVANGNTRVVHRADNSPESSTGDQDSGSILRRKKHLSNGVSSSSSSSSNTRSLTSSGKSNGQGSYTSSSRGMQFRK
LSGCYECHAIVDPARIAIPRSTICLCSECGEVFPKMESLEHHQAVRHAVSELGPEDSGRNIVEIIFKSSWLNKDHPIFTIERIL
KVNNTRRTIQRFEDCRNTVKLRATTTSRCAADGNELLRFHCTTIACTLGSNGSSTLCGSTTTCGVCTVIRHGFQTPITGDGGRK
GVCTTAGSGRAHDSMVVGKDALRAMLVCRVIAGKVKLIAGNGPPEEEGPYDSVAGQPGSYSSIEELYVYNPKAILPCFVVVYKA
MEC
>SrC2H2.1i-Q.42
MEQGSLTGMEQKGKAKVEDMQEQMPNLKNDARIVVEEHPEKESPLPVQPSPMVLVNSGGHDAASAMSVSNPPPKVANDGEISSQ
KRSSFPCNHCKTRFKTAQALGGHQNTHRYVREKVKKQKLKHTSANSSLIRQPRVIYPNYVGSSNSMTRGLPRIPSYNSNLIPTM
PAFHEYSHSMLRYQATQYPQSTTGLTLGQPRQTSGMLFDGRQSPVVNSSRLISPNIVNNINVNNYTGDNFNFGVGGQGGILGDF
EAPNFNFNSSGIRGRGSTNLDFLRMRSCNNYYSPFGVEATCTSRRAFRNFHGVSQGESNVRGHEDEIMGDGTIDLLSRVGMRNG
EDEERNGDNFKRW
>SrC2H2.1i-Q.43
MMWNPNSMTVDRQEEDDSWEVKAFAEDTGNAMGTTWPPRSYTCTFCRREFRSAQALGGHMNVHRRDRARLHQAQNFKNPNLSFS
GSGSGSGSSTLLIPTDQQLVADGGLCLLFSLPNPNRMFKPSSSSRVLSVTPHQSSGNLMNFPAAVTPPHSFNYSPSNSSNNEAS
TSIDHIHNQRISIENKRMKTDLTVDQGIDLELRLGCSSSGS
>SrC2H2.1i-M.20
MNTETKKKKSVSHQNGDYVCKTCKKRFDTYQALGGHQGTHKRLKSSLGLHQCKTCMKEFETGQALGGHMRRHRIEKALNLMEKE
HIWRQQVAVTADGDLVLAAGEPQQKEEDNRHEDKSELDNRHEDKSELDNPHGDKSELDNRHEDKSELVLATKESCLWRQEAVAK
AESDSVFGAVEPQWVKVEEDKPPPEDKTKLILAAKEWRLSV
>SrC2H2.4i-M.01
MHKPFISIFLSIQKMSFPLSKNTLGLTCHACNKEFIDKNAQKIHYKSEWHRYNLKRKSVGVPGVTQALFLARQSALTDEKNKLY
EPPMLYTCGLCGKGYKSSNAYNQHLKTRAHNTWASQANNQNEITAIIKPLFSKYPQHEDEESDEWEEFDQNVDFSDLNHMEMDE
LSSDVDEDEDELDPTCCFMCDQEHETIESCMIHLHKNHGFFIPDVEYLKDPVGFLTYIGLMVKRDYMCLYCSSNCQPFNSLEAV
RKHMVAKSHCRVHYGDDDDDEAEAELDEFYDYSSSYVDGNGNQLVTPDVSGDVIQLGGIGSELVITRLTDDGICTKAIGFREYS
RYYRQKPRPSNALAARYTSMRLSTVQSKEKMVRMKVMKKMNRSGWR

>SrC2H2.2i-Q.24
MKRNWQDDREIETLAMANCLMILNHVNGSGSSSRFGRAFNCKTCNKQFSSFQALGGHSTSHKKLKSVDESNLKQAKTHKCSVCG
LEFNIGQALGGHMRLHRHSQAEKSVLAKVPVVEEVVGSMRGLCLDLNLTPFQNELKSGLLQELLFKIQNKTNEYDH
>SrC2H2.1i-Q.44
MEPEQPPSETSDDAKPPSAATEQQQQQPTTHVPRSYECNFCKRGFTNAQALGGHMNIHRKDKTKFKHSTTTTTAAAAPANITVA
EPPSTTSSRLNPNPFSISHQGKQLPLFEHDTVVQRNIHHPGGDTSAPEGGVDLELRLGHVEPPPESSSENKTTTTRKFF
>SrC2H2.1i-Q.45
MSFLDLNLPKNYCTLDLLHESSISSSTSNSEQRVFSCNYCRRKFHSSQALGGHQNAHKLERTLAKKTRYLSSEVKLWDHDSDHV
VWVLPSDHVWVQPSFMNQGRVQEDIHQLDLSLRL
>SrC2H2.1i-Q.46
MKTREEISQEKTKGWLNLSLGKKLMKLYTCKFCQRKFCSSQALGGHQNAHKRERNASRRYFSPNTTNLAITFMVNHSLEVQPHS
PAIAWFGGDGTNGVNWVRANDGEEMVGLKWPGGIYFAVETASQSSDDQHMLDLNLKL
>SrC2H2.2i-Q.25
MALEAITTTTAPPSLRHQQMDVQDSWNKGKRSKRPRAATDDDEYLALCLMLLSRGSPSAAVSPPQHSYSPPANYNCSVCNKSFS
SYQALGGHKASHRKNVPDDHTSTSATTTTTAGASASSVLKPDGRSHECSICHRSFPTGQALGGHKRRHYDGNNPGSGVTTSDGA
ASSARSQTRDFDLNLPAFTDFQMGLSVDCGKKSQLSINEQEVESPLPMKKPRLSLMAD
>SrC2H2.1i-Q.47
MMNNNQSKDENKWLDLSLGQTITASCSSQSRPVSVKVCHFCKRKFYSAQALGGHQNAHKKERDAARRYHSLNALSYDRVGVQLQ
AHSLVHKPIRETTVVEVGGGDARVHGEERMWHGGYYSDPRMGSQPSDQDPIELDLNLKL
>SrC2H2.4i-Mx.11
MDDQNTNFKHFCRICKKGFMCGRALGGHMRAHGIGDDTGILDDEDPASDWEDKQGNKRMYALRTNPNRLKSCRVCENCGKEFLS
WKSFLEHKKCSSDDGESLVSSPESEADEDDGYDDENHGARRDCQSSGWSKRKRSLRAKVGSFNSNCPSSEDEDLVLAKCLMELS
NGRVDPAETDLEDSCTSPSREEQRQNPMVPMTTLSPPFARAPPPLDYKAKGVATTPKGMFACKACKKVFTSHQALGGHRASHKK
VKGCFAARNDQSDDNVGDDDVITHDELYPSPKPISSYQFNQGPSTGPSVGLARQISKVHKCSICGHKRCHWLTSNMSDTSSIAK
FNFHEHIEQLHRRALALPSRILDKSKALDLNQPALEQAGTGLRKDPRYPLSFEVSTDINLHSWNVDHKIVTDGEVKDQKRTGDY
QDHDQKASNNNEKEAVLGAIMEDDEADSKLKLAKLSELKDMSNISGSSSSWLQVGIGSTTDVGSSHDP
>SrC2H2.t2-Mx.10
FNCGEAANFGTPQWLSVAKEAAVRRAAMNFLPMLSHQQLLYLLTMSFIPRVPRSLLPGIRTSRLKDRQKEKRELLVKKEFIDDL
LKENKLLNNILQKNPSYHAVLWDLETLSPSVIKESVVTNNENVQIQKEDYLDPETLADIEDDDMSSDFQIDSGTLPCVACGVLG
YPFMSVIQPSTKAVIENITVTGHGFVQPHAIETESVKVDKNWNMSNVYLRPRIFCLEHACKIEELLDSMGGAKLLIICHSDFQK
IKVQASTIAEQIGSAFRYNEVQLNDATQDDLDLINFAIDNEQKEDESVKEDWTVKLNVNLRHSVKLRPKLSTDKIHHALSLTMD
ALFADTTRASSVGASATVLKWEATKFRSKRKSNWSVKKLSNSEKDVEFMEKLSEPQMIKKEKVLIHYSRRKLKSKPQDLVTDFS
KNTNDVGFLVSSDLSTVGQSDIAAIIENDGIEKDDINNETCMQNENNGGSSTDECDTDSNDGVKKNDDNTLESNSTGGNKRKRE
LELLQTNENSAFGGFIKSPCEGLRPRGCKDLHKVGIFINKKPIPEKPTKKSRNPNVTERPSAQPNGSNARNPEKTDHRAHRCNH
DGCKMSFKTKTEVILHRKNRCPHEGCGKKFSSHRYAVLHFRVHEDSRPLKCSWKGCKMTFKWAWARTEHLRVHTGERPYKCKVE
GCGLTFRFVSDFSRHRRKTGHIVTVKS
>SrC2H2.t2-Mx.11
MEEDQDELRFVCKLCDKRYPSGKSLGGHMRSHVIAANSSESDEKFTTLMTNGNGIETANGNENPNSYGLRENPKKTWKTVDSST
IPYSNEKICKQCGKGFQSLKALCGHMACHSEKDRNFKDYDHSWTSENLDHDEKVVIDSYSDIEDPEFQDPARVTRSKSKRYMKV
IVKPYSFNYNYGSSSVSEIDELEQEDVAMCLMMLSKDSSNWGGVNSVVETSDNDSGFLEMNSDSRNLFKEFAYRDSLRKNTRVE
DDSYTPELEREGSNKKRNKYECLNCNKIFTSFQGLGGHRPCHKRTILSALGGHKRSHFLNRGIDHYAVMEHEAPSCSDMIDLNL
PAPEEE
>SrC2H2.t1-Mx.36
MMSSDELSSPPLSHTPFIHDPITNPNPNSSNSVTRKRNLPGNPDPDAEVIALSPESLMAKNRFVCEICSKGFQRDQNLQLHRRG
HNLPWKLKQRNKDEVVRKKVYICPEKSCVHHHPSRALGDLTGVKKHFSRKHGEKKWKCEKCLKKYAVQSDWKAHSKICGTREYK
CDCGTIFSRKDSFITHRAFCDVIAEDNSRMASFAIATTMNLNFQNNWLMMNNGGTIMNPVMKPVLPIWLHQRDNNLIENATYCW
SFLGSSSLMNNNINGGILSPIEMLQGLNKGHETTRSFGEMTHGLLLKEEDNTKGEIKLNPSYNNYGGYSNPPPTPPHMSATALL
QKASQMGSMRSTNSGGFGLMNTSEFTDFNSLTRTENLMMNEDGLMMMMMARSEVDTGDGVLTRDFLGVQGNERREVGLQQELIN
SWVGWWLRSWVGSWFGHGSCVVAWFKLWVGSWFGSWFETWVCSWVE
>SrC2H2.t1-Mx.37

MMSSDELSSPPLSHTPFIHDPITNPNPNSSNSVTRKRNLPGNPDPDAEVIALSPESLMAKNRFVCEICSKGFQRDQNLQLHRRG
HNLPWKLKQRNKDEVVRKKVYICPEKSCVHHHPSRALGDLTGVKKHFSRKHGEKKWKCEKCLKKYAVQSDWKAHSKICGTREYK
CDCGTIFSRKDSFITHRAFCDVIAEDNSRMASFAIATTMNLNFQNNWLMMNNGGTIMNPVMKPVLPIWLHQRDNNLIENATYCS
SFLGSSSLMNNNINGGILSPIEMLQGLNKGHETTRSFGEMTHGLLLKEEDNTKGEIKLNPSYNNYGGYSNPPPTPPHMSATALL
QKASQMGSMRSTNSGGFGLMNTSEFTDFNSLTRTENLMMNEDGLMMMMMARSEVDTGDGVLTRDFLGVQGNERREVGLQQELIK
FSCSMNDSNIGFSGSQ
>SrC2H2.1i-Q.48
MEKSNRETHDFMNVESFSQLPFVRPLSLKEKGIRLFGKEFGSDQTGDLTPPGSPPMNLTQSTTQILQIFISRKFECHYCCRNFP
TSQALGGHQNAHKRERQLAKRAHLQSTMVNGSFSEAQMYGLMNYHRFTTTPPSFYHQTTTSSTTTNYNNRFNYGHNSSYSSHQT
PINGSPLALWKYPNYAQNRMINTSPLISSSNEGLRASRIQTNSSYMYDSKPSVQDQVSLDLRL
>SrC2H2.2i-Q.26
MALEALNSPATTTVAPPPSFHYKDMSFPTTTVLQDSWNKGKRSKRPRATASTESDVTHPSSEPEHPTEEEYLAFCLMLLSRGGN
KPVAATESVAAAPAVKLPPLPQSVSHKCSVCNKEFPSYQALGGHKASHRKNVSDDHPSTSATTTASATTFSTLKPSGRAHECTI
CHRTFPTGQALGGHKRRHYDGNNSGTGPAASGITTSGKTTSSSNSQPRNFDLNLPAFPEFHLGLNVDFVKKGPLFIHEQEVESP
HPAKKQRLSDAGDVPIY
>SrC2H2.1i-Q.49
MGSDQKHGDSETSSDQETSRPLSPNSSKDGLGRSYECTFCKRGFTNAQALGGHMNIHRKDKAKAKQHTTASAKLNKDHRLIITS
SKSFSHDPIEDSQYLGSSFAIRTSDDHGYQFIPSNPNFQWALAASQFDHNRALHEEHLRVNLNLGIGSSETENNINIESTKVGI
NEDWFENGVDLELRLGHYP
>SrC2H2.1i-M.21
MSTVWFSLKKSFHCKSGSSDVHDPKSRNHLSPILTRRPGRCGCSKSIANLKDVINGGSKRHSEKLVNCSPRSIGSSEFLNPITH
EVILDNSTCELKITSFSGGFHDGFALLPETPTSGGVPTMQQFKNTPPKRKMESLDGNGIGNSGHLGIFGKSMNNTVLQKARRSS
EKDSGGCTGGGVTCHKCGKQFRNLENLEAHHLSKHAVTELTEGDSSRKIVEIICRSGWLKSENSSSRIEKILKVHNMQKTIARF
EEYREFVKTKASKLPKKHPRCLADGNELLRFYGATIACSLGINGTSSLCDFNKCCVCQIIRDGFSTKKELNGGIGVFTSSTSVK
AFQSIEVFEEEPNIRKALIVCRVIAGRVHRPLENIQEITNQTGFDSLAGKVGLYSNIEELYLLSPRALLPCFVVIGKP
>SrC2H2.1i-Q.50
MALEALNSPTAPPTPLFRQDSFNHLRYLKSSTKGKRSKRSRIDQPPTEEEYLALCLMLLARGDSRSSNSTIQPIHHTKSNSVTV
SFGSYQALGGHKASHRKNTAGGGDVEQSSVVTTTSTTTSGGRIHECSICHRGFPTGQALGGHKRLHYDGGHVSTGVTSSNHSQR
GFDLNLPASPENVFADEEVESPHPAKKSRLFNPAN
