# Supplementary material for: Genome-wide analysis of the C2H2-ZFP gene family in Stevia rebaudiana reveals involvement in abiotic stress response
Source: Sci Rep. 2024 Mar 14;14:6164. doi: 10.1038/s41598-024-56624-y (PMC10940304; doi:10.1038/s41598-024-56624-y)
Supplement: Supplementary file 12 — Supplementary Figure S3. [file 41598_2024_56624_MOESM12_ESM.pdf]

Genome-wide analysis of the C2H2-ZFP gene family in *Stevia rebaudiana* reveals involvement in abiotic stress response

Shahla Nikraftar, Rahman Ebrahimzadegan, Mohammad Majdi, Ghader Mirzaghaderi

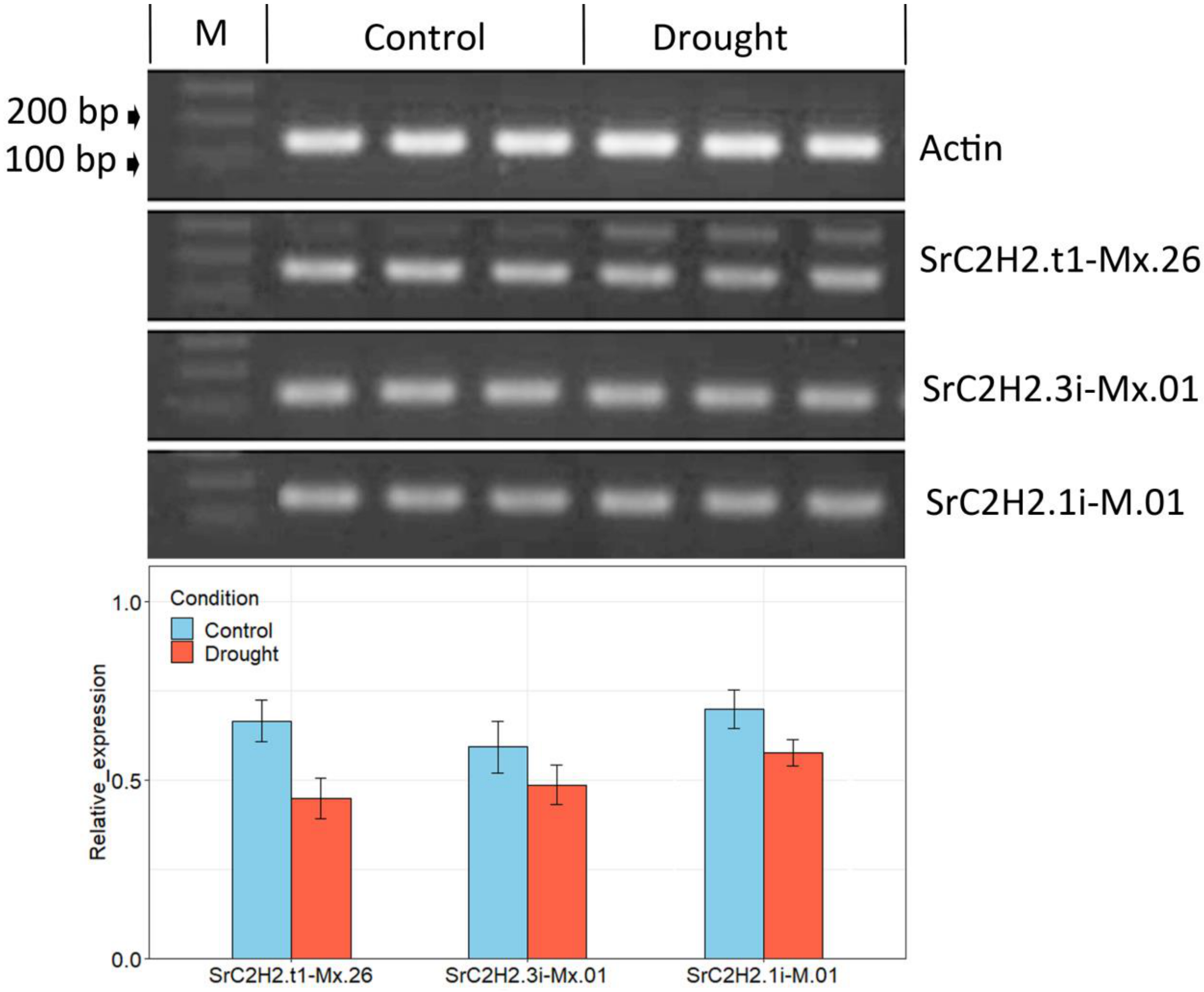

Supplementary Figure S3. Semi quantitative-RT-PCR analysis of three *SrC2H2-ZFP* genes in leaves of *S. rebaudiana.* The barplots of the expressions are shown relative to the reference gene*Actin*. The primers sequences presented in supplementary dataset S7.

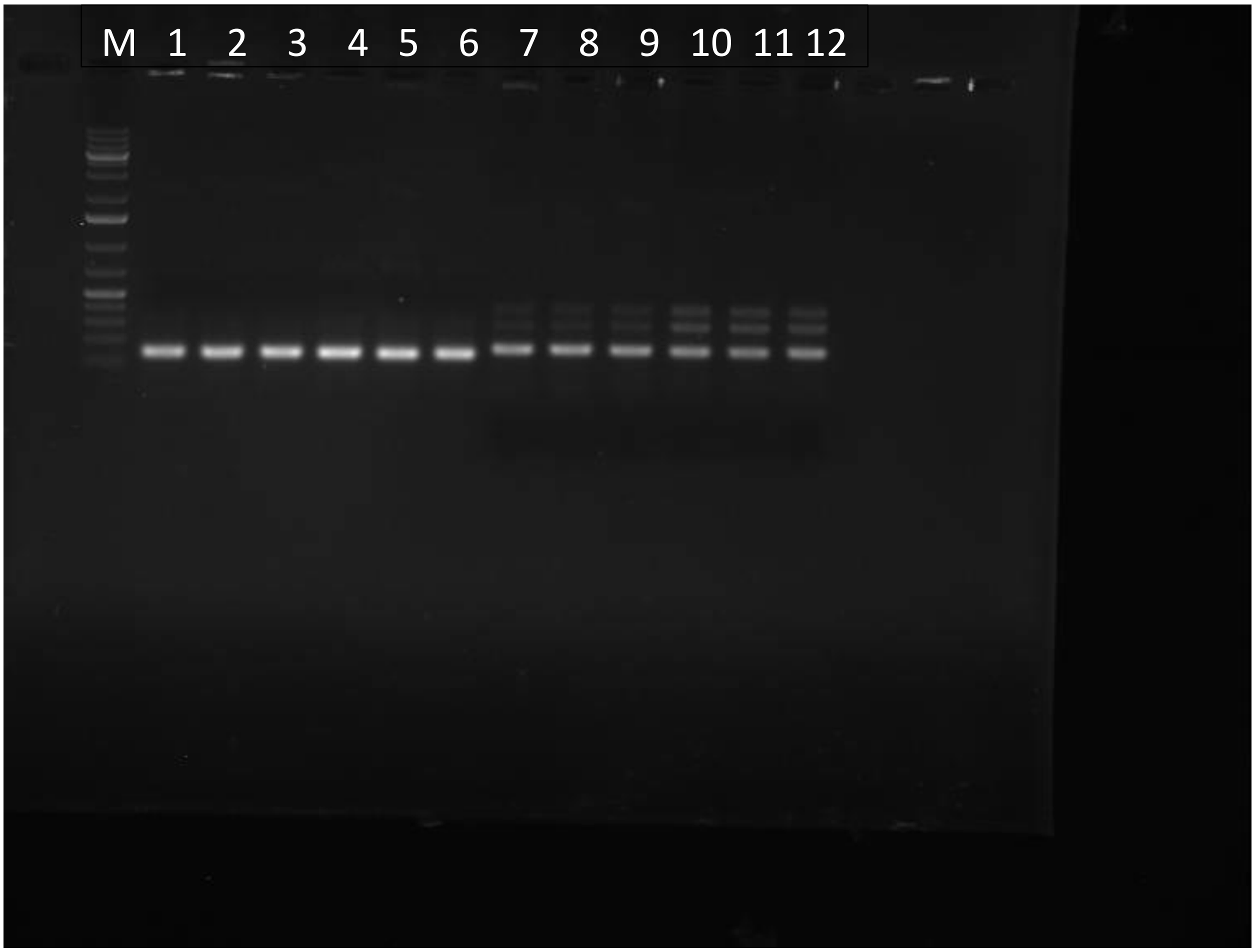

SemiqRT-PCR results for Actin (columns 1 to 6) SrC2H2.t1-Mx.26 (columns 7 to 12).

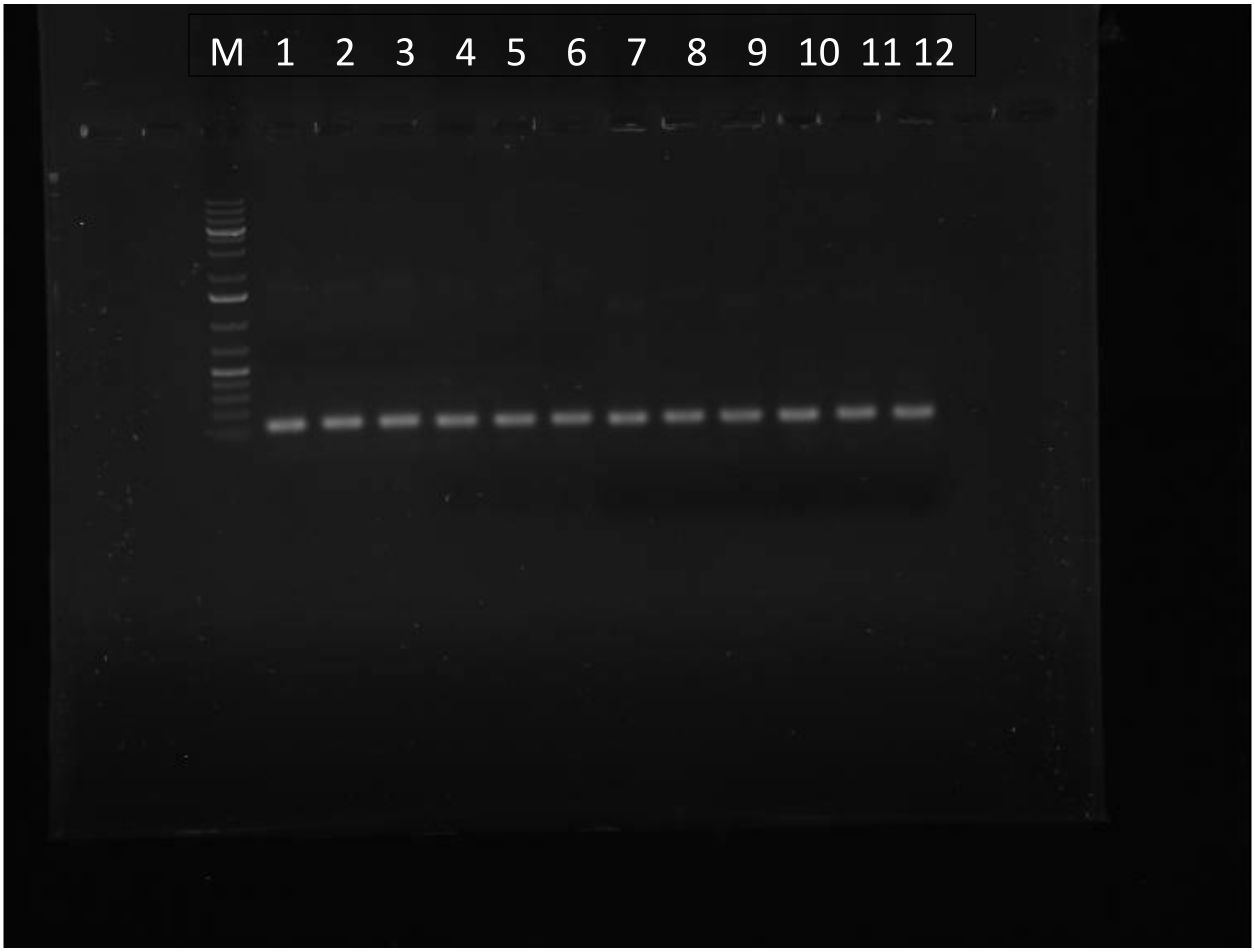

SemiqRT-PCR results for SrC2H2.3i-Mx.01 (columns 1 to 6) SrC2H2.1i-M.01 (columns 7 to 12).
